# Metagenomic sequencing suggests a diversity of RNA interference-like responses to viruses across multicellular eukaryotes

**DOI:** 10.1371/journal.pgen.1007533

**Published:** 2018-07-30

**Authors:** Fergal M. Waldron, Graham N. Stone, Darren J. Obbard

**Affiliations:** 1 Institute of Evolutionary Biology, University of Edinburgh, Ashworth Laboratories, Edinburgh, United Kingdom; 2 Centre for Immunity Infection and Evolution, University of Edinburgh, Ashworth Laboratories, Edinburgh, United Kingdom; University of Cambridge, UNITED KINGDOM

## Abstract

RNA interference (RNAi)-related pathways target viruses and transposable element (TE) transcripts in plants, fungi, and ecdysozoans (nematodes and arthropods), giving protection against infection and transmission. In each case, this produces abundant TE and virus-derived 20-30nt small RNAs, which provide a characteristic signature of RNAi-mediated defence. The broad phylogenetic distribution of the Argonaute and Dicer-family genes that mediate these pathways suggests that defensive RNAi is ancient, and probably shared by most animal (metazoan) phyla. Indeed, while vertebrates had been thought an exception, it has recently been argued that mammals also possess an antiviral RNAi pathway, although its immunological relevance is currently uncertain and the viral small RNAs (viRNAs) are not easily detectable. Here we use a metagenomic approach to test for the presence of viRNAs in five species from divergent animal phyla (Porifera, Cnidaria, Echinodermata, Mollusca, and Annelida), and in a brown alga—which represents an independent origin of multicellularity from plants, fungi, and animals. We use metagenomic RNA sequencing to identify around 80 virus-like contigs in these lineages, and small RNA sequencing to identify viRNAs derived from those viruses. We identified 21U small RNAs derived from an RNA virus in the brown alga, reminiscent of plant and fungal viRNAs, despite the deep divergence between these lineages. However, contrary to our expectations, we were unable to identify canonical (i.e. *Drosophila-* or nematode-like) viRNAs in any of the animals, despite the widespread presence of abundant micro-RNAs, and somatic transposon-derived piwi-interacting RNAs. We did identify a distinctive group of small RNAs derived from RNA viruses in the mollusc. However, unlike ecdysozoan viRNAs, these had a piRNA-like length distribution but lacked key signatures of piRNA biogenesis. We also identified primary piRNAs derived from putatively endogenous copies of DNA viruses in the cnidarian and the echinoderm, and an endogenous RNA virus in the mollusc. The absence of canonical virus-derived small RNAs from our samples may suggest that the majority of animal phyla lack an antiviral RNAi response. Alternatively, these phyla could possess an antiviral RNAi response resembling that reported for vertebrates, with cryptic viRNAs not detectable through simple metagenomic sequencing of wild-type individuals. In either case, our findings show that the antiviral RNAi responses of arthropods and nematodes, which are highly divergent from each other and from that of plants and fungi, are also highly diverged from the most likely ancestral metazoan state.

## Author summary

The presence of abundant virus-derived small RNAs in infected plants, fungi, nematodes, and arthropods suggests that Dicer-dependent antiviral RNAi is an ancient and conserved defence. Using metagenomic sequencing from wild-caught organisms we find that antiviral RNAi is variable across animals. We identify a distinctive group of virus-derived small RNAs in a mollusc, which have a piRNA-like length distribution but lack key signatures of piRNA biogenesis. We also report a group of 21U virus-derived small RNAs in a brown alga, which represents an origin of multicellularity separate from that of plants, fungi, and animals. The absence of virus-derived small RNAs from most of our animal samples may suggest either that the majority of animal phyla lack an antiviral RNAi response, or that these phyla possess an antiviral RNAi response resembling that reported for vertebrates, which is not detectable through simple metagenomic sequencing of wild-type individuals. In addition, we report abundant somatic piRNAs across anciently divergent animals suggesting that this is the ancestral state in Bilateria. Our study challenges a widely-held assumption that most invertebrates possess an antiviral RNAi pathway likely similar to that seen in *Drosophila*, other arthropods, and nematodes.

## Introduction

RNA interference-related (RNAi) pathways provide an important line of defence against parasitic nucleic acids in plants, fungi, and most animals [1–5]. In plants and fungi, which lack a distinct germline, Dicer and Argonaute-dependent RNAi responses suppress the expression and replication of viruses and transposable elements (TEs) through a combination of target cleavage and/or heterochromatin induction [6,7]. This gives rise to a characteristic signature of short interfering RNAs (siRNAs) derived from both TEs and viruses [8–12]. In contrast, the best-studied animal (metazoan) lineages display two distinct signatures of defensive RNAi. First, reminiscent of plants and fungi, arthropods and nematodes exhibit a highly active Dicer-dependent antiviral pathway that is characterised by copious virus-derived siRNAs (viRNAs) peaking sharply in length between 20nt (e.g. Lepidoptera) and 22nt (e.g. Hymenoptera). These are cleaved from double-stranded viral RNA by Dicer, and loaded into an Argonaute-containing complex that targets virus genomes and transcripts via sequence complementarity [13,14]. Second, and in contrast to plants and fungi, animals also possess a Piwi-dependent (piRNA) pathway that provides a defence against TEs in germline (*Drosophila* and vertebrates) and/or somatic cells (e.g. [15–18]). This pathway is usually characterised by a broad peak of 26-30nt small RNAs bound by Piwi-family Argonaute proteins, and comprises both 5'U primary piRNAs cleaved from long ‘piRNA cluster’ transcripts by homologs of *Drosophila* Zucchini[19], and secondary piRNAs generated by ‘Ping-Pong’ amplification. This pathway is thought to target TE transcripts for cleavage and genomic copies for heterochromatin induction in most animals [20].

The presence of abundant viRNAs in infected plants, fungi, nematodes, and arthropods suggests that Dicer-dependent antiviral RNAi is an ancient and conserved defence [1,2]. However, RNAi has been entirely lost in lineages such as *Plasmodium* [21], some trypanosomes [22], and some *Saccharomyces* [23], and/or extensively modified in others. For example, antiviral RNAi was long thought to be absent from vertebrates [24,25], at least in part because their viRNAs cannot easily be detected by high-throughput sequencing of the total small-RNA pool from wild-type individuals [25–30]. Recently, it has been suggested that vertebrates also possess a functional virus-targeting RNAi pathway in tissues lacking an interferon response [31–33] and/or in the absence of viral suppressor of RNAi [32,34,35]. However, there is still debate as to whether this occurs under natural conditions, and whether or not it represents an immunologically relevant defence (compare [36,37]).

**Fig 1:**
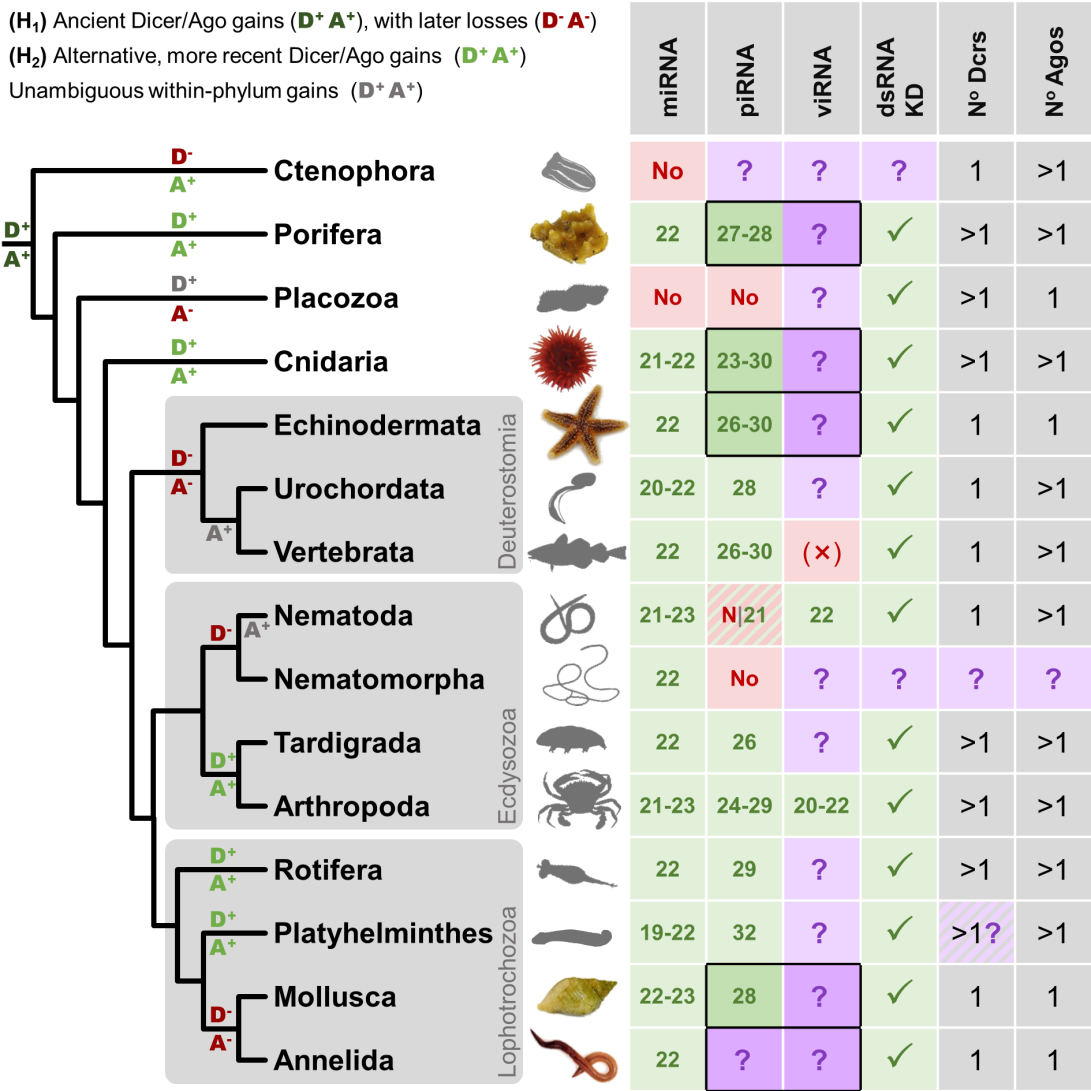
Distribution of small RNA pathways across the Metazoa. Phylogeny of selected metazoan (animal) phyla (topology follows [180]) with a table recording the reported range of modal lengths for miRNAs, piRNAs, and viRNAs detectable by bulk sequencing from wild-type organisms (miRNA modes taken from miRbase). Entries marked ‘No’ have been reported to be absent, and those marked ‘?’ are untested. Focal taxa in this study are marked in colour, and the target table entries are outlined. Vertebrate viRNAs are marked ‘(×)’ as mammalian virus-derived small RNAs are only detectable in tissues and experimental systems lacking viral suppressors of RNAi and/or an interferon response [31–35]. Note that piRNAs are absent from some, but not all, nematodes [57]. The column ‘dsRNA KD’ records whether dsRNA knockdown of gene expression using long dsRNA (i.e. a Dicer substrate) has been reported, as this may suggest the presence of an RNAi pathway capable of producing viRNAs from replicating viruses. The ‘Dcrs’ and ‘Agos’ columns record the inferred number of Dicers and (non-Piwi) Argonautes ancestrally present in each phylum, although the number of Dicers in Platyhelminthes is contentious as the putative second Dicer lacks the majority of expected Dicer domains. Broadly speaking, there are two competing hypotheses for the histories of Dicers and (non-Piwi) Argonautes in animals [47,50,181]. The first (labelled H_1_), posits that an early duplication in Dicer and/or Argonaute (marked D^+^ and A^+^ in dark green on the phylogeny) gave rise to at least two very divergent homologues of each gene in the lineage leading to the Metazoa, followed by subsequent losses (D^-^ and A^-^ in dark red). The second (H_2_), suggests that divergent homologues are the result of more recent duplications (D^+^ and A^+^ in pale green), and where homologs have high divergence it is as a result of rapid evolution. Note that these hypotheses are independent for Argonautes and Dicers, and one may be ancient but the other recent. For Dicers, at least, the ‘ancient’ duplication is arguably better supported [47], although it remains extremely difficult to determine orthology between the duplicates. In addition, Dicers and Argonautes have unambiguously diversified within some phyla (important examples marked A^+^ and D^+^ in grey)—as seen for the large nematode-specific WAGO clade of Argonautes (reviewed in [141]), and the multiple Argonautes in vertebrates.

Despite this clear interest in the phylogenetic distribution of antiviral RNAi, comprehensive experimental studies of antiviral RNAi in animals are not available. Instead, studies have focussed on arthropods such as insects (reviewed in [38,39]), crustaceans ([40], and reviewed in [41]), chelicerates [42], and on nematodes [43–45] and vertebrates [25,26,28,29,31–35,46]. In particular, there have been few attempts to identify viRNAs in ‘early-branching’ animal lineages such as Porifera or Cnidaria, in divergent Deuterostome lineages such as Echinodermata or Urochordata, or in Lophotrochozoa (including the large phyla Annelida and Mollusca; See Fig 1 for the known distribution of RNAi-pathways across the Metazoa).

Broadly consistent with a wide distribution of antiviral RNAi, Argonaute and Dicer genes are detectable in most animal genomes (Fig 1; [47–50]). However, while Dicer and Argonaute genes would be necessary for an antiviral RNAi response, their presence is insufficient to demonstrate one, for two reasons. First, these genes also have non-defensive roles such as transcription regulation through miRNAs (see [51,52])—and a single gene can fulfil multiple roles. For example, whereas in *Drosophila* there is a distinction between the Dcr2-Ago2 antiviral pathway and the Ago1-Dcr1 micro-RNA (miRNA) pathway [53], in *C*. *elegans* a single Dicer is required for the biogenesis of both miRNAs and viRNAs [Fig 1; [43,54,55]). Second, RNAi pathways are labile over evolutionary timescales, with regular gene duplication, loss, and change of function [18,56–58]. For example, the Piwi-family Argonaute genes that mediate anti-TE defence in animals were ancestrally present in eukaryotes, but were lost independently in plants, fungi, brown algae, most nematodes, and dust mites [2,47,57–59]. In contrast, non-Piwi Argonautes were lost in many alveolates, excavates and Amoebozoa [59,60] while Piwi genes were retained in these lineages. At the same time, new RNAi mechanisms have arisen, such as the 22G RNAs of nematodes [57,61,62], the recent gain of an antiviral role for Piwi in *Aedes* mosquitoes [63,64], and the RNAi-mediated immune memory of some dipterans [65–67]. Taken together, the potential for multiple functions, and for gains and losses of function, make it challenging to confidently predict the phylogenetic distribution of antiviral RNAi from the distribution of the required genes alone (see [49]).

Thus, although antiviral RNAi is predicted to be shared by most extant eukaryotes (see [68,69]), in the absence of experimental studies, its distribution across animal phyla remains largely unknown (Fig 1). This contrasts sharply with our knowledge of other RNAi-related pathways, such as the miRNA mediated control of gene expression, which is conserved across plants, brown algae, fungi, and almost all animals [70], and the presence of TE-derived piRNAs in most animals: Porifera [15,71], Cnidaria [15,72], Ctenophora [73], Vertebrata [74,75], Arthropoda [76–79], some Nematoda [57,80], Platyhelminthes [81], but not Placozoa [15]. In eukaryotes that lack direct experimental evidence for viRNAs, the presence of an inducible RNAi response to experimentally applied long double-stranded RNA might indicate a potential for antiviral RNAi (Fig 1). This has been reported for Excavata [82], Heterkonta [83] Amoebozoa [84], trypanosomes [85], and among animals in Porifera [86], Cnidaria [87], Placozoa [88], Arthropoda [89], Nematoda [90], and several lineages of Lophotrochozoa including planarian flatworms [91], bivalve molluscs [92], rotifers [93] and annelids [94].

Thus, although circumstantial evidence suggests a near-universal potential for antiviral RNAi in animals, we still lack experimental evidence of exogenous viral processing. This knowledge gap is probably attributable, in part at least, to the challenges associated with isolating and culturing non-model animals and their natural viral pathogens in the lab. Here we seek to examine the phylogenetic distribution of viRNAs, and thus elucidate the phylogenetic distribution of a canonical (i.e. *Drosophila-*, nematode- or plant-like) antiviral RNAi response, through metagenomic sequencing. We combine rRNA-depleted RNA sequencing with small-RNA sequencing to detect both viruses and viRNAs in pooled samples of six deeply divergent lineages. This metagenomic approach circumvents the need to isolate and/or culture non-model organisms in the laboratory, and can capitalise on the high diversity of viruses naturally infecting individuals in the wild. It also avoids any artefactual outcomes that might result from non-native host-virus combinations or non-natural infection routes. First, we include two early branching animal species: a sponge (*Halichondria panicea*: Porifera, Demospongiae) and a sea anemone (*Actinia equina*: Cnidaria, Anthozoa) that branch basally to the divergence between deuterostomes and protostomes (Fig 1). Second, a starfish (*Asterias rubens*: Echinodermata, Asteroidea) that branches basally to vertebrates within the Deuterostomia. Third, two divergent species of Lophotrochozoa—the clade which forms the sister group to Ecdysozoa within the protostomes: a dog whelk (*Nucella lapillus*: Mollusca, Gastropoda) and earthworms (Annelida, Oligochaeta). Finally, to explore the deep history of antiviral RNAi within the eukaryotes, we included the brown alga *Fucus serratus* (Phaeophyceae, Heterokonta), which represents an origin of multicellularity separate from those of plants, fungi, and animals.

We detect 21nt 5’U small RNAs derived from both strands of an RNA virus in the brown alga, similar to virus-derived small RNAs seen in plants and fungi, despite the deep divergence among these multicellular eukaryotes. We also detect miRNAs and somatic TE-derived piRNAs in all the animal lineages, demonstrating that our small-RNA sequencing was successful, and suggesting that somatic piRNAs represent the ancestral state in Bilateria. However, although we find RNA viruses to be common and sometimes highly abundant, we do not find abundant viRNAs in most of the sampled animals. Specifically, we detect no viRNAs from RNA viruses infecting the earthworms, the sponge, or the sea anemone, suggesting that insect- or nematode-like antiviral RNAi is absent from these lineages, and thus potentially their common ancestor. In contrast, we do detect viRNAs from RNA viruses in a gastropod mollusc, the dog whelk. But, unlike the viRNAs of nematodes and arthropods, these peak broadly at 26-30nt, as would be expected of piRNAs—but they lack the 5'U or ‘ping-pong’ signature Finally, we identify primary piRNA-like 26-30nt 5'U small-RNAs derived from putatively endogenous copies of viruses in the sponge, the starfish, and the dog whelk, consistent with a piRNA-like response targeting endogenous virus copies. Together with the known differences among the antiviral RNAi response of plants, fungi, nematodes and arthropods, these findings imply that the true diversity of defensive RNAi strategies employed by eukaryotes may have been underestimated. They also suggest that antiviral RNAi may either be lacking from many animal phyla, or perhaps resembles the antiviral RNAi response reported for mammals.

## Results

### New virus-like sequences identified by metagenomic sequencing

Using the Illumina platform, we generated strand-specific 150 nt paired-end sequence reads from ribosome-depleted RNA extracted from metagenomic pools of each of six different multicellular eukaryotes: the breadcrumb sponge (*Halichondria panacea*, Porifera); the beadlet sea anemone (*Actinia equina*, Cnidaria); the common starfish (*Asterias rubens*, Echinodermata); the dog whelk (*Nucella lapillus*, Mollusca); mixed earthworm species (*Amynthas* and *Lumbricus* spp., Annelida), and a brown alga (the ‘serrated wrack’, *Fucus serratus*, Fucales, Phaeophyceae, Heterokonta). See S1 Table for collection data. Gut contents were excluded by dissection, and contaminating nematodes excluded by a PCR screen prior to pooling (Materials and methods; S1 Table). Reads were assembled separately for each species using Trinity v2.2.0 [95,96], resulting in between 104,000 contigs for the sponge and 235,000 contigs for the earthworms. Metagenomic analysis using Diamond v0.7.11.60 [97] and MEGAN6 [98] suggests the vast majority of these contigs derive for the intended host organism (S1 Fig). For each of the six species pools, the raw meta-transcriptomic contigs generated by Trinity are provided in compressed (gzipped) fasta format as unannotated contigs at https://doi.org/10.6084/m9.figshare.6803885.v1. To identify viruses, we used Diamond to search with translated open reading frames (ORFs) from our contigs against all virus proteins from the NCBI nr database, all predicted proteins from Repbase [99], and all proteins from the NCBI RefSeq_protein database (see Materials and methods). After excluding some low-quality matches to large DNA viruses and matches to phage, this identified nearly 900 potentially virus-like contigs (S1 Data). These matches were examined and manually curated to generate 85 high-confidence virus-like contigs between 0.5 and 12kbp (mean 3.7Kbp) that are the focus of this study. We have provided provisional names for these viruses following the model of Shi *et al*., [100] and the sequences have been submitted to GenBank under accession numbers MF189971-MF190055.

The majority of these virus-like contigs were related to positive sense RNA viruses (+ssRNA), including *ca*. 20 contigs from the Picornavirales, 10 Weivirus contigs, and around 5 contigs each from Hepeviruses, Nodaviruses, Sobemoviruses, and Tombusviruses. We also identified 18 putative dsRNA virus contigs (Narnaviruses, Partitiviruses and a Picobirnavirus) and 11 negative sense RNA virus (-ssRNA) contigs (5 bunya-like virus contigs, 3 chuvirus-like contigs, and two contigs each from Rhabdoviridae and Orthomyxoviridae). Our curated viruses included five DNA virus-like contigs, all of which were related to the single-stranded DNA Parvoviridae. Sequences very similar to our Caledonia Starfish parvo-like viruses 1, 2 and 3 are detectable in the publicly-available transcriptomes of *Asterias* starfish species (101; S2 Fig). Although some of the virus-like contigs are likely to be near-complete genomes, including several +ssRNA viruses represented by single contigs of >9kbp, many are partial genomes representing only the RNA polymerase, which tends to be highly conserved [102]. We identified virus-like contigs from all of the sampled taxa, although numbers varied from only three in the earthworm pool to around 40 in the sponge. This may represent differences in host species biology, but more likely reflects the different range of tissues sampled [103], and/or differences in sampling effort (S1 Text). A detailed description of each putative virus is provided in S2 Table.

**Fig 2:**
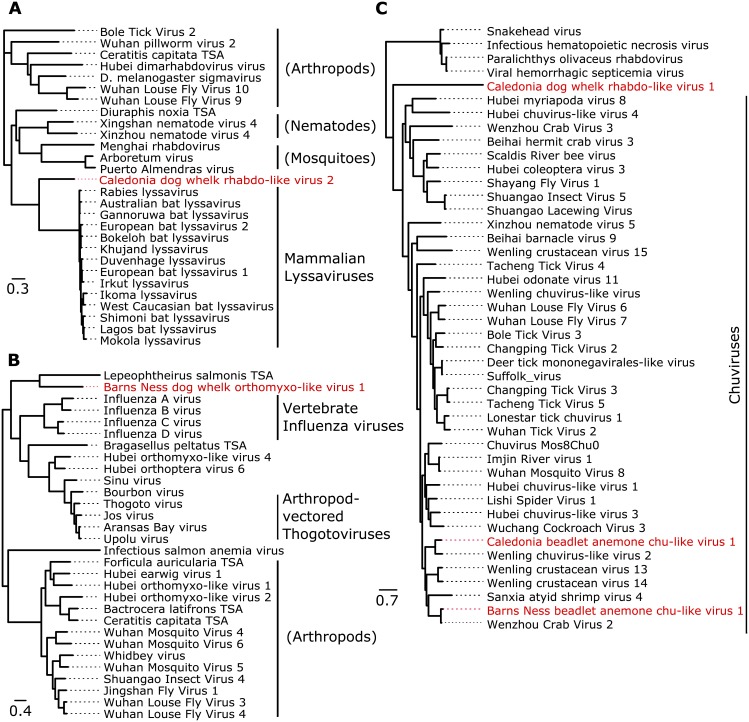
Phylogenetic relationships of virus-like contigs from the dog whelk. Mid-point rooted maximum likelihood phylogenetic trees for each of the virus-like contigs associated with viRNAs in the dog whelk (*Nucella lapillus*). New virus-like contigs described here are marked in red, sequences marked ‘TSA’ are derived from public transcriptome assemblies of the species named, and the scale is given in amino acid substitutions per site. Panels are: (A) rhabdoviruses related to lyssaviruses, inferred using the protein sequence of the nucleoprotein (the only open reading frame available from this contig, which is likely an EVE); (B) orthomyxoviruses related to influenza and thogoto viruses, inferred using the protein sequence of PB1; (C) rhabdoviruses and chuviruses, inferred from the RNA polymerase. Support values and accession identifiers are presented in S2 Fig and S3 Data, and alignments in S2 Data. Given the high level of divergence, alignments and inferred trees should be treated as tentative.

After initially assigning viruses to potential taxonomic groups based on BLASTp similarity, we applied a maximum likelihood approach to protein sequences to infer their phylogenetic relationships. Many of the viruses derived from large poorly-studied clades recently identified by metagenomic sequencing [100,104], and most are related to viruses from other invertebrates. For example, five of the sponge picornavirales were distributed across the ‘Aquatic picorna-like viruses’ clade of Shi *et al*., [100] with closest known relatives that infect marine Lophotrochozoa and Crustacea. Associated with the breadcrumb sponge we identified sequences related to the recently described ‘Weivirus’ clade known from marine molluscs [100], and from the beadlet anemone we identified sequences related to chuviruses of arthropods [100,104]. Some of the virus-like sequences were closely-related to well-studied viruses, for example Millport beadlet anemone dicistro-like virus 1 and Caledonia beadlet anemone dicistro-like virus 2 are both very closely related to Drosophila C virus [105,106] and Cricket Paralysis virus [107]. Others are notable because they lack very close relatives, or because they fall closest to lineages not previously known to infect invertebrates. These include the Caledonia dog whelk rhabdo-like virus 2 sequence, which is represented by a nucleoprotein that falls between the Rabies/Lyssaviruses and other rhabdoviruses, and Barns Ness dog whelk orthomyxo-like virus 1—for which the PB2 polymerase subunit falls between Infectious Salmon Anaemia virus and the Influenza/Thogoto virus clade (Fig 2; the PA polymerase subunit shows similarity to the Thogoto viruses, but not other Orthomyxoviruses). Phylogenetic trees are presented with support values and GenBank sequence identifiers in S2 Fig, and the alignments used for phylogenetic inference and newick-format trees with support values are provided in S2 and S3 Data respectively.

### Evidence supporting the viruses as bone fide infectious agents of the target hosts

In addition to avoiding gut content and/or nematode contamination, we sought to provide four lines of corroborating evidence that these virus-like sequences represent infections of the targeted hosts. First, we estimated the representation of potential hosts in each pool by mapping RNA-seq forward reads to the contigs of Cytochrome Oxidase 1 (COI, a highly expressed eukaryotic gene) that could be identified in our assemblies. COI reads that could not be matched to the target host animals amounted to less than 0.2% of the target’s own COI reads in every case, arguing against substantial contamination with non-target taxa such as parasites or commensals. Contamination was higher in the brown alga, perhaps reflecting the challenge of recovering RNA from this taxon (S1 Text). In this case we identified around 10 contaminating taxa, amounting to 5% of the COI reads, including taxa that we might expect to live as ectocommensals on seaweeds, such as a bryozoan with 3.6% and a tunicate with 1.2%. We also identified some cross-contamination and/or adapter-switching between libraries that shared an Illumina lane [108,109], with a mean of < 0.2% of COI reads deriving from the other libraries in the lane. Nevertheless, an average of 99.78% of the mapped COI reads in each invertebrate library derived from the targeted species (93% in the brown alga), suggesting that any viruses of contaminating species would need to be at a very high copy-number to be detected and erroneously attributed to the target host (read counts are provided in S3 Table).

Second, we remapped reads to the 85 focal virus contigs to measure the number of virus-derived reads relative to host COI. We reasoned that sequence reads from genuine infections are likely to appear in a single host species and to have high representation, whereas viruses present only as surface or sea-water contaminants would be present at low copy-number and seen in association with the multiple hosts that were collected together. We only identified one virus present at an appreciable copy-number in more than one host pool, suggesting that our virus-like sequences do not in general represent biological or experimental contaminants, and that the majority of viruses infected only one of the sampled host species. The exception was a 1.3 kbp partiti-like virus contig (Caledonia partiti-like virus 1), which displayed substantial numbers of reads in both the anemone and the sponge—perhaps indicative of closely related viruses infecting these highly divergent taxa. Four viruses were present at a very high level (>1% of COI in at least one library), including Caledonia beadlet anemone dicistro-like virus 2, Millport beadlet anemone dicistro-like virus, Lothian earthworm picorna-like virus 1, and in the brown alga, Barns Ness serrated wrack bunya/phlebo-like virus 1. In total, 18 of the 85 virus contigs were present at >0.1% of host COI in at least one library, and all but 8 were present at >0.01% of COI (S3 Table, S3 Fig).

Third, we recorded which strand each RNA sequencing read derived from, as actively replicating DNA viruses and -ssRNA and dsRNA viruses generate substantial numbers of positive sense RNAs (S4 Fig). As expected, all of the -ssRNA viruses in our sample (Orthomyxoviridae, Rhabdoviridae, Bunyaviridae/Arenaviridae-like, chuvirus-like) displayed substantial numbers of reads from both strands, consistent with active replication. We also detected negative-sense reads for many of the +ssRNA viruses, but not always at a substantially higher rate than seen for host mRNAs such as COI (S3 Table, S4 Fig. Nevertheless, although +ssRNA viruses also produce complementary (negative sense) RNA during replication, the positive to negative strand ratio is usually very high (e.g. 50:1 to 1000:1 in Drosophila C Virus), potentially making the negative strand hard to detect by metagenomic sequencing. These data provide strong evidence that all of the -ssRNA and dsRNA viruses we detected comprise active infections and are consistent with replication by the other viruses. Surprisingly, only one of the five DNA viruses (Millport starfish parvo-like virus 1) showed the strong positive sense bias expected of mRNAs, whereas the others displayed a negative sense bias. This suggests that these parvovirus-like sequences derived from expressed Endogenous Viral Elements (EVEs; [110]) rather than active viral infections.

Fourth, we selected 53 of the putative virus contigs for further verification by PCR (Materials and methods; S2 Table). For most of these, we confirmed that the template was detectable by RT-PCR but not by (RT-negative) PCR, confirming that the viruses were not present in DNA form, i.e. were not EVEs (Materials and methods; S2 Table). The exceptions were Caledonia dog whelk rhabdo-like virus 2 and (as expected) the DNA parvovirus-like contigs, which did appear in RT-negative PCR. We then estimated virus prevalence in the wild, using RT-PCR to survey all of our samples in pools of between 7 and 30 individuals. The majority of viruses had an estimated prevalence in the range 0.79–100% (S4 Table), with some virus-like sequences present in all sub-pools of the species. These ‘ubiquitous’ sequences included Caledonia dog whelk rhabdo-like virus 2, Caledonia starfish parvo-like virus 2, Caledonia starfish parvo-like virus 3, Caledonia beadlet anemone parvo-like virus 1, and thirteen of the sponge viruses. This suggests that these sequences are common or that they are ‘fixed’ in the population, which could be consistent with integration into the host genome (i.e. an EVE). However, given the sampling scheme, a sponge virus at >36% prevalence has a >95% chance of being indistinguishable from ubiquitous. In addition, with the exception of Caledonia dog whelk rhabdo-like virus 2, none of the RNA viruses could be amplified from a DNA template. Taken together, the use of tissue dissection in RNA preparation, the distribution of viruses across sequencing pools, the host distribution of related viruses, the abundance and strand specificity of virus reads, the absence of DNA copies (for all but one of the RNA viruses), and the variable prevalence in wild populations, support the majority of these sequences as *bone fide* active viral infections of the sampled species.

### Virus and TE-derived 21nt 5'U RNAs are present in a brown alga

**Fig 3:**
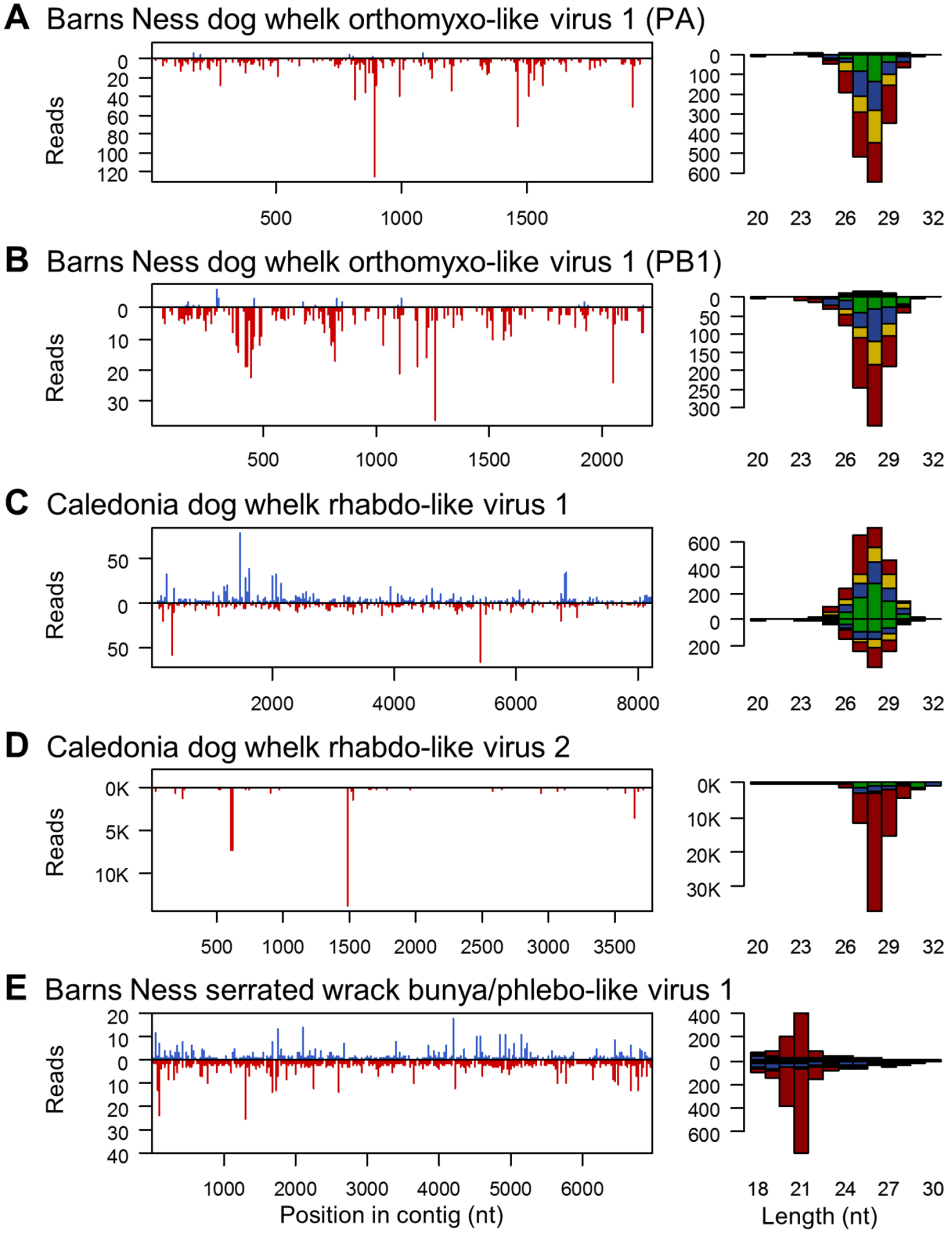
Small RNAs from RNA virus-like contigs. Panels to the left show the distribution of 20-30nt small RNAs along the length of the virus-like contig, and panels to the right show the size distribution small RNA reads coloured by the 5' base (U red, G yellow, C blue, A green). Read counts above the x-axis represent reads mapping to the positive sense (coding) sequence and counts below the x-axis represent reads mapping to the complementary sequence. For the dog whelk (A-D), only reads from the oxidised library are shown. Other dog whelk libraries display similar distributions and the small-RNA ‘hotspot’ pattern along the contig is highly repeatable (S6 Fig). Small RNAs from the two segments of the orthomyxovirus (A and B) show strong strand bias to the negative strand and no 5' base composition bias. Those from the first rhabdo-like virus (C) display little strand bias and no base composition bias, and those from the second rhabdo virus-like contig, which is a probable EVE (D), derive only from the negative strand and display a very strong 5' U bias. There were insufficient reads from the positive strand of this virus to detect a ping-pong signature. Small RNAs from the four dog whelk contigs all display 28nt peaks. Small RNAs from the bunya/phlebo-like virus identified in the brown alga (E) derive from both strands, and show a strong 5' U bias with a peak size of 21nt. The data required to plot the size distributions are provided in S5 Table.

Virus and TE-derived small RNAs have been well characterised in plants, fungi, and some animals, but other major eukaryotic lineages such as Heterokonta, Alveolata, Excavata and Amoebozoa have received less attention. In principle, a metagenomic approach could also be applied to these lineages, but the difficulty of collecting large numbers of individuals of a single lineage makes this challenging for single-celled organisms. Here we have taken advantage of multicellularity in the brown algae (Phaeophyceae, Heterokonta) to test for the presence of viRNAs using the serrated wrack, *Fucus serratus*. Based on a single pooled sample of tissue from 100 individuals, we identified large numbers of small RNAs with a tight distribution between 19 and 23nt, peaking sharply at 21nt (S5 Fig). Almost all of the 21nt sRNAs were 5' U, as has been seen for sRNAs in diatoms (Bacillariophyceae, Heterokonta; [111]) and is seen for some small RNA classes in green plants [112,113] and fungi [114,115]. Although miRNAs have been described for two other brown algae, *Ectocarpus siliculosus* [116,117] and *Saccharina japonica* [118], we were unable to identify homologues of known miRbase miRNAs among these reads. This may reflect a lack of sensitivity, as the miRNA complements of the studied brown algae are highly divergent [118], and miRNAs of *Fucus serratus* may be sufficiently divergent again to be undetectable based on sequence similarity. In contrast, 1.8% of small RNAs corresponded to the subset of high-confidence TE contigs. These small RNAs were derived from both strands, but as expected given the absence of Piwi, displayed no evidence of ‘ping-pong’ amplification—with sRNAs from both strands showing a 5' U bias. Most interestingly, we also detected viRNAs corresponding to a -ssRNA bunya-like virus (Barns Ness serrated wrack bunya/phelobo-like virus 1; Fig 3E, S6 Fig). Although numbers were relatively small, comprising 0.01% of all small RNA reads, these derived from both strands along the full length of the virus-like contig, peaked sharply at 21nt, and were almost exclusively 5'U. We did not detect a viRNA signature from a further two -ssRNA or from four dsRNA virus-like contigs, although their copy-number was very low compared to Barns Ness serrated wrack bunya/phelobo-like virus 1 (S3 Fig, S3 Table).

### Virus-derived small RNAs are detectable in a dog whelk, but not other animal samples

Based on our knowledge of antiviral RNA interference in arthropods and nematodes, we expected viral infections in our animal samples to be associated with large numbers of Dicer-generated viRNAs, with a narrow size distribution peaking between 20nt (as seen in Lepidoptera; [119]) and 22nt (as seen in chelicerates, nematodes, and hymenopterans; [42,120,121]). Because animal piRNAs and viRNAs are generally modified by the addition of a 3' 2-O-methyl group, and some nematode small RNAs are generated by direct syntheses (resulting in a 5’ triphosphate group) our sequencing included small RNAs treated with 5' polyphosphatase (to remove 5’ triphosphates) and oxidised RNA (to increase the representation of small RNAs bearing a 3' 2-O-methyl group). Furthermore, to ensure that we did not exclude viRNAs that had been edited (e.g. by ADAR; [122]), or that contained untemplated bases (e.g. 3' adenylation or uridylation; [123]), our mapping approach permitted at least two high base-quality mismatches within a 21nt sRNA. We also confirmed that remapping with local alignment, which permits any number of contiguous mismatches at either end of the read, did not substantially alter our results.

We successfully recovered abundant miRNAs in all of the animal samples, with between 20% (sponge) and 80% (starfish) of 20-23nt RNAs from untreated libraries mapping to known miRbase miRNAs [124]. Consistent with the absence of a 3' 2-O-methyl group, these miRNA-like reads had much lower representation in the oxidised libraries, there comprising only 0.4% (earthworms) to 14% (dog whelk) of 20-23nt RNAs. We also identified characteristic peaks of small RNAs derived from ribosomal RNA at 12nt and 18nt in the sponge, at 12nt and 16nt in the sea anemone, and in oxidised libraries from all organisms. The only exception to this overall pattern was for the sea anemone, in which oxidation had no effect on the relative number of miRNAs, although did strongly affect the overall size distribution of rRNA-derived sRNAs. This suggests the presence of a 3' 2-O-methyl group in sea anemone miRNAs (S5 Fig). A few small RNAs also mapped to contigs nominally identified as bacterial in origin (S7 Fig) but numbers were small in the Sea Anemone, Dog Whelk, and Starfish, while those the brown alga were predominantly degradation products from bacterial rRNA, and manual inspection suggests the vast majority in the sponge and earthworm derived from miss-classified TE sequences.

Surprisingly, despite our identification of more than 40 RNA virus-like contigs associated with the sponge, 17 in the sea anemone, and three in the earthworms, we were unable to detect a signature of abundant viRNAs in any of these three organisms. On average, less than 0.002% of 17-35nt RNAs from these organisms mapped to the RNA virus contigs, and those that did map were enriched for shorter lengths (17-19nt), lacked a clearly defined size distribution, and were less common in the oxidised than non-oxidised libraries (S5 Fig, S3 Table)—features consistent with non-specific degradation products, rather than viRNAs. (Note that the starfish sample lacked detectable RNA viruses, precluding the identification of RNA-virus viRNAs).

The only metazoan sample to display a viRNA signature was the dog whelk (*Nucella lapillus*), with 0.14% of oxidised small RNAs derived from four of the seven RNA virus-like contigs. These included both contigs of Barns Ness dog whelk orthomyxo-like virus 1, Caledonia dog whelk rhabdo-like virus 1, and Caledonia dog whelk rhabdo-like virus 2. A Narnavirus-like contig and a very low copy-number Bunyavirus-like contig were not major sources of viRNAs. Given the absence of detectable viRNAs in the Sponge, Sea Anemone, and Earthworm, it is notable that the viRNA-producing viruses in the dog whelk were present at a much lower copy number than many viRNA-free viruses in those organisms (e.g. Lothians earthworm picorna-like virus 1, Barns Ness breadcrumb sponge hepe-like virus 1; S3 Fig). This suggests that, had viRNAs been present in those taxa, we were likely (for many viruses) to have been be able to detect them.

Nevertheless, the virus-derived small RNAs seen in the dog whelk did not show the expected size, strand, or 2nt overhang signature of canonical Dicer-generated viRNAs (Fig 3, S6 Fig). Instead, viRNA lengths formed a broad distribution from 26 to 30nt (peaking at 28nt), more consistent with piRNAs seen in the *Drosophila* and mammalian germlines. These small RNAs were derived almost entirely from the negative-sense (i.e. genomic) strand of Barns Ness dog whelk orthomyxo-like virus 1 (Fig 3A and 3B) and Caledonia dog whelk rhabdo-like virus 2 (Fig 3D), but from both stands of Caledonia dog whelk rhabdo-like virus 2 (Fig 3C and 3E). Although this size distribution is more consistent with the piRNA pathway, only those from Caledonia dog whelk rhabdo-like virus 2 (a suspected EVE, see above) displayed the strong 5'U bias expected of primary piRNAs (Fig 3D), and none showed any evidence of ping-pong amplification. In all three cases, the putative dog whelk viRNAs were derived from the whole length of the viral genome—albeit with strong hotspots in Caledonia dog whelk rhabdo-like virus 2. None of these findings were qualitatively altered by a requirement for perfect (zero mismatch) mappings, or by permitting local mapping. Relative to miRNAs, these RNA-virus derived viRNAs were much more strongly represented in the oxidised library than the untreated library, with the miRNA:viRNA ratio increasing 300-fold—consistent with the presence of a 3' 2-O-methyl group (S5 and S6 Figs).

### The sea anemone and starfish display 5'U 26-30nt RNAs from DNA virus-like contigs

**Fig 4:**
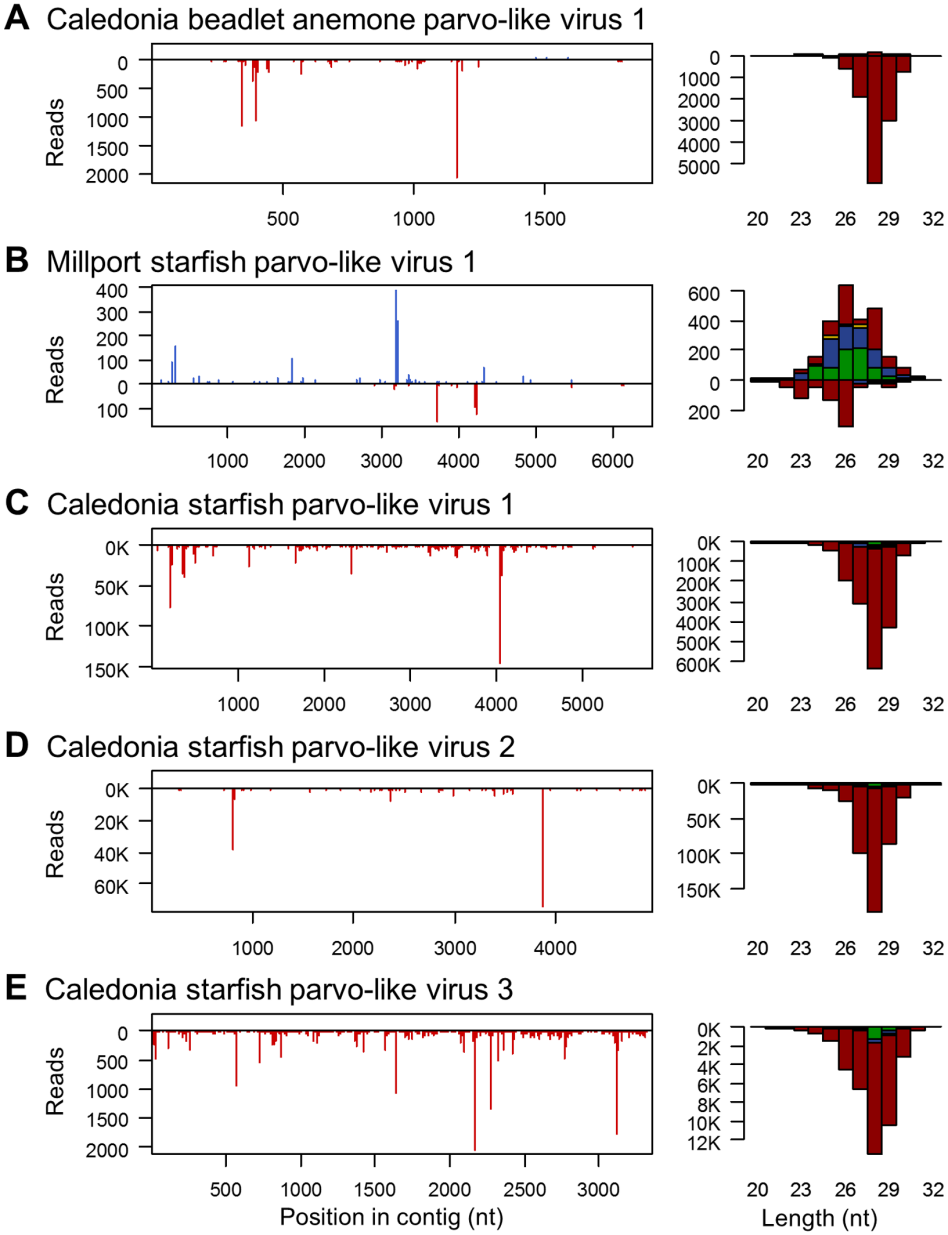
Small RNAs from DNA parvo/densovirus-like contigs. Panels to the left show the distribution of 20-30nt small RNAs along the length of the parvo/densovirus-like contigs from sea anemone (A) and starfish (B-E), and panels to the right show the size distribution small RNA reads coloured by the 5' base (U red, G yellow, C blue, A green). Read counts above the x-axis represent reads mapping to the positive sense (coding) sequence, and counts below the x-axis represent reads mapping to the complementary sequence. Only reads from the oxidised library are shown, but other libraries display similar distributions, and the small-RNA ‘hotspot’ pattern is highly repeatable (S6 Fig). For all but one of the parvo/denso-like virus contigs, the small RNAs derived exclusively from the negative sense strand and showed a strong 5'U bias, consistent with piRNAs derived from endogenous copies (see main text). For one contig (B: Millport starfish parvo-like virus 1) reads derived predominantly from the positive strand and did not display a 5' U bias. Although the number of unique small RNA sequences from this virus was small, the positive-sense small RNAs showed a slight bias to A at position 10, consistent with ping-pong (S6 Fig). The data required to plot these size distributions is provided in S5 Table.

DNA viruses are a source of Dicer-mediated viRNAs in arthropods and in plants, and antiviral RNAi pathways are important for antiviral immunity to DNA viruses in both groups (reviewed in [125,126]). Although our RNA sequencing strategy was intended to detect RNA viruses, we also identified four novel parvo/densovirus-like contigs (Parvoviridae; single-stranded DNA) in the starfish, and one in the sea anemone. These sequences constituted a substantial source of small RNAs in both organisms, particularly the starfish—contributing 0.3% of small RNAs in the untreated libraries and 3.4% of small RNAs in the oxidised library. In four of the five cases these small RNAs were almost exclusively negative sense, were 26 to 30nt in length (peaking at 28nt), and were very strongly biased toward U in the 5' position—resembling primary piRNAs (Fig 4). However, the high prevalence and/or negative strand RNAseq bias (S4 Fig) of these source contigs is consistent with expressed genomic integrations (EVEs) rather than active viral infections. In the case of Millport starfish parvo-like virus 1, both positive and negative sense reads were detectable, the negative sense reads again displayed a strong 5' U bias, but the positive sense reads displayed a postion-10 ‘A’ ping-pong signature (Fig 4B), as expected of piRNAs. Relative to miRNAs, these putative piRNAs were much more strongly represented in the oxidised library than the untreated library, consistent with the presence of a 3' 2-O-methyl group (S5 and S6 Figs).

### All of the sampled animals display somatic TE-derived piRNAs

**Fig 5:**
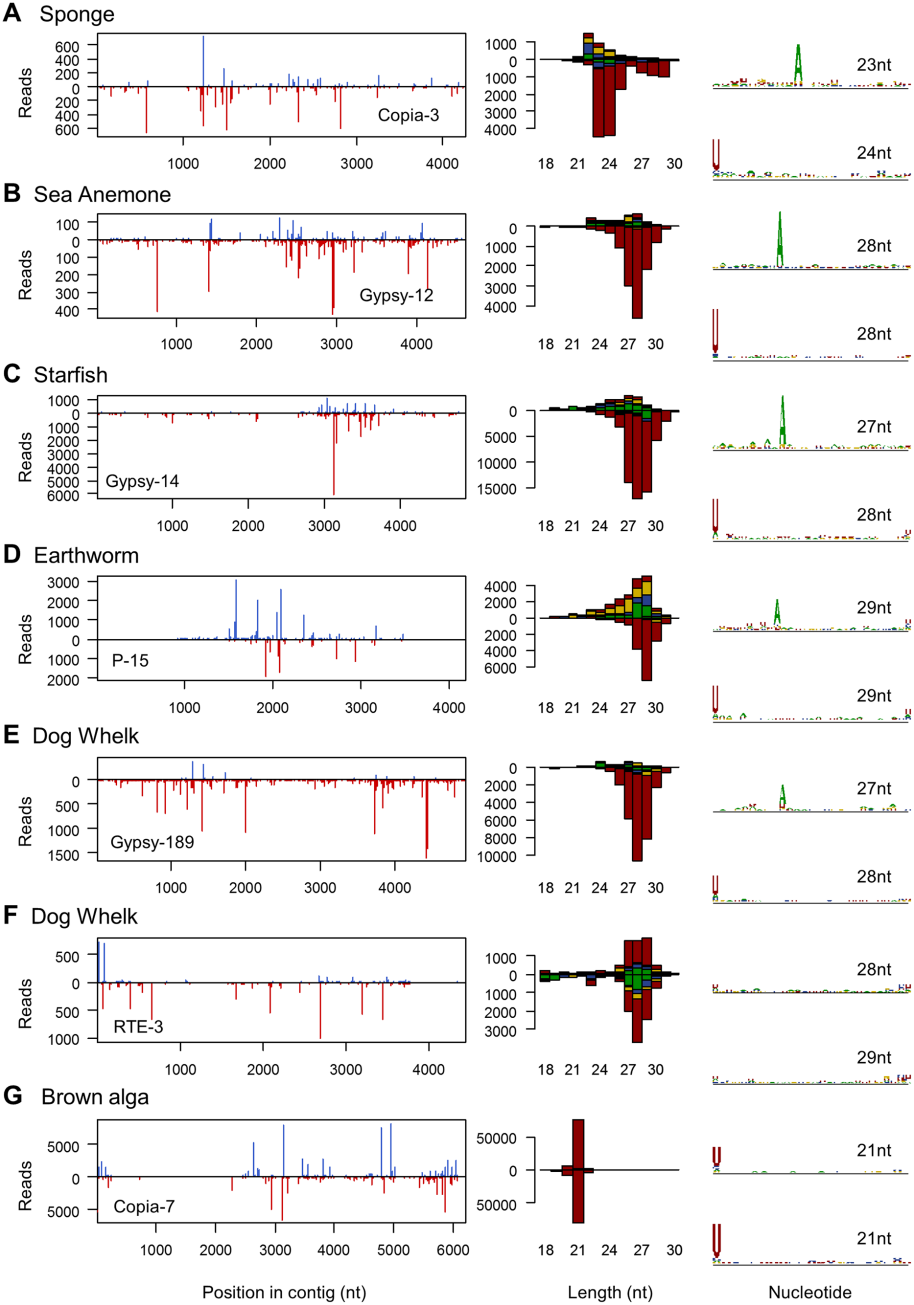
Small RNAs from TE-like contigs. The threecolumns show (left to right): the distribution of 20-30nt small RNAs along the length of a TE-like contig; the size distribution of small RNA reads (U red, G yellow, C blue, A green); and the sequence ‘logo’ of unique sequences for the dominant sequence length. Read counts above the x-axis represent reads mapping to the positive sense (coding) sequence, and counts below the x-axis represent reads mapping to the complementary sequence. For the sequence logos, the upper and lower plots show positive and negative sense reads respectively, and the y-axis of each measures relative information content in bits. Where available, reads from the oxidised library are shown (A-F), but other libraries display similar distributions (S8 Fig). These examples from sponge (A), sea anemone (B), starfish (C), earthworm (D), dog whelk (E-F) and brown alga (G) were chosen to best illustrate the presence of the ‘ping pong’ signature, but other examples are shown in S8 Fig. Note that the size distribution of TE-derived small RNAs varies substantially among species, and that the dog whelk (E and F) displays at least two distinct patterns, one (F) reminiscent of that seen for some RNA virus contigs (Fig 3C). The data required to plot these figures is provided in S5 Table.

Transposable elements and TE-derived transcripts represent a major source of piRNAs in the germlines of *Drosophila* [76], *C*. *elegans* [127,128], mice [129,130], and zebrafish [75], although the germline limitation seen in *Drosophila* is derived within the Arthropods [17]. Piwi-interacting RNAs are also detectable in Cnidaria and Porifera, although their tissue specificity is unclear [15]. Furthermore, TE transcripts in *Drosophila* and some other arthropods are also processed by Dicer to generate 21nt endo-siRNAs [17]. We therefore selected a total of 146 long, high-confidence, TE contigs from our assemblies to analyse TE-derived small RNAs (these contigs were selected on the basis of length and similarity to repBase entries, and to best illustrate small RNA properties; contigs are provided in S4 Data). We identified large numbers of TE-derived putative piRNAs in the somatic tissues of all the sampled organisms (Fig 5). In total, between 0.17% (starfish) and 1.7% (dog whelk) of untreated small RNA reads mapped to the 146 high-confidence TE contigs (S2 Data, S5 and S8 Figs). In every case except the anemone, the putative piRNAs were more highly represented in the oxidised library than in untreated or polyphosphatase-treated libraries (1.4–6% of oxidised reads), suggesting that they are 3' 2-O-methylated and result from cleavage rather than synthesis. Despite very large numbers of piRNAs for some TE contigs, we did not observe endo-siRNA -like small RNAs similar those observed in *Drosophila* and some other arthropods (e.g. [17,131]).

We observed putative piRNAs derived from one or both strands of the TEs (Fig 5). Where they derived predominantly from a single strand they were generally strongly 5'U-biased (consistent with primary piRNAs). Where they derived from both strands, those from the second strand presented evidence of ‘ping pong’ amplification (i.e. no 5' U bias, and a strong ‘A’ bias at position ten; Fig 5, S8 Fig). However, the piRNA size distribution varied substantially among organisms and TEs. In the sponge, the length of the 5' U-biased piRNAs either peaked at 23-24nt, or presented a broader bimodal distribution peaking at 23-24nt and 27-29nt. Where piRNAs derived from both strands, the strand with a ping-pong signature showed a shorter length distribution (22-23nt). In a few cases the putative sponge piRNAs from both strands showed a strong 5'U bias with no evidence of ping-pong amplification. In the sea anemone we consistently identified a strong peak of 5'-U biased sRNAs peaking at 28-29nt on one strand, but a generally bimodal distribution from the second ‘ping-pong’ strand (if piRNAs were present), peaking at around 23nt and 28nt. Again, both strands occasionally displayed a 5'-U bias and no evidence of ping-pong amplification. The patterns were again similar in the starfish and the earthworms, except that size distributions were unimodal, peaking at 29-30nt in the 5'-U biased strand and 25-26nt (starfish) and 26-27nt (earthworms) in the ‘ping-pong’ strand.

As with viRNAs, the only exception to this general pattern was seen in the dog whelk. In addition to TE-like contigs that displayed a classical piRNA-like signature (28nt 5'U RNAs from one strand; 26-28nt ‘ping-pong’ RNAs from the opposite strand), a small number of TE-like contigs in the dog whelk had an sRNA signature that resembled that of Barns Ness dog whelk orthomyxo-like virus 1 and Caledonia dog whelk rhabdo-like virus 1. In these TE-like contigs, the sRNAs were derived from one or both strands, peaked broadly at 26-30nt, and lacked any bias in base composition or evidence of ‘ping-pong’ (Fig 5E and 5F). This indicates that some TEs are processed in the same way as the identified RNA viruses, (e.g. Gypsy; S8D Fig). A minority of TE-like contigs displayed an intermediate pattern, with a weak 5'U-bias from one strand, and a broad peak that lacked a pong-pong signature from the other strand. Such an intermediate pattern could result either from a single TE targeted by two different mechanisms, or from cross-mapping of sRNAs derived from different copies of the same TE inserted in different locations/contexts. As before, our permissive mapping approach and re-mapping using local alignments reduces the possibility that a large category of sRNAs escaped detection, and a requirement for zero mismatches had no qualitative impact on our results.

### The phylogenetic distribution and expression of RNAi-pathway genes

We sought to examine whether the phylogenetic distribution and expression of RNAi pathway genes in our samples was consistent with the small RNAs we observed. As expected, based on the presence of abundant miRNAs and/or an antiviral pathway and given what is known for their close relatives [15,132–138], we identified two deeply divergent Dicer transcripts in the sea anemone, and a single Dicer transcript in each of the other animal species. The single Dicers seen in the starfish, dog whelk, and earthworms were more similar to Dicer-1 from the *Drosophila* miRNA pathway than to arthropod Dicer-2-like genes that mediate antiviral RNAi. Similarly consistent with an antiviral RNAi and/or a miRNA pathway, and with what is known for their close relatives [42,135,136,139–143], we identified two deeply divergent (non-Piwi) Argonaute transcripts in the sponge and in the anemone (S6 Table), and single Argonaute transcripts in the dog whelk and in the starfish. We identified three distinct Argonaute transcripts in the mixed-earthworm species pool, although these may represent the multiple earthworm species present. The dog whelk, starfish, and earthworm Argonautes were all more closely related to arthropod Ago-1 (which binds miRNAs but rarely viRNAs) and to vertebrate Argonautes, than to insect Ago2-like genes that mediate antiviral RNAi. It is likely that these genes mediate the miRNA pathway in these organisms, although it is possible that they may also mediate the production of novel viRNAs seen in the dog whelk. We also identified a single Dicer and Argonaute in the *Fucus*, which is consistent with what has been seen in other brown algae [116–118], and with the presence of both miRNAs and viRNAs.

Host-encoded RNA-dependent RNA polymerases (RdRp) play a key role in antiviral RNAi responses in plants [144] and nematodes [145,146], underlining the substantial diversity in antiviral RNAi pathways across eukaryotes. However, their role in RNAi in other animals is unknown, and they have an extremely patchy distribution across the animal phylogeny with multiple independent losses. For example, they are absent from Vertebrata and Pancrustacea, but are present in Porifera, Cnidaria, Chelicerata, Nematoda, Bivalvia, Brachiopoda, some Platyhelminthes, and non-vertebrate Deuterostomia. We identified three host RdRps in the Sea Anemone, each closely related to sequences from *Exaiptasia pallida*. We also identified a single RdRp sequence in the sponge and three in the Earthworm, although these did not cluster with their closest sequenced relatives. We were unable to identify any RdRp sequences in the dog whelk or the starfish, or in the brown alga, but it remains possible that they are present and expressed at a level too low for us to detect.

In animals, the piRNA pathway suppresses transposable element transcripts, and is mediated by homologs of the *Drosophila* nuclease ‘Zucchini’ and the Piwi-family Argonaute proteins Ago3 and Piwi/Aub. In mammals, fish, *C*. *elegans* and *Drosophila*, this pathway is primarily active in the germline and its associated somatic tissues [75,76,127–130], whereas in sponges and cnidarians—which lack a segregated germline—and many other arthropods, Piwi homologs are ubiquitously expressed [17,71,147]. Consistent with our finding of TE-derived piRNAs displaying a canonical ‘ping-pong’ signature, we identified single Zucchini, Ago3 and Piwi homologs in four of the five animals surveyed (S6 Table). The exception was the sea anemone, in which we could only identify a single Piwi (more similar to *Drosophila* Piwi/Aub than to Ago-3). Surprisingly, although we did not identify canonical piRNAs in the brown alga, we did identify a possible Piwi-like transcript. However, its relatively low expression and apparent similarity to Piwi genes from the Lophotrochozoa suggest it most likely derives from the contaminating bryozoan identified by COI reads (above). Finally, consistent with the altered small RNA profile associated with oxidation, we were able to identify a single homolog of the RNA methyl transferase Hen-1 in each of the animal species, but not in the brown alga. These sequences have been submitted to GenBank under accession numbers MF288049-MF288076.

## Discussion

### Evidence for antiviral RNAi against -ssRNA viruses in the dog whelk and brown alga

Antiviral RNAi is an important defence mechanism in plants and many fungi, and in nematodes and arthropods, where it generates large numbers of easily detectable virus-derived small RNAs in wild-type individuals. Here we identified abundant viRNAs from RNA viruses in two of the six multicellular Eukaryotes we tested: from a bunya/phlebo-like virus in a brown alga (*Fucus serratus*) and from three different RNA virus-like contigs in the dog whelk (*Nucella lapillus*). The viRNAs from the brown alga strongly resembled other classes of small RNA from brown algae [117,118] and viRNAs from fungi [114,115] and some viRNAs from plants [113], consistent with an antiviral RNAi response in this species. The viRNAs from the dog whelk similarly displayed a distinct size distribution, derived from the full length of the viral sequence, and were over-represented after oxidation—implying the presence of a 3' 2-O-methyl group (Fig 3, S6 Fig). However, their broad length distribution around 28nt and the strong strand-bias were not consistent with Dicer processing, which is expected to generate sRNAs from both strands simultaneously and to result in a characteristic sequence length determined by the distance between the PAZ and RNaseIII domains [148]. We therefore suggest that these distinctive viRNA are consistent with an active, but divergent, antiviral RNAi pathway in this species.

We have also considered three alternative explanations for these data. First, it is possible that the result is artefactual, and that all of the virus-like reads derive from another unknown source, such as environmental contamination. However, the large number of complementary (mRNA) sequences show the -ssRNA viruses to be active, the sequences were not identified in any of the other co-collected taxa, and the COI read counts in the dog whelk show contamination rates to be low. Contamination was higher for the brown alga, but the virus would need to be at extremely high copy number in the contaminating taxon to achieve the observed 3% of brown alga COI expression. Second, it is possible that the virus-like contigs represent expressed host loci, such as EVEs. However, sequences were not detectable by PCR in the absence of reverse transcription, and in the dog whelk the low and variable population prevalence means that any putative EVE must be segregating and at very different frequencies in different samples—more consistent with an infectious agent. Moreover, in a previous analysis of insect viruses, expressed EVEs were found to be rare relative to active viral infections: zero of 20 viruses identified by metagenomic sequencing in *Drosophila* [149]. Third, even if the virus-like sequences do represent real infections, it is possible that the small RNAs do not represent an active RNAi-like response. However, their distinctive size distributions and the presence of a 3' 2-O-methyl group in the dog whelk and near 100% 5'U in the brown alga, argue strongly that these viRNAs are the result of active biogenesis rather than degradation.

In contrast, it seems probable that the shorter rhabdo-like virus fragment from the dog whelk (Caledonia dog whelk rhabdo-like virus 2; Fig 3D) is a host-encoded EVE. First, the only open reading frame is homologous to a nucleoprotein and we could not detect a polymerase—despite its close relationship with the nucleoprotein of Lyssaviruses (Fig 2A). Second, RNA sequencing was dominated by negative-sense reads, suggesting a lack of mRNA expression, but consistent with host-driven expression of an integrated locus. Third, the small RNAs were exclusively negative-sense and 5'U, as sometimes seen for primary piRNAs derived from EVEs in other taxa. Fourth, the sequence was ubiquitous in our population samples, consistent with fixation and thus genome integration. Fifth, we were able to PCR amplify a band from a DNA template. If this sequence is an EVE, this could represent an alternative antiviral RNAi mechanism, akin to the piRNA-generating EVEs seen in *Aedes* mosquitoes [150].

### Evidence for substantial variation in antiviral RNAi-like responses to RNA viruses

Despite the presence of more than 70 high-confidence RNA virus-like contigs, we were unable to identify an abundant or distinct population of viRNAs from RNA viruses in the sponge, sea anemone, or earthworm samples (the starfish sample lacked detectable RNA viruses). Whereas the -ssRNA viruses in the dog whelk produced 1–100 viRNA reads per RNAseq read (S9 Fig), and Barns Ness serrated wrack bunya/phelbo-like virus 1 in the brown alga produced *ca*. 0.1 viRNA reads per RNAseq read (S9 Fig), none of the other RNA viruses gave rise to ≥0.001 viRNA reads per RNAseq read. In contrast, in an equivalent analysis of *Drosophila*, all putative viruses produced viRNAs at approximately 10–1000 viRNAs per RNAseq read [149]. This represents a striking difference in the processing of RNA viruses between arthropods [17,38,39,42] and nematodes [44,45,120], and the processing of viruses by sponges (Porifera), anemones (Cnidaria), and earthworms (Annelida). Importantly, it suggests that these animal lineages either do not process RNA viruses into small RNAs in the way that plants, fungi, nematodes or insects do, or that they do so at a level that is undetectable through the bulk small RNA sequencing of wild-type organisms and viruses—as has been reported to be the case for mammals [31,33–35]. The broad distribution of dsRNA-inducible gene knockdown reported across the animals (Fig 1) may support the latter (cryptic small RNAs) explanation. However, it is also possible that these knockdowns function through the Dicer that mediates the miRNA pathway, as it does in *C*. *elegans*. In either case our data imply that the antiviral RNAi mechanisms seen in arthropods and nematodes are highly derived and unlikely to represent the ancestral state in Metazoa.

Nevertheless, it is necessarily hard to demonstrate that RNA viruses do *not* give rise to small RNAs in these lineages: an absence of evidence provides weak evidence of absence. For example, it is possible that small RNAs are abundant, but were not detected. This is highly unlikely as we were able to detect miRNAs, piRNAs, and small rRNAs, and we would also have detected viRNAs bearing a 5' triphosphate or 3' 2-O-methyl group, as well as viRNAs that had been edited or extended by untemplated bases at the 5' or 3' end. One alternative is that all of the RNA-virus like contigs that we identified from the sea anemone, sponge, and earthworm, were inactive and/or encapsidated at the time of collection, and thus not subject to Dicer processing. However, this is unlikely for three reasons. First, it can be ruled out for eight of the nine highest copy-number dsRNA viruses in the sponge, as these all showed a strong positive-strand RNAseq bias, consistent with gene expression. Second, it is not supported by the two -ssRNA virus contigs in the earthworms, which also displayed positive sense mRNA reads (although the virus copy-number was extremely low, such that that we had little power to identify either positive sense RNAseq reads or viRNAs). Finally, although the small number of negative sense reads resulting from +ssRNA virus replication makes it hard to exclude the possibility that they were inactive, it would be surprising if all of the -ssRNA viruses and dsRNA viruses (including those in the dog whelk and brown alga) were active, but none of the +ssRNA viruses were.

Perhaps a more plausible alternative is that the remaining viruses express viral suppressors of RNAi (VSRs) that completely eradicate the small RNA signature, such that it is undetectable through bulk sequencing of wild-type individuals. This appears to be the case for some mammalian viruses, where viruses genetically modified to remove their VSR do indeed form a much greater source of small RNAs [31,32,34,35]. However, it is not the case for the many insect and plant viruses that express well-characterised VSRs [151,152], and while it could certainly be true for some of the 80 different viruses we detect, it would be surprising if it were true for all of them.

It is also possible that abundant viRNAs are characteristic of a response against -ssRNA viruses in anemones, earthworms, and sponges, but are not characteristic of the response against +ssRNA or dsRNA viruses. This could also be consistent with our failure to detect viRNAs from putative dsRNA narnaviruses in the dog whelk and brown alga, and to a putative +ssRNA nodavirus in the brown alga. If so, then an apparent absence of antiviral RNAi in the sponge, sea anemone and earthworms may really reflect differences in the composition of the RNA virus community, with a preponderance of -ssRNA viruses in the dog whelk and their absence from the sponge or anemone. However, even if -ssRNA viruses, but not +ssRNA viruses or dsRNA viruses, give rise to viRNAs in most animal lineages, then this is still in striking contrast to the antiviral RNAi response in plants, fungi, nematodes and insects [9,38,153], and again suggests that antiviral RNAi mechanisms are highly variable among eukaryotic lineages. Finally, it also remains possible that the majority of sponge, sea anemone, and annelid species do possess an active antiviral RNAi mechanism that generates abundant viRNAs from RNA viruses, but that the particular species we examined here have lost the ability. It is certainly the case that RNAi mechanisms are occasionally lost, as in one clade of the yeast genus *Saccharomyces* [23,154]. However, unless antiviral RNAi is lost extremely frequently in these three animal phyla—which is not the case in arthropods or plants—it is extremely unlikely that we would by chance select three lineages that have lost the mechanism while others retained it.

### Evidence for Piwi-pathway targeting of DNA viruses in the sea anemone and starfish

We identified four parvo/denso-like virus contigs in the starfish, and one in the sea anemone. All of these sequences were detected as RNAseq reads and were associated with abundant 26-29nt piRNA-like small RNAs (Fig 4). However, RNAseq from three of the four starfish parvo/denso-like virus contigs, and the sea anemone contig, were dominated by negative sense reads. This is hard to reconcile with the normal functioning of ssDNA parvo/denso-like viruses, and may instead reflect host-driven transcription. For these four contigs, the small RNAs were also almost exclusively negative-sense and 5'U—as expected of primary piRNAs. In contrast, RNAseq and small RNAs reads from Millport starfish parvo-like virus 1 were almost exclusively positive (mRNA) sense, with the negative strand small RNAs showing a 5'U bias and positive strand sRNAs showing weak ‘ping-pong’ signature (S6 Fig). Together, these observations suggest that at least some of parvo/denso-like virus sequences represent expressed EVEs, but also that they are targeted by a piRNA pathway-related mechanism.

Unlike for RNA viruses, we were unable to test whether these sequences represent integrations into the host genome, as integrations are indistinguishable from viral genomic ssDNA by PCR, and both +ssDNA and -ssDNA sequences are usually encapsidated by densoviruses. However, Caledonia starfish parvo-like viruses 1, 2 and 3 are nearly identical to published starfish transcripts, and the two published sequences most similar to Caledonia beadlet anemone parvo-like virus 1 are from an anemone transcriptome and an anemone genome (S2 Fig). In addition, three of the five contigs (two in the starfish, and one in the anemone) appear to be ubiquitous in our wild sample. This ubiquitous distribution and close relationship to published sequences support the suggestion (above) that some of these sequences may be host integrations. The exceptions are Caledonia starfish parvo-like virus 1 and Millport starfish parvo-like virus 1, which both had an estimated prevalence of between 4% and 20% in the larger Millport collection. We were able to recover putatively near-complete genomes of 6.5 and 5.8 Kb, containing the full length structural (VP1) and non-structural (NS1) genes, from Millport starfish parvo-like virus 1 and Caledonia starfish parvo-like virus 1, respectively (S2 Table).

If these sequences are EVEs, as seems very likely for four of the five, then their expression and processing into piRNAs may reflect the location of integration—for example, into or near to a piRNA generating locus [155,156]. In contrast, if these sequences are not host EVEs, then the high expression of negative sense transcripts and the presence of primary piRNA-like small RNAs suggests an active Piwi-pathway response targeting DNA viruses in basally-branching animals. These are not mutually exclusive, and it is tempting to speculate that such integrations could provide an active defence against incoming virus infections in basal animals, as suggested for RNA-virus integrations in *Aedes* mosquitoes [150]. If so, the low-prevalence Millport starfish parvo-like virus 1 sequence, which shared 72% sequence identity with Caledonia starfish parvo-like virus 1, but displayed positive sense transcripts, positive and negative sense piRNAs and a ‘ping-pong’ signature, is a good candidate to represent an unintegrated infectious virus lineage.

### Implications for the evolution of RNAi pathways

The absence of detectable viRNAs in the sponge, sea anemone, or earthworm samples, combined with the presence of 26-29nt (non-piwi) viRNAs in the mollusc and 21nt 5'U viRNAs in the brown alga, reinforces the diversity of antiviral RNAi mechanisms in multicellular eukaryotes. Previously, the abundant viRNAs present in plants, fungi, nematodes and arthropods had implied that Dicer-based antiviral RNAi was ancestral to the eukaryotes and likely to be ancestral in animals, with a recent modification [or even loss; 24] in the vertebrates—perhaps associated with the evolution of interferons [157]. Our findings now suggest three alternative hypotheses. First, antiviral RNAi may have been absent from ancestral animals, and re-evolved on at least one occasion—giving rise to the distinctively different viRNA signatures seen in nematodes, arthropods, vertebrates, and now also a mollusc. Second, the ancestral state may have been more similar to current-day mammals, which do not produce abundant easily-detected viRNAs under natural conditions, but may still possess an antiviral RNAi response [31,33–35]. In this scenario, antiviral RNAi has been maintained as a defence—possibly since the origin of the eukaryotes—but has diversified substantially to give the distinctive viRNA signatures now seen in each lineage. Third, dsRNA, +ssRNA, -ssRNA, and DNA viruses may be targeted differently by RNAi pathways in divergent animal lineages, but arthropods have recently evolved a defence that gives rise to the same viRNA signature from each class. It is not possible to distinguish among these hypotheses without broader taxonomic sampling and experimental work in key lineages. For example, analyses of the Ago-bound viRNAs of Cnidaria and Porifera could help to distinguish between the first two hypotheses, and an identification of the nucleases and Argonautes and/or Piwis required for the 26-29nt mollusc viRNAs could establish whether this response is derived from a Dicer/Ago pathway or a Zucchini/Piwi like pathway. In each case, the limited taxonomic sampling and a lack of experimental data from these non-model taxa preclude any firm conclusions, and given the alternative possibilities outlined above, our interpretations should be treated as tentative. Nevertheless, the balance of evidence strongly suggests that the well-studied canonical antiviral RNAi responses of *Drosophila* and nematodes are likely to be derived compared to the ancestral state, and that there is substantial diversity across the antiviral RNAi mechanisms of multicellular eukaryotes.

The presence of piRNAs derived from transposable elements in the soma of all of the sampled animals also demonstrates a previously under-appreciated diversity of piRNA-like mechanisms. First, it argues strongly that the predominantly germline expression of the piRNA pathway in key model animals (vertebrates, *Drosophila*, and nematodes) is a derived state, and that “ping-pong” mediated TE-suppression in the soma is likely to be common in other animal phyla, as has been shown for arthropods [17], and has recently been confirmed in two other molluscs [158]. Second, it suggests that the TE-derived endo-siRNAs seen in *Drosophila* and mosquitoes [64,155,159–161] are absent from most phyla, and are therefore a relatively recent innovation. Third, the diversity of piRNA profiles we see among organisms—such as the bimodal length distributions of primary piRNAs in the sponge and in “ping-pong’ piRNAs in the sea anemone—suggests substantial variation among animals in the details of piRNA biogenesis. Finally, the large numbers of primary piRNAs derived from putative endogenous copies of parvo/denso-like viruses in the starfish and sea anemone, and from the putatively endogenous rhabdo-like virus 2 in the dog whelk, suggests that the piRNA processing of endogenous virus copies may be widespread across the animals, perhaps even representing an additional ancient defence mechanism.

## Materials and methods

### Sample collections and RNA extraction

We sampled six organisms: The breadcrumb sponge *Halichondria panacea* (Porifera: Demospongiae), the beadlet anenome *Actinia equina* (Cnidaria: Anthozoa), the common starfish *Asterias rubens* (Echinodermata: Asteroidea), the dog whelk *Nucella lapillus* (Mollusca: Gastropoda), mixed earthworm species (*Amynthas* spp. and *Lumbricus* spp.; Annelida: Oligochaeta), and the brown alga *Fucus serratus* (Heterokonta: Phaecophyceae: Fucales). Marine species were sampled from rocky shores at Barns Ness (July 2014; 56.00° N, 2.45° E), and from three sites near Millport on the island of Great Cumbrae (August 2014; 55.77° N, 4.92° E) in Scotland, UK (S1 Table, S1 Text). The terrestrial sample (mixed earthworms; *Lumbricus* spp., and *Amythas* spp.), were collected from The King’s Buildings campus, Edinburgh, UK (November 2015; 55.92° N, 3.17° E). To maximise the probability of incorporating infected hosts, we included multiple individuals for sequencing (minimum: 37 sponge colonies; maximum: 164 starfish; see S1 Table for sampling details, numbers). Marine organisms were stored separately in sea water at 4°C for up to 72 hours before dissection. After dissection, the selected tissues were immediately frozen in liquid nitrogen, pooled in groups of 5–30 individuals, and ground to a fine powder for RNA extraction under liquid nitrogen (see S1 Text for details of tissue processing). Except for the brown alga *Fucus serratus*, RNA was extracted using Trizol (Life Technologies) and DNase treated (Turbo DNA-free: Life Technologies) following manufacturer’s instructions. For *Fucus*, the extraction protocol was modified from Apt *et al*., [162]. Briefly, tissue was lysed in a CTAB extraction buffer, and RNA was repeatedly (re-)extracted using chloroform/isoamyl alchohol (24:1) and phenol-chloroform (pH 4.3), and (re-)precipitated using 100% ethanol, 12M LiCl, and 3M NaOAc (pH 5.2).

### Library preparation and sequencing

To avoid potential nematode contamination, an aliquot of RNA from each small (5–30 individual) pool was reverse transcribed using M-MLV reverse transcriptase (Promega) with random hexamer primers. These were screened by PCR with nematode-specific primers and conditions as described in [163] (Forward 5'-CGCGAATRGCTCATTACAACAGC; Reverse 5'-GGCGATCAGATACCGCCC). We excluded all sample pools that tested positive for nematodes from sequencing, although they were used to infer virus prevalence (below). For each host species, RNA from the nematode-free pools were combined to give final RNA-sequencing pools in which individuals were approximately equally represented. For the sponge, sea anemone, starfish, and dog whelk this pooling was subsequently replicated, using a subset of the original small pools, resulting in sequencing pools ‘A’ and ‘B’ (S1 and S2 Tables).

Total RNA was provided to Edinburgh Genomics (Edinburgh, UK) for paired-end sequencing using the Illumina platform. Following ribosomal RNA depletion using Ribo-Zero Gold (Illumina), TruSeq stranded total RNAseq libraries (Illumina) were prepared using standard barcodes, to be sequenced in three groups, each on a single lane. Lanes were: (i) sponge, sea anemone, starfish, and dog whelk ‘A’ libraries (HiSeq v4; 125nt paired-end reads; a *Drosophila suzukii* RNAseq library from an unrelated project was also included in this lane); (ii) sponge, sea anemone, starfish, and dog whelk ‘B’ libraries (HiSeq 4000; 150nt paired-end reads); (iii) *Fucus* and Earthworms (HiSeq 4000; 150nt paired-end reads). In total, this resulted in approximately 70M high quality read pairs (i.e. after trimming and quality control) from the sponge, 60M from the sea anemone, 70M from the starfish, 70M from the dog whelk, 130M from the earthworms, and 180M from the brown alga (S3 Table).

For small RNA sequencing, total RNA was provided to Edinburgh Genomics (Edinburgh, UK) for untreated libraries (A and B), or after treatment either with a polyphosphatase (“A: Polyphosphatase”) or with sodium periodate (“B: Oxidised”). In the first case, we used a RNA 5' Polyphosphatase (Epicentre) treatment to convert 5' triphosphate groups to a single phosphate. This permits the ligation of small RNAs that result from direct synthesis rather than Dicer-mediated cleavage, such as 22G-RNA sRNAs of nematodes. In the second case, we used a sodium periodate (NaIO_4_) treatment (S2 Text). Oxidation using NaIO_4_ reduces the relative ligation efficiency of animal miRNAs that lack 3′-Ribose 2′O-methylation, relative to canonical piRNAs and viRNAs. This permits identification of 3′- 2′O-methylated sRNA populations, and is expected to enrich small RNA library for canonical piRNAs and viRNAs. TruSeq stranded total RNAseq libraries (Illumina) were prepared from treated RNA by Edinburgh Genomics, and sequenced using the Illumina platform (HiSeq v4; 50nt single-end reads), with all ‘A’ libraries sequenced together in a single lane, and all ‘B’ libraries sequenced together with *Fucus* and earthworm small RNAs, across four lanes. In total, this resulted in between 46M and 150M adaptor-trimmed small RNAs (S3 Table). Raw reads from RNAseq and small RNA sequencing are available from the NCBI Sequence Read Archive under accession number SRP153010, within BioProject accession PRJNA394213.

### Sequence assembly and taxonomic assignment

For each organism, paired end RNAseq data were assembled *de novo* using Trinity 2.2.0 [95,96] as a paired end strand-specific library (—SS_lib_type RF), following automated trimming (—trimmomatic) and digital read normalisation (—normalize_reads). Where two RNAseq libraries (‘A’ and ‘B’) had been sequenced, these were combined for assembly. For the mixed earthworm assembly, which had a large number of reads, high complexity, and a high proportion of ribosomal sequences (18%), ribosomal sequences were identified by mapping to a preliminary build of rRNA derived from subsampled data and excluded from the subsequent final assembly. To provide a low-resolution overview of the taxonomic diversity in each sample, we used Diamond [97] and BLASTp [164] to search the NCBI nr database using translated contigs, and MEGAN6 [98] (long reads with the weighted lowest common ancestor assignment algorithm) to provide taxonomic classification. In addition, for a more sensitive and quantitative analysis of Eukaryotic contamination, we recorded the number of Cytochrome oxidase reads for each reconstructed COI sequence present. To identify cytochrome oxidase 1 (COI) sequences, all COI DNA sequences from GenBank nt were used to search all contigs using BLASTn [164], and the resulting matches examined and manually curated before read mapping. An analogous approach was taken to identify rRNA sequences, but using rRNA from related taxa for a BLASTn search.

To identify probable virus and transposable element (TE)-like contigs, all long open reading frames from each contig were translated and concatenated to provide a ‘bait’ sequence for similarity searches using Diamond [97] and BLASTp [164]. Only those contigs with an open reading frame of at least 200 codons were retained. To reduce computing time, we used a two-step search. First, a preliminary search was made using translations against a Diamond database comprising all of the virus protein sequences available in NCBI database ‘nr’ (mode ‘blastp’; e-value 0.01; maximum of one match). Second, we used the resulting (potentially virus-like) contigs to search a Diamond database that combined all virus proteins from NCBI ‘nr’, with all proteins from NCBI ‘RefSeq_protein’ (mode ‘blastp’; e-value 0.01; no maximum matches). Putatively virus-like matches from this search were retained for manual examination and curation (including assessment of coverage—see below), resulting in 85 high-confidence putative virus contigs. A similar (but single-step) approach was used to search translated sequences from Repbase [99], using an e-value of 1x10^-10^ to identify TE-like contigs.

### Virus annotation and phylogenetic analysis

Translated open reading frames from the 85 virus-like contigs were used to search the NCBI ‘RefSeq_protein’ blast database using BLASTp [164]. High confidence open reading frames were manually annotated based on similarity to predicted (or known) proteins from related viruses. Where unlinked fragments could be unambiguously associated based on similarity to a related sequence or via PCR (below), they were assigned to the same virus. These contigs were provisionally named based on the collection location, host species, and virus lineage. Where available, the polymerase (or a polymerase component) from each putative virus species was selected for phylogenetic analysis. Where the polymerase was not present, sequences for phylogenetic analysis were selected to maximise the number of published virus sequences available. For the Weiviruses, bunya-like viruses, and noda-like viruses, two different proteins were used for phylogenetic inference. Published viral taxa were selected for inclusion based on high sequence similarity (identifiable by BLASTp). Translated protein sequences were aligned using T-Coffee [165] mode ‘m_coffee’ [166] combining a consensus of alignments from ClustalW [167,168], T-coffee [165], POA [169], Muscle [170], Mafft [171], DIALIGN [172], PCMA [173] and Probcons [174]. Alignments were examined by eye, and regions of ambiguous alignment at either end were removed. Phylogenetic relationships were inferred by maximum-likelihood using PhyML (version 20120412); (version 20120412; [175]) with the LG substitution model, empirical amino-acid frequencies, and a four-category gamma distribution of rates with an inferred shape parameter. Searches started from a maximum parsimony tree, and used both nearest-neighbour interchange (NNI) and sub-tree prune and re-graft (SPR) algorithms, retaining the best result. Support was assessed using the Shimodaira-Hasegawa-like nonparametric version of an approximate likelihood ratio test. All trees are presented mid-point rooted.

### PCR survey for virus prevalence

To estimate virus prevalence in the five animal taxa, we used a PCR survey of the small sample pools (5–30 individuals) for 53 virus-like contigs. There was insufficient RNA to survey prevalence in the brown alga. Aliquots from each sample pool were reverse transcribed using M-MLV reverse transcriptase (Promega) with random hexamer primers, and 10-fold diluted cDNA screened by PCR with primers for virus-like contigs designed using Primer3 [176,177]. To confirm that primer combinations could successfully amplify the target virus sequences, and to provide robust assays, each of four PCR assays (employing pairwise combinations of two forward and two reverse primers) were tested using combined pools of cDNA for each host, with the combination that produced the clearest amplicon band chosen as the optimal assay. We took a single successful PCR amplification to indicate the presence of virus in a pool, whereas absence was confirmed through at least 2 PCRs that produced no product. PCR primers and conditions are provided in S7 Table. Prevalence was inferred by maximum likelihood, and 2 log-likelihood intervals are reported.

### RT-negative PCR survey for EVE detection

For 47 of the putative RNA virus contigs, we used PCR to verify that the sequences were not present as DNA in our sample, i.e. were not EVEs. We performed an RT-negative PCR survey of Trizol RNA extractions (which also contained DNA) using the primers and conditions provided in S7 Table. Where amplification was successful from cDNA synthesised from a DNAse-treated extraction, but not from 1:10, 1:100, or 1:0000-fold diluted RNA samples (serial dilution was necessary as excessive RNA interfered with PCR), we inferred that template DNA was absent. The remaining six (out of a total of 53 contigs for which designed PCR assays) were putative parvo/denso-like virus contigs, and were also tested as above. All six DNA virus contigs were detectable as DNA copies.

### Origin of sequencing reads and small RNA properties

To identify the origin of RNA sequencing reads, quality trimmed forward-orientation RNAseq reads and adaptor-trimmed small-RNA reads between 17nt and 40nt in length (and trimmed using cutadapt and retaining adaptor triimed reads only; [178]) were mapped to potential source sequences. To provide approximate counts of rRNA and miRNA reads, reads were mapped to ribosomal contigs from the target host taxa and to all mature miRNA stem-loops represented in miRbase [124], using Bowtie2 [179] with the ‘—fast’ sensitivity option and retaining only one mapping (option ‘-k 1’). To identify the number and properties of virus and TE-derived reads, the remaining unmapped reads were then mapped to the 85 curated virus-like contigs, to COI-like contigs, and to 146 selected long TE-like contigs between 2kbp and 7.5kbp from out assemblies, using the ‘—sensitive’ option and default reporting (multiple alignments, report mapping quality). For small RNA mapping, the gap-opening and extension costs were set extremely high (‘—rdg 20,20—rfg 20,20’) to exclude maps that required an indel. The resulting read mappings were counted and analysed for the distribution of read lengths, base composition, and orientation. In an attempt to identify modified or edited small RNAs, we additionally mapped the small RNA reads to the virus-like and TE-like contigs using high sensitivity local mapping options equivalent to ‘—very-sensitive-local’ but additionally permitting a mismatch in the mapping seed region (‘-N 1’) and again preventing indels (‘—rdg 20,20—rfg 20,20’). We also reanalysed the data using only perfect (zero mismatch) mappings. Neither approach led to substantially different results.

## Supporting information

S1 Fig Taxonomic composition of contigs. For each of the six organisms, the coloured bars show (on a linear scale), the proportion of all Trinity contigs assigned to each major lineage using Diamond [97] and MEGAN6 [98] with ‘long reads’.
(PDF)

S2 Fig Phylogenetic trees. Maximum likelihood phylogenetic trees. Support values (approximate likelihood ratio test) and NCBI accession identifiers are provided. Viruses newly identified here are highlighted in red, and unannotated virus-like sequences from publicly-available transcriptome datasets are denoted ‘TSA’. Clade names follow [100,104]. Alignments are provided in S2 Data and Newick format trees in S3 Data.
(PDF)

S3 Fig Relative read counts from high- copy-number viruses. The bar plot shows the relative number of RNAseq reads that mapped to each of virus contigs, as a percentage relative to the read count of host COI reads (both normalised by contig length). Both positive and negative sense reads were included, from library ‘B’ only. Viruses with less than 0.01% of the COI read count were excluded. Contigs marked in bold and italic are thought to be DNA viruses or endogenous viral elements, and contigs marked with an asterisk were surveyed by (RT-PCR). Those contigs that were a source of detectable small RNAs are marked ‘viRNA’ or ‘piRNA’ as appropriate.
(PDF)

S4 Fig Strand bias in RNA sequencing reads from viruses. Bars show the proportion of RNA sequencing reads (combined across libraries) derived from the positive (sense) strand of the virus, for all viruses represented by >150 read pairs. Error bars reflect 95% confidence intervals based on a likelihood ratio test, assuming a binomial distribution, and to clearly display ratios close to zero and one the results are plotted on a logit scale. All -ssRNA viruses and dsRNA viruses show strong evidence of replication, as their respective proportions of positive sense reads are >>0% and >>50%. Many of the +ssRNA viruses show evidence of replication, as the proportion of positive reads is <100%. However, the positive to negative strand ratio for replicating +ssRNA reads can be very high, making this a conservative test. Note that two of the putative DNA parvovirus EVEs display negative sense reads, constant with host-driven expression rather than functional mRNA expression.
(PDF)

S5 Fig Size distributions of small RNAs. Bar-plot size distributions of all small RNAs sequences. Columns correspond to species, rows to libraries. **Panel A**: All sRNAs from each library. **B**: sRNAs mapping to ribosomal sequences. Note that in most species read abundance decreases with size, indicative of degradation products, but that distinct peaks are visible in the oxidised libraries, consistent with specific short rRNAs possessing a 3' 2-O-methyl group. **C**: sRNAs mapping to known miRNA stem-loops from miRbase [124]. The proportion of putative miRNAs decreases dramatically in all oxidised libraries except the sea anemone, suggesting that miRNAs in this species possess 3' 2-O-methyl groups. The small number of mapped miRNA reads in the brown alga is probably a result of the under-representation of close relatives in miRbase [124]. **D**: sRNAs mapping to putative RNA virus contigs. Only the dog whelk has a large and distinctive distribution of virus-derived sRNAs, and these increase in the oxidised library, suggesting that they possess 3' 2-O-methyl groups. The small number of very short virus-derived reads in the sponge are consistent with degradation products. **E**: sRNAs mapping to DNA parvovirus-like contigs. These increase in the oxidised library, suggesting that they possess 3' 2-O-methyl groups. **F**: sRNAs mapping to selected TE-like contigs. These vary in their size range among species (21nt in the brown alga, bimodal in the sponge, peaking at 28-29nt in the other species), and increase in the oxidised library, suggesting that they possess 3'-2-O-methyl groups. Only a small proportion of TE-like contigs were used as mapping targets, and many TE-derived small RNAs remain unmapped. **G**: Unmapped sRNAs, comprising those that derived from divergent miRNAs, unrecognised viral contigs, TEs that were excluded from panel F, and all other sources. The data required to plot these figs are provided in S5 Table.
(PDF)

S6 Fig Properties and repeatability of virus-derived small RNAs. Panels **A-D** are dog-whelk RNA viruses, panels **E-H** starfish DNA virus-like contigs, panel **I** is the anemone DNA virus, and panel **J** is the brown alga virus (note that only one library was made for this sample). In each panel, rows (top to bottom) represent each library: Library A, polyphosphatase-treated library A, Library B, and oxidised library B. Columns (left to right) are (i) Origin of reads from each genome position (red lines above the x-axis denote reads from the positive sense strand, blue lines below the x-axis denote reads from the negative sense strand; (ii) Bar plot of frequencies of unique sequences, bars above the x-axis denote reads from the positive sense strand, those below the x-axis denote reads from the negative sense strand, colours indicate 5' base (U red, G yellow, C blue and A green); (iii) Barplot of frequencies of reads; (iv) Sequence logo for the unique sequences of the most frequent length deriving from the positive strand; (v) Sequence logo for the unique sequences of the most frequent length deriving from the negative strand. The data required to plot the size distributions are provided in S5 Table.
(PDF)

S7 Fig Small RNAs mapping to bacterial contigs. Columns show (left) the number and size distribution of small RNAs that mapped to contigs provisionally classified as bacterial by similarity search, and the hotspots and size distributions for the nominal bacterial contig that displayed the largest (centre) and second-largest (right) number of small RNA mappings. The combined oxidised libraries are shown for all species except the brown alga (untreated library, as no oxidised library was prepared), and colours and axes are as in Figs 3–5. Read numbers were very small for the Starfish, Dog Whelk and Sea Anemone (<2000 reads) and in Fucus the vast majority of small RNAs derived from a bacterial rRNA, but their size distribution suggests that they represent degradation products. Those in the Earthworm and the Sponge strongly resemble host primary piRNAs in their strand bias, size distribution and base composition (compare with Fig 5), but manual inspection suggests that almost all derived from misclassified host TE contigs.
(PDF)

S8 Fig Properties and repeatability of TE-derived small RNAs. Panels **A-R** show the small RNA properties of selected high-confidence TE-like contigs: starfish panels **A-C**, dog whelk **D-F**, sponge **G-I**, earthworms **J-L**, sea anemone **M-O**, brown alga **P-R**. Rows and columns are as in S6 Fig. The data required to plot the size distributions are provided in S5 Table.
(PDF)

S9 Fig RNAseq and sRNA reads per metagenomic contig. For each metagenomic contig (pale grey) the ratio of sRNAs (20-31nt) to RNAseq reads is shown on the x-axis, and the ratio of 20-24nt sRNAs (expected viRNAs) to 25-31nt sRNAs (expected piRNAs) is shown on the y-axis. Contigs are only included if they are >0.75Kbp in length and produced at least 20 small RNAs; Contigs in dark grey have sequence similarity to known TEs, and contigs in colour correspond to the curated viruses. Based on *Drosophila*, TEs (dark grey) are expected to appear in the lower right quadrant of each plot, and viruses (colour) in the upper right [see 149]. Only the dog whelk (panel **A**) and the brown alga (panel **D**) display sRNAs from RNA virus contigs, although DNA virus-like contigs display piRNA-like small RNAs in the sea anemone (panel **C**) and the starfish (panel **B**). No other viruses produced sufficient viRNAs to appear on these figs. All figures (except the brown alga) use data from RNAseq library B and the corresponding oxidised sRNAs (which is enriched for viRNAs over miRNAs), and sRNA counts exclude those mapping to known (miRbase) miRNAs and rRNAs.
(PDF)

S1 Table Sample collection details. Detailed descriptions of the sample collection locations, dates and numbers of individuals sampled for each target taxon, along with sample pool information, including which extraction pools were included in sequencing pools, and which were excluded due to detection of suspected nematode contamination.
(XLSX)

S2 Table Detailed descriptions of putative viruses and virus-like contigs. Detailed descriptions of the candidate virus fragments identified by protein similarity search, including phylogenetic position, estimated prevalence, approximate coverage, ORF number and most similar viral proteins identified by BLASTp, detectability by RT-negative PCR, GenBank accession numbers, and additional notes.
(XLSX)

S3 Table Sources of RNAseq and small RNA reads. Cytochrome oxidase coverage relative to that of the target taxon, and virus coverage for the target viruses (positive and negative strand) relative to that of COI.
(XLSX)

S4 Table Virus prevalence. Estimated virus prevalence inferred by maximum likelihood (with 2 log-likelihood intervals) from an RT-PCR survey of pooled samples[methods in 149].
(XLSX)

S5 Table Size distribution of small RNAs. Raw counts necessary to plot Figs 3, 4 and 5, S4, S5 and S6 Figs.
(XLSX)

S6 Table RNAi related genes identified from organisms. Counts of key RNAi related genes identified in transcriptomes of target taxa along with GenBank accession numbers for sequences.
(XLSX)

S7 Table PCR primers and conditions. PCR primer names and sequences, thermocycler conditions, and PCR recipes for virus prevalence, and RT-negative (EVE detection), assays.
(XLSX)

S1 Data Putative virus-like contigs. Raw meta-transcriptomic contigs generated by Trinity that have detectable sequence similarity (using Diamond) to virus proteins in GenBank, provided in compressed (gzipped) fasta format. Contig titles are annotated using the species name of the top match, followed by the percentage identity of that match in the sequence, and the e-value associated with that match. The contigs have not been curated, and are likely to include chimeric assemblies. As such, they are not suitable for submission to GenBank, and should be treated with caution.
(GZ) Click here for additional data file.

S2 Data Protein sequence alignments. Protein sequence alignments used for phylogenetic analyses are provided in compressed (gzipped) gapped fasta format, with regions of poor alignment (identified by eye) deleted. Sequence titles comprise the taxon name and NCBI accession identifier for the sequence.
(TGZ)

S3 Data Phylogenetic trees. Phylogenetic trees are provided in compressed (gzipped) newick format. Sequence titles comprise the taxon name and NCBI accession identifier for the original protein sequence.
(TGZ)

S4 Data Long high-confidence TE-like contigs. Selected meta-transcriptomic contigs generated by Trinity that have detectable sequence similarity (using Diamond) to TEs in Repbase [99], provided in compressed (gzipped) fasta format. Contig titles are annotated using the host species name and the top-match TE in Repbase.
(GZ) Click here for additional data file.

S1 Text Sampling, tissue preparations and RNA extractions. Detailed description of the sampling, tissue preparations and RNA extractions techniques employed for each target taxon.
(PDF)

S2 Text RNA oxidation treatment. Protocol for sodium periodate (NaIO4) oxidation of RNA prior to library preparation, to enrich small RNA libraries for canonical piRNAs and viRNAs by reducing the relative ligation efficiency of metazoan miRNAs that lack 3′-Ribose 2′O-methylation.
(PDF)

## References

[1] Obbard DJ, Gordon KHJ, Buck AH, Jiggins FM (2009) The evolution of RNAi as a defence against viruses and transposable elements. Philos Trans R Soc Lond B Biol Sci 364: 99–115. 10.1098/rstb.2008.0168 18926973 PMC2592633

[2] Cerutti H, Casas-Mollano JA (2006) On the origin and functions of RNA-mediated silencing: from protists to man. Curr Genet 50: 81–99. 10.1007/s00294-006-0078-x 16691418 PMC2583075

[3] Ding SW, Li H, Lu R, Li F, Li WX (2004) RNA silencing: A conserved antiviral immunity of plants and animals. Virus Res 102: 109–115. 10.1016/j.virusres.2004.01.021 15068886

[4] Buchon N, Vaury C (2006) RNAi: a defensive RNA-silencing against viruses and transposable elements. Heredity (Edinb) 96: 195–202. 10.1038/sj.hdy.6800789 16369574

[5] Segers GC, Zhang X, Deng F, Sun Q, Nuss DL (2007) Evidence that RNA silencing functions as an antiviral defense mechanism in fungi. Proc Natl Acad Sci 104: 12902–12906. 10.1073/pnas.0702500104 17646660 PMC1937564

[6] Chang S-S, Zhang Z, Liu Y (2012) RNA interference pathways in Fungi: mechanisms and functions. Annu Rev Microbiol 66: 305–323. 10.1146/annurev-micro-092611-150138 22746336 PMC4617789

[7] Agius C, Eamens AL, Millar AA, Watson JM, Wang M (2012) Antiviral Resistance in Plants In: Watson J, Wang M-B, editors. Antiviral Resistance in Plants: Methods and Protocols. Methods In Molecular Biology. Springer International Publishing, Vol. 894 pp. 17–38. 10.1007/978-1-61779-882-5

[8] Axtell MJ (2013) Classification and comparison of small RNAs from plants. Annu Rev Plant Biol 64: 137–159. 10.1146/annurev-arplant-050312-120043 23330790

[9] Szittya G, Burgyan J (2013) RNA interference-mediated intrinsic antiviral immunity in plants. Curr Top Microbiololgy Immunol 371: 153–181. 10.1007/978-3-642-37765-5_6 23686235

[10] Dang Y, Yang Q, Xue Z, Liu Y (2011) RNA interference in fungi: pathways, functions, and applications. Eukaryot Cell 10: 1148–1155. 10.1128/EC.05109-11 21724934 PMC3187057

[11] Borges F, Martienssen RA (2015) The expanding world of small RNAs in plants. Nat Rev Mol Cell Biol 16: 727–741. 10.1038/nrm4085 26530390 PMC4948178

[12] Nicolás FE, Ruiz-Vázquez RM (2013) Functional diversity of RNAi-associated sRNAs in fungi. Int J Mol Sci 14: 15348–15360. 10.3390/ijms140815348 23887655 PMC3759863

[13] Sarkies P, Miska E a (2013) RNAi pathways in the recognition of foreign RNA: antiviral responses and host-parasite interactions in nematodes. Biochem Soc Trans 41: 876–880. 10.1042/BST20130021 23863148

[14] Barnard AC, Nijhof AM, Fick W, Stutzer C, Maritz-Olivier C (2012) RNAi in arthropods: Insight into the machinery and applications for understanding the pathogen-vector interface. Genes (Basel) 3: 702–741. 10.3390/genes3040702 24705082 PMC3899984

[15] Grimson A, Srivastava M, Fahey B, Woodcroft BJ, Chiang HR, et al (2008) Early origins and evolution of microRNAs and Piwi-interacting RNAs in animals. Nature 455: 1193–1197. 10.1038/nature07415 18830242 PMC3837422

[16] Praher D, Zimmermann B, Genikhovich G, Columbus-Shenkar Y, Modepalli V, et al (2017) Characterization of the piRNA pathway during development of the sea anemone *Nematostella vectensis*. RNA Biol 14: 1727–1741. 10.1080/15476286.2017.1349048 28783426 PMC5731801

[17] Lewis SH, Quarles KA, Yang Y, Tanguy M, Frézal L, et al (2018) Pan-arthropod analysis reveals somatic piRNAs as an ancestral defence against transposable elements. Nat Ecol Evol 2: 174–181. 10.1038/s41559-017-0403-4 29203920 PMC5732027

[18] Calcino AD, Fernandez-valverde SL, Taft RJ, Degnan BM (2018) Diverse RNA interference strategies in early-branching metazoans. BioRXiv. 10.1186/s12862-018-1274-2 PMC6211395 30382896

[19] Yamanaka S, Siomi MC, Siomi H (2014) piRNA clusters and open chromatin structure. Mob DNA 5: 22 10.1186/1759-8753-5-22 25126116 PMC4131230

[20] Czech B, Hannon GJ (2016) One loop to rule them all: the ping-pong cycle and piRNA-guided silencing. Trends Biochem Sci 41 10.1016/j.tibs.2015.12.008 26810602 PMC4819955

[21] Baum J, Papenfuss AT, Mair GR, Janse CJ, Vlachou D, et al (2009) Molecular genetics and comparative genomics reveal RNAi is not functional in malaria parasites. Nucleic Acids Res 37: 3788–3798. 10.1093/nar/gkp239 19380379 PMC2699523

[22] Lye LF, Owens K, Shi H, Murta SMF, Vieira AC, et al (2010) Retention and Loss of RNA interference pathways in trypanosomatid protozoans. PLoS Pathog 6: e1001161 10.1371/journal.ppat.1001161 21060810 PMC2965760

[23] Drinnenberg I a, Weinberg DE, Xie KT, Mower JP, Wolfe KH, et al (2009) RNAi in budding yeast. Science 326: 544–550. 10.1126/science.1176945 19745116 PMC3786161

[24] Backes S, Langlois RA, Schmid S, Varble A, Shim J V., et al (2014) The mammalian response to virus infection is Independent of small RNA silencing. Cell Rep 8: 114–125. 10.1016/j.celrep.2014.05.038 24953656 PMC4096324

[25] Bogerd HP, Skalsky RL, Kennedy EM, Furuse Y, Whisnant AW, et al (2014) Replication of many human viruses is refractory to inhibition by endogenous cellular microRNAs. J Virol 88: 8065–8076. 10.1128/JVI.00985-14 24807715 PMC4097787

[26] Parameswaran P, Sklan E, Wilkins C, Burgon T, Samuel M a, et al (2010) Six RNA viruses and forty-one hosts: viral small RNAs and modulation of small RNA repertoires in vertebrate and invertebrate systems. PLoS Pathog 6: e1000764 10.1371/journal.ppat.1000764 20169186 PMC2820531

[27] Umbach JL, Cullen BR (2009) The role of RNAi and microRNAs in animal virus replication and antiviral immunity. Genes Dev 23: 1151–1164. 10.1101/gad.1793309 19451215 PMC2763533

[28] Girardi E, Chane-Woon-Ming B, Messmer M, Kaukinen P, Pfeffer S (2013) Identification of RNase L-dependent, 3′ -end-modified, viral small RNAs in Sindbis virus-infected mammalian cells. MBio 4: 1–10. 10.1128/mBio.00698-13 24255120 PMC3870239

[29] Backes S, Langlois RA, Schmid S, Varble A, Shim J V., et al (2014) The mammalian response to virus infection is independent of small RNA silencing. Cell Rep 8: 114–125. 10.1016/j.celrep.2014.05.038 24953656 PMC4096324

[30] Perez JT, Varble A, Sachidanandam R, Zlatev I, Manoharan M, et al (2010) Influenza A virus-generated small RNAs regulate the switch from transcription to replication. Proc Natl Acad Sci 107: 11525–11530. 10.1073/pnas.1001984107 20534471 PMC2895093

[31] Li Y, Lu J, Han Y, Fan X, Ding S-W (2013) RNA interference functions as an antiviral immunity mechanism in mammals. Science 342: 231–234. 10.1126/science.1241911 24115437 PMC3875315

[32] Maillard P V, Ciaudo C, Marchais A, Li Y, Jay F, et al (2013) Antiviral RNA interference in mammalian cells. Science 342: 235–238. 10.1126/science.1241930 24115438 PMC3853215

[33] Maillard P V, Van Der Veen AG, Deddouche-grass S, Rogers NC (2016) Inactivation of the type I interferon pathway reveals long double-stranded RNA-mediated RNA interference in mammalian cells. EMBO J: 1–14. 10.15252/embj.201695086 27815315 PMC5167344

[34] Li Y, Basavappa M, Lu J, Dong S, Cronkite DA, et al (2016) Induction and suppression of antiviral RNA interference by influenza A virus in mammalian cells. Nat Microbiol 2: 16250 10.1038/nmicrobiol.2016.250 27918527 PMC5488270

[35] Qiu Y, Xu Y, Zhang Y, Zhou H, Deng Y-Q, et al (2017) Human virus-derived small RNAs can confer antiviral immunity in mammals. Immunity 46: 992–1004.e5. 10.1016/j.immuni.2017.05.006 28636969

[36] Jeffrey KL, Li Y, Ding S (2017) Reply to ‘Questioning antiviral RNAi in mammals.’ Nat Microbiol 2: 17053 10.1038/nmicrobiol.2017.53 28440274 PMC5488271

[37] tenOever BR (2017) Questioning antiviral RNAi in mammals. Nat Microbiol 2: 17052 10.1038/nmicrobiol.2017.52 28440277

[38] Bronkhorst AW, van Rij RP (2014) The long and short of antiviral defense: small RNA-based immunity in insects. Curr Opin Virol 7C: 19–28. 10.1016/j.coviro.2014.03.010 24732439

[39] Gammon D, Mello C (2015) RNA interference-mediated antiviral defense in insects. Curr Opin Insect Sci: 111–120. 10.1016/j.cois.2015.01.006 26034705 PMC4448697

[40] Labreuche Y, Warr GW (2013) Insights into the antiviral functions of the RNAi machinery in penaeid shrimp. Fish Shellfish Immunol 34: 1002–1010. 10.1016/j.fsi.2012.06.008 22732509

[41] Liu H, Söderhäll K, Jiravanichpaisal P (2009) Antiviral immunity in crustaceans. Fish Shellfish Immunol 27: 79–88. 10.1016/j.fsi.2009.02.009 19223016 PMC7172356

[42] Schnettler E, Tykalová H, Watson M, Sharma M, Sterken MG, et al (2014) Induction and suppression of tick cell antiviral RNAi responses by tick-borne flaviviruses. Nucleic Acids Res 42: 1–11. 25053841 10.1093/nar/gku657 PMC4132761

[43] Ashe A, Bélicard T, Le Pen J, Sarkies P, Frézal L, et al (2013) A deletion polymorphism in the *Caenorhabditis elegans* RIG-I homolog disables viral RNA dicing and antiviral immunity. Elife 2: e00994 10.7554/eLife.00994 24137537 PMC3793227

[44] Gammon DB, Ishidate T, Li L, Gu W, Silverman N, et al (2017) The antiviral RNA interference response provides resistance to lethal arbovirus infection and vertical transmission in *Caenorhabditis elegans*. Curr Biol 27: 795–806. 10.1016/j.cub.2017.02.004 28262484 PMC5446062

[45] Coffman S, Lu J, Guo X, Zhong J, Jiang H, et al (2017) *Caenorhabditis elegans* RIG-1 homolog mediates antiviral RNA interference downstream of dicer-dependent biogenesis of viral small interfering RNAs. MBio 8: 1–15. 10.1128/mBio.00264-17 PMC5362034 28325765

[46] Seo GJ, Kincaid RP, Phanaksri T, Burke JM, Pare JM, et al (2013) Reciprocal inhibition between intracellular antiviral signaling and the RNAi machinery in mammalian cells. Cell Host Microbe 14. 10.1016/j.chom.2013.09.002 PMC3837626 24075860

[47] Mukherjee K, Campos H, Kolaczkowski B (2013) Evolution of animal and plant dicers: early parallel duplications and recurrent adaptation of antiviral RNA binding in plants. Mol Biol Evol 30: 627–641. 10.1093/molbev/mss263 23180579 PMC3563972

[48] Tabach Y, Billi AC, Hayes GD, Newman M a, Zuk O, et al (2013) Identification of small RNA pathway genes using patterns of phylogenetic conservation and divergence. Nature 493: 694–698. 10.1038/nature11779 23364702 PMC3762460

[49] Casas-Mollano JA, Zacarias E, Ma X, Kim E-J, Cerutti H (2016) RNA-Mediated Silencing in Eukaryotes: Evolution of the protein synthesis machinery and its regulation In: Hernanez G, Jagus R, editors. Evolution of the Protein Synthesis Machinery and Its Regulation. Springer International Publishing pp. 513–529.

[50] De Jong D, Eitel M, Jakob W, Osigus HJ, Hadrys H, et al (2009) Multiple Dicer genes in the early-diverging metazoa. Mol Biol Evol 26: 1333–1340. 10.1093/molbev/msp042 19276153

[51] Rajasethupathy P, Antonov I, Sheridan R, Frey S, Sander C, et al (2012) A role for neuronal piRNAs in the epigenetic control of memory-related synaptic plasticity. Cell 149: 693–707. 10.1016/j.cell.2012.02.057 22541438 PMC3442366

[52] Castel SE, Martienssen R a (2013) RNA interference in the nucleus: roles for small RNAs in transcription, epigenetics and beyond. Nat Rev Genet 14: 100–112. 10.1038/nrg3355 23329111 PMC4205957

[53] Lee YS, Nakahara K, Pham JW, Kim K, He Z, et al (2004) Distinct roles for *Drosophila* Dicer-1 and Dicer-2 in the siRNA/miRNA silencing pathways. Cell 117: 69–81. 15066283 10.1016/s0092-8674(04)00261-2

[54] Grishok A, Pasquinelli AE, Conte D, Li N, Parrish S, et al (2001) Genes and mechanisms related to RNA interference regulate expression of the small temporal RNAs that control *C*. *elegans* developmental timing. Cell 106: 23–34. 10.1016/S0092-8674(01)00431-7 11461699

[55] Tabara H, Yigit E, Siomi H, Mello CC (2002) The double-stranded RNA binding protein RDE-4 interacts in vivo with RDE-1, DCR-1 and a conserved DExH-box helicase to direct RNA interference in *C*. *elegans*. Cell 109: 861–871. 12110183 10.1016/s0092-8674(02)00793-6

[56] Lewis SH, Salmela H, Obbard DJ, Street D, Buildings K, et al (2015) Duplication and diversification of Dipteran Argonaute genes, and the evolutionary divergence of Piwi and Aubergine: 1–30. 10.1093/gbe/evw018 26868596 PMC4824172

[57] Sarkies P, Selkirk ME, Jones JT, Blok V, Boothby T, et al (2015) Ancient and novel small RNA pathways compensate for the loss of piRNAs in multiple independent nematode lineages. PLOS Biol 13: e1002061 10.1371/journal.pbio.1002061 25668728 PMC4323106

[58] Mondal M, Klimov P, Flynt AS (2018) Rewired RNAi-mediated genome surveillance in house dust mites. PLOS Genet 14: e1007183 10.1371/journal.pgen.1007183 29377900 PMC5805368

[59] Swarts DC, Makarova K, Wang Y, Nakanishi K, Ketting RF, et al (2014) The evolutionary journey of Argonaute proteins. Nat Struct Mol Biol 21: 743–753. 10.1038/nsmb.2879 25192263 PMC4691850

[60] Burroughs AM, Ando Y, Aravind L (2014) New perspectives on the diversification of the RNA interference system: Insights from comparative genomics and small RNA sequencing. Wiley Interdiscip Rev RNA 5: 141–181. 10.1002/wrna.1210 24311560 PMC4066877

[61] Yigit E, Batista PJ, Bei Y, Pang KM, Chen CCG, et al (2006) Analysis of the *C*. *elegans* Argonaute family reveals that distinct Argonautes act sequentially during RNAi. Cell 127: 747–757. 10.1016/j.cell.2006.09.033 17110334

[62] Pak J, Fire A (2007) Distinct populations of primary and secondary effectors during RNAi in *C*. *elegans*. Science (80-) 315: 241–244. 10.1126/science.1132839 17124291

[63] Morazzani EM, Wiley MR, Murreddu MG, Adelman ZN, Myles KM (2012) Production of virus-derived ping-pong-dependent piRNA-like small RNAs in the mosquito soma. PLoS Pathog 8: e1002470 10.1371/journal.ppat.1002470 22241995 PMC3252369

[64] Vodovar N, Bronkhorst AW, van Cleef KWR, Miesen P, Blanc H, et al (2012) Arbovirus-derived piRNAs exhibit a ping-pong signature in mosquito cells. PLoS One 7: e30861 10.1371/journal.pone.0030861 22292064 PMC3265520

[65] Goic B, Vodovar N, Mondotte JA, Monot C, Frangeul L, et al (2013) RNA-mediated interference and reverse transcription control the persistence of RNA viruses in the insect model *Drosophila*. Nat Immunol 14: 396–403. 10.1038/ni.2542 23435119

[66] Tassetto M, Kunitomi M, Tassetto M, Kunitomi M, Andino R (2017) Circulating immune cells mediate a systemic RNAi-based adaptive antiviral response in Drosophila. Cell 169: 314–325.e13. 10.1016/j.cell.2017.03.033 28388413 PMC5730277

[67] Poirier EZ, Goic B, Tomé-Poderti L, Frangeul L, Boussier J, et al (2018) Dicer-2-dependent generation of viral DNA from defective genomes of RNA viruses modulates antiviral immunity in insects. Cell Host Microbe 23: 353–365.e8. 10.1016/j.chom.2018.02.001 29503180 PMC5857290

[68] tenOever BR (2016) The evolution of antiviral defense systems. Cell Host Microbe 19: 142–149. 10.1016/j.chom.2016.01.006 26867173

[69] Koonin E V. (2017) Evolution of RNA- and DNA-guided antivirus defense systems in prokaryotes and eukaryotes: common ancestry vs convergence. Biol Direct 12: 5 10.1186/s13062-017-0177-2 28187792 PMC5303251

[70] Moran Y, Agron M, Praher D, Technau U (2017) The evolutionary origin of plant and animal microRNAs. Nat Ecol Evol 1: 0027 10.1038/s41559-016-0027 28529980 PMC5435108

[71] Funayama N, Nakatsukasa M, Mohri K, Masuda Y, Agata K (2010) Piwi expression in archeocytes and choanocytes in demosponges: Insights into the stem cell system in demosponges. Evol Dev 12: 275–287. 10.1111/j.1525-142X.2010.00413.x 20565538

[72] Juliano CE, Reich A, Liu N, Gotzfried J, Zhong M, et al (2013) PIWI proteins and PIWI-interacting RNAs function in *Hydra* somatic stem cells. Proc Natl Acad Sci 111: 337–342. 10.1073/pnas.1320965111 24367095 PMC3890812

[73] Alié A, Leclère L, Jager M, Dayraud C, Chang P, et al (2011) Somatic stem cells express Piwi and Vasa genes in an adult ctenophore: Ancient association of “germline genes” with stemness. Dev Biol 350: 183–197. 10.1016/j.ydbio.2010.10.019 21036163

[74] Aravin A, Gaidatzis D, Pfeffer S, Lagos-Quintana M, Landgraf P, et al (2006) A novel class of small RNAs bind to MILI protein in mouse testes. Nature 442: 203–207. 10.1038/nature04916 16751777

[75] Houwing S, Kamminga LM, Berezikov E, Cronembold D, Girard A, et al (2007) A role for piwi and piRNAs in germ cell baintenance and transposon silencing in Zebrafish. Cell 129: 69–82. 10.1016/j.cell.2007.03.026 17418787

[76] Brennecke J, Aravin AA, Stark A, Dus M, Kellis M, et al (2007) Discrete small RNA-generating loci as master regulators of transposon activity in *Drosophila*. Cell 128: 1089–1103. 10.1016/j.cell.2007.01.043 17346786

[77] Swarts DC, Szczepaniak M, Sheng G, Chandradoss SD, Zhu Y, et al (2017) Autonomous generation and loading of DNA guides by bacterial Argonaute. Mol Cell: 985–998. 10.1016/j.molcel.2017.01.033 28262506 PMC5779613

[78] Miesen P, Girardi E, van Rij RP (2015) Distinct sets of PIWI proteins produce arbovirus and transposon-derived piRNAs in *Aedes aegypti* mosquito cells. Nucleic Acids Res 43: 6545–6556. 26068474 10.1093/nar/gkv590 PMC4513867

[79] Cai Y, Zhou Q, Yu C, Wang X, Hu S, et al (2012) Transposable-element associated small RNAs in Bombyx mori genome. PLoS One 7: e36599 10.1371/journal.pone.0036599 22662121 PMC3359762

[80] Sijen T, Plasterk H (2003) Transposon silencing in the *Caenorhabditis elegans* germ by natural RNAi. Nature 426: 310–314. 10.1038/nature02107 14628056

[81] Zhou X, Battistoni G, El Demerdash O, Gurtowski J, Wunderer J, et al (2015) Dual functions of Macpiwi1 in transposon silencing and stem cell maintenance in the flatworm *Macrostomum lignano*. RNA 21: 1885–1897. 10.1261/rna.052456.115 26323280 PMC4604429

[82] Ishikawa T, Nishikawa H, Gao Y, Sawa Y, Shibata H, et al (2008) The pathway via D-galacturonate/L-galactonate is significant for ascorbate biosynthesis in *Euglena gracilis*: Identification and functional characterization of aldonolactonase. J Biol Chem 283: 31133–31141. 10.1074/jbc.M803930200 18782759 PMC2662179

[83] Takahashi F, Yamagata D, Ishikawa M, Fukamatsu Y, Ogura Y, et al (2007) AUREOCHROME, a photoreceptor required for photomorphogenesis in stramenopiles. Proc Natl Acad Sci 104: 19625–19630. 10.1073/pnas.0707692104 18003911 PMC2148339

[84] Kaur G, Lohia A (2004) Inhibition of gene expression with double strand RNA interference in *Entamoeba histolytica*. Biochem Biophys Res Commun 320: 1118–1122. 10.1016/j.bbrc.2004.06.064 15249205

[85] Ngo H, Tschudi C, Gull K, Ullu E (1998) Double-stranded RNA induces mRNA degradation in Trypanosoma brucei. Proc Natl Acad Sci 95: 14687–14692. 10.1073/pnas.95.25.14687 9843950 PMC24510

[86] Rivera AS, Hammel JU, Haen KM, Danka ES, Cieniewicz B, et al (2011) RNA interference in marine and freshwater sponges: actin knockdown in *Tethya wilhelma* and *Ephydatia muelleri* by ingested dsRNA expressing bacteria. BMC Biotechnol 11: 67 10.1186/1472-6750-11-67 21679422 PMC3146823

[87] Wittig K, Kasper J, Seipp S, Leitz T (2011) Evidence for an instructive role of apoptosis during the metamorphosis of *Hydractinia echinata* (Hydrozoa). Zoology 114: 11–22. 10.1016/j.zool.2010.09.004 21247747

[88] Jakob W, Sagasser S, Dellaporta S, Holland P, Kuhn K, et al (2004) The Trox-2 Hox/ParaHox gene of *Trichoplax* (Placozoa) marks an epithelial boundary. Dev Genes Evol 214: 170–175. 10.1007/s00427-004-0390-8 14997392

[89] Yu N, Christiaens O, Liu J, Niu J, Cappelle K, et al (2013) Delivery of dsRNA for RNAi in insects: An overview and future directions. Insect Sci 20: 4–14. 10.1111/j.1744-7917.2012.01534.x 23955821

[90] Fire A, Xu S, Montgomery M, Kostas S, Driver S, et al (1998) Potent and specifc genetic interference by double-stranded RNA in Caenorhabditis elegans. Nature 394: 806–811. 10.1038/35888 9486653

[91] Sánchez Alvarado A, Newmark P (1999) Double-stranded RNA specifically disrupts gene expression during planarian regeneration. Proc Natl Acad Sci U S A 96: 5049–5054. 10.1073/pnas.96.9.5049 10220416 PMC21814

[92] Fabioux C, Corporeau C, Quillien V, Favrel P, Huvet A (2009) In vivo RNA interference in oyster -vasa silencing inhibits germ cell development. FEBS J 276: 2566–2573. 10.1111/j.1742-4658.2009.06982.x 19476495

[93] Snell TW, Shearer TL, Smith HA (2011) Exposure to dsRNA elicits RNA interference in Brachionus manjavacas (Rotifera). Mar Biotechnol 13: 264–274. 10.1007/s10126-010-9295-x 20461431

[94] Yoshida-Noro C, Tochinai S (2010) Stem cell system in asexual and sexual reproduction of *Enchytraeus japonensis* (Oligochaeta, Annelida). Dev Growth Differ 52: 43–55. 10.1111/j.1440-169X.2009.01149.x 20039928

[95] Grabherr MG, Haas BJ, Yassour M, Levin JZ, Thompson D a, et al (2011) Full-length transcriptome assembly from RNA-Seq data without a reference genome. Nat Biotechnol 29: 644–652. 10.1038/nbt.1883 21572440 PMC3571712

[96] Haas BJ, Papanicolaou A, Yassour M, Grabherr M, Blood PD, et al (2013) De novo transcript sequence reconstruction from RNA-seq using the Trinity platform for reference generation and analysis. Nat Protoc 8: 1494–1512. 10.1038/nprot.2013.084 23845962 PMC3875132

[97] Buchfink B, Xie C, Huson DH (2014) Fast and sensitive protein alignment using DIAMOND. Nat Methods 12: 59–60. 10.1038/nmeth.3176 25402007

[98] Huson DH, Beier S, Flade I, Górska A, El-Hadidi M, et al (2016) MEGAN community edition—interactive exploration and analysis of large-scale microbiome sequencing data. PLoS Comput Biol 12: e1004957 10.1371/journal.pcbi.1004957 27327495 PMC4915700

[99] Bao W, Kojima KK, Kohany O (2015) Repbase Update, a database of repetitive elements in eukaryotic genomes. Mob DNA 6: 11 10.1186/s13100-015-0041-9 26045719 PMC4455052

[100] Shi M, Lin X-D, Tian J-H, Chen L-J, Chen X, et al (2016) Redefining the invertebrate RNA virosphere. Nature 540: 539–543. 10.1038/nature20167 27880757

[101] Hennebert E, Leroy B, Wattiez R, Ladurner P (2015) An integrated transcriptomic and proteomic analysis of sea star epidermal secretions identifies proteins involved in defense and adhesion. J Proteomics 128: 83–91. 10.1016/j.jprot.2015.07.002 26171724

[102] Holmes E (2009) The Evolution and Emergence of RNA Viruses. Oxford University Press.

[103] Shi M, Lin X-D, Chen X, Tian J-H, Chen L-J, et al (2018) The evolutionary history of vertebrate RNA viruses. Nature 556: 197–202. 10.1038/s41586-018-0012-7 29618816

[104] Li C-X, Shi M, Tian J-H, Lin X-D, Kang Y-J, et al (2015) Unprecedented genomic diversity of RNA viruses in arthropods reveals the ancestry of negative-sense RNA viruses. Elife 4: 4–6. 10.7554/eLife.05378 25633976 PMC4384744

[105] Jousset F-X, Plus N, Croizier G, Thomas M (1972) Existence chez *Drosophila* de deux groupes de picornaviruaea de propietes serologiques et biologiques differentes. Comptes Rendus l’Académie des Sci 275: 3043–3046. 4631976

[106] Brun P, Plus N (1980) The viruses of *Drosophila* In: Ashburner M, Wright TRF, editors. The Genetics and Biology of Drosophila. Academic Press, London pp. 625–702.

[107] Reinganum C, O’Loughlin G, Hogan T (1970) A nonoccluded virus of the field crickets *Teleogryllus aceanicus* and *T*. *commodus* (Orthoptera: Gryllidae). J Invertebr Pathol 16: 220–314.

[108] Kircher M, Sawyer S, Meyer M (2012) Double indexing overcomes inaccuracies in multiplex sequencing on the Illumina platform. Nucleic Acids Res 40: 1–8. 22021376 10.1093/nar/gkr771 PMC3245947

[109] Ballenghien M, Faivre N, Galtier N (2017) Patterns of cross-contamination in a multispecies population genomic project: detection, quantification, impact, and solutions. BMC Biol 15: 25 10.1186/s12915-017-0366-6 28356154 PMC5370491

[110] Katzourakis A, Gifford RJ (2010) Endogenous viral elements in animal genomes. PLoS Genet 6: e1001191 10.1371/journal.pgen.1001191 21124940 PMC2987831

[111] Rogato A, Richard H, Sarazin A, Voss B, Cheminant Navarro S, et al (2014) The diversity of small non-coding RNAs in the diatom Phaeodactylum tricornutum. BMC Genomics 15: 698 10.1186/1471-2164-15-698 25142710 PMC4247016

[112] Montgomery TA, Howell MD, Cuperus JT, Li D, Hansen JE, et al (2008) Specificity of ARGONAUTE7-miR390 interaction and dual functionality in TAS3 Trans-acting siRNA formation. Cell 133: 128–141. 10.1016/j.cell.2008.02.033 18342362

[113] Garcia-Ruiz H, Carbonell A, Hoyer JS, Fahlgren N, Gilbert KB, et al (2015) Roles and programming of *Arabidopsis* ARGONAUTE proteins during Turnip Mosaic Virus infection. PLoS Pathog 11: e1004755 10.1371/journal.ppat.1004755 25806948 PMC4373807

[114] Campo S, Gilbert KB, Carrington JC (2016) Small RNA-based antiviral defense in the phytopathogenic fungus *Colletotrichum higginsianum*. PLOS Pathog 12: e1005640 10.1371/journal.ppat.1005640 27253323 PMC4890784

[115] Donaire L, Ayllón MA (2017) Deep sequencing of mycovirus-derived small RNAs from *Botrytis* species. Mol Plant Pathol 18: 1127–1137. 10.1111/mpp.12466 27578449 PMC6638239

[116] Cock JM, Sterck L, Rouzé P, Scornet D, Allen AE, et al (2010) The *Ectocarpus genome* and the independent evolution of multicellularity in brown algae. Nature 465: 617–621. 10.1038/nature09016 20520714

[117] Tarver JE, Cormier A, Pinzon N, Taylor RS, Carre W, et al (2015) microRNAs and the evolution of complex multicellularity: identification of a large, diverse complement of microRNAs in the brown alga *Ectocarpus*. Nucleic Acids Res: 1–15. 10.1093/nar/gkv578 26101255 PMC4513859

[118] Cock JM, Liu F, Duan D, Bourdareau S, Lipinska AP, et al (2017) Rapid evolution of microRNA loci in the brown algae. Genome Biol Evol 9: 740–749. 10.1093/gbe/evx038 28338896 PMC5381526

[119] Zografidis A, Van Nieuwerburgh F, Kolliopoulou A, Apostolou-Karampelis K, Head SR, et al (2015) Viral small RNA analysis of *Bombyx mori* larval midgut during persistent and pathogenic cytoplasmic polyhedrosis virus infection. J Virol 89: JVI.01695-15. 10.1128/JVI.01695-15 26339065 PMC4645660

[120] Félix M-A, Ashe A, Piffaretti J, Wu G, Nuez I, et al (2011) Natural and experimental infection of *Caenorhabditis* Nematodes by novel viruses related to nodaviruses. PLoS Biol 9: e1000586 10.1371/journal.pbio.1000586 21283608 PMC3026760

[121] Chejanovsky N, Ophir R, Schwager MS, Slabezki Y, Grossman S, et al (2014) Characterization of viral siRNA populations in honey bee colony collapse disorder. Virology 454–455: 176–183. 10.1016/j.virol.2014.02.012 24725944

[122] Samuel C (2012) ADARs, viruses and innate immunity. Curr Opin Microbiol Immunol 353: 163–195. 10.1007/82_2011_148 PMC3867276 21809195

[123] Ameres S, Horwich M, Hung J-H, Xu J, Ghildiyal M, et al (2010) Target RNA–directed trimming and tailing of small silencing RNAs. Science (80-) 328: 1534–1539. 10.1126/science.1187058 PMC2902985 20558712

[124] Kozomara A, Griffiths-Jones S (2014) MiRBase: Annotating high confidence microRNAs using deep sequencing data. Nucleic Acids Res 42: 68–73. 10.1093/nar/gkt1181 24275495 PMC3965103

[125] Bronkhorst AW, Miesen P, van Rij RP (2013) Small RNAs tackle large viruses: RNA interference-based antiviral defense against DNA viruses in insects. Fly (Austin) 7: 216–223. 10.4161/fly.25708 23974177 PMC3896492

[126] Rajeswaren R, Pooggin M (2012) Role of virus-derived small RNAs in plant antiviral defense: insights from DNA viruses In: Sunkar R, editor. MicroRNAs in Plant Development and Stress Response. Springer International Publishing pp. 261–289.

[127] Lee HC, Gu W, Shirayama M, Youngman E, Conte D, et al (2012) C. elegans piRNAs mediate the genome-wide surveillance of germline transcripts. Cell 150: 78–87. 10.1016/j.cell.2012.06.016 22738724 PMC3410639

[128] Das PP, Bagijn MP, Goldstein LD, Woolford JR, Lehrbach NJ, et al (2008) Piwi and piRNAs act upstream of an endogenous siRNA pathway to suppress Tc3 transposon mobility in the *Caenorhabditis elegans* germline. Mol Cell 31: 79–90. 10.1016/j.molcel.2008.06.003 18571451 PMC3353317

[129] Deng W, Lin H (2002) miwi, a murine homolog of piwi, encodes a cytoplasmic protein essential for spermatogenesis. Dev Cell 2: 819–830. 12062093 10.1016/s1534-5807(02)00165-x

[130] Kuramochi-Miyagawa S (2004) Mili, a mammalian member of piwi family gene, is essential for spermatogenesis. Development 131: 839–849. 10.1242/dev.00973 14736746

[131] Czech B, Malone CD, Zhou R, Stark A, Schlingeheyde C, et al (2008) An endogenous small interfering RNA pathway in *Drosophila*. Nature 453: 798–802. 10.1038/nature07007 18463631 PMC2895258

[132] Gao Z, Wang M, Blair D, Zheng Y, Dou Y (2014) Phylogenetic analysis of the endoribonuclease Dicer family. PLoS One 9: e95350 10.1371/journal.pone.0095350 24748168 PMC3991619

[133] Huang Y, Kendall T, Forsythe ES, Dorantes-Acosta A, Li S, et al (2015) Ancient origin and recent innovations of RNA polymerase IV and V. Mol Biol Evol 32: 1788–1799. 10.1093/molbev/msv060 25767205 PMC4476159

[134] Bollmann SR, Fang Y, Press CM, Tyler BM, Grünwald NJ (2016) Diverse evolutionary trajectories for small RNA biogenesis genes in the oomycete genus *Phytophthora*. Front Plant Sci 7: 1–15. 27014308 10.3389/fpls.2016.00284 PMC4791657

[135] Liew YJ, Ryu T, Aranda M, Ravasi T (2016) miRNA repertoires of demosponges *Stylissa carteri* and *Xestospongia testudinaria*. PLoS One 11: e0149080 10.1371/journal.pone.0149080 26871907 PMC4752309

[136] Rosani U, Pallavicini A, Venier P (2016) The miRNA biogenesis in marine bivalves. PeerJ 4: e1763 10.7717/peerj.1763 26989613 PMC4793324

[137] Coruh C, Cho SH, Shahid S, Liu Q, Wierzbicki A, et al (2015) Comprehensive annotation of *Physcomitrella patens* small RNA loci reveals that the heterochromatic short interfering RNA pathway Is largely conserved in land plants. Plant Cell 27: tpc.15.00228-. 10.1105/tpc.15.00228 26209555 PMC4568501

[138] Shoguchi E, Shinzato C, Kawashima T, Gyoja F, Mungpakdee S, et al (2013) Draft assembly of the *Symbiodinium minutum* nuclear genome reveals dinoflagellate gene structure. Curr Biol 23: 1399–1408. 10.1016/j.cub.2013.05.062 23850284

[139] Moran Y, Praher D, Fredman D, Technau U (2013) The evolution of MicroRNA pathway protein components in Cnidaria. Mol Biol Evol 30: 2541–2552. 10.1093/molbev/mst159 24030553 PMC3840309

[140] Hu Y, Stenlid J, Elfstrand M, Olson A (2013) Evolution of RNA interference proteins Dicer and Argonaute in Basidiomycota. Mycologia 105: 1489–1498. 10.3852/13-171 23928424

[141] Buck AH, Blaxter M (2013) Functional diversification of Argonautes in nematodes: an expanding universe. Biochem Soc Trans 41: 881–886. 10.1042/BST20130086 23863149 PMC3782831

[142] Singh RK, Gase K, Baldwin IT, Pandey SP (2015) Molecular evolution and diversification of the Argonaute family of proteins in plants. BMC Plant Biol 15: 23 10.1186/s12870-014-0364-6 25626325 PMC4318128

[143] Takeuchi T, Koyanagi R, Gyoja F, Kanda M, Hisata K, et al (2016) Bivalve-specific gene expansion in the pearl oyster genome: implications of adaptation to a sessile lifestyle. Zool Lett 2: 3 10.1186/s40851-016-0039-2 26900483 PMC4759782

[144] Schwach F, Vaistij F, Jones L, Baulcombe D (2005) An RNA-dependent RNA polymerase prevents meristem invasion by Potato Virus X and is required for the activity but not the production of a systemic silencing signal. Plant Physiol 138: 1842–1852. 10.1104/pp.105.063537 16040651 PMC1183376

[145] Schott DH, Cureton DK, Whelan SP, Hunter CP (2005) An antiviral role for the RNA interference machinery in Caenorhabditis elegans. Proc Natl Acad Sci 102: 18420–18424. 10.1073/pnas.0507123102 16339901 PMC1317933

[146] Wilkins C, Dishongh R, Moore SC, Whitt M a, Chow M, et al (2005) RNA interference is an antiviral defence mechanism in Caenorhabditis elegans. Nature 436: 1044–1047. 10.1038/nature03957 16107852

[147] Denker E, Manuel M, Leclère L, Le Guyader H, Rabet N (2008) Ordered progression of nematogenesis from stem cells through differentiation stages in the tentacle bulb of *Clytia hemisphaerica* (Hydrozoa, Cnidaria). Dev Biol 315: 99–113. 10.1016/j.ydbio.2007.12.023 18234172

[148] MacRae IJ (2006) Structural basis for double-stranded RNA processing by dicer. Science (80-) 311: 195–198. 10.1126/science.1121638 16410517

[149] Webster CL, Waldron FM, Robertson S, Crowson D, Ferrari G, et al (2015) The discovery, distribution, and evolution of viruses associated with *Drosophila melanogaster*. PLOS Biol 13: e1002210 10.1371/journal.pbio.1002210 26172158 PMC4501690

[150] Palatini U, Miesen P, Carballar-Lejarazu R, Ometto L, Tu Z, et al (2017) Comparative genomics shows that viral integrations are abundant and express. BMC Genomics 18: 1–15. 28676109 10.1186/s12864-017-3903-3 PMC5497376

[151] van Mierlo JT, Bronkhorst AW, Overheul GJ, Sadanandan S a, Ekström J-O, et al (2012) Convergent evolution of argonaute-2 slicer antagonism in two distinct insect RNA viruses. PLoS Pathog 8: e1002872 10.1371/journal.ppat.1002872 22916019 PMC3420963

[152] Hamilton A, Voinnet O, Chappell L, Baulcombe D (2002) Two classes of short interfering RNA in RNA silencing. Embo J 21: 4671–4679. 10.1093/emboj/cdf464 12198169 PMC125409

[153] Zhang X, Segers GC, Sun Q, Deng F, Nuss DL (2008) Characterization of hypovirus-derived small RNAs generated in the chestnut blight fungus by an inducible DCL-2-dependent pathway. J Virol 82: 2613–2619. 10.1128/JVI.02324-07 18199652 PMC2258980

[154] Drinnenberg IA, Fink GR, Bartel DP (2011) Compatibility with killer explains the rise of RNAi-deficient fungi. Science (80-) 333: 1592–1592. 10.1126/science.1209575 21921191 PMC3790311

[155] Arensburger P, Hice RH, Wright JA, Craig NL, Atkinson PW (2011) The mosquito *Aedes aegypti* has a large genome size and high transposable element load but contains a low proportion of transposon-specific piRNAs. BMC Genomics 12: 606 10.1186/1471-2164-12-606 22171608 PMC3259105

[156] Handler D, Meixner K, Pizka M, Lauss K, Schmied C, et al (2013) The genetic makeup of the *Drosophila* piRNA pathway. Mol Cell 50: 762–777. 10.1016/j.molcel.2013.04.031 23665231 PMC3679447

[157] Benitez AA, Spanko LA, Bouhaddou M, Sachs D, TenOever BR (2015) Engineered mammalian RNAi can elicit antiviral protection that negates the requirement for the interferon response. Cell Rep 13: 1456–1466. 10.1016/j.celrep.2015.10.020 26549455 PMC4654977

[158] Jehn J, Gebert D, Pipilescu F, Stern S, Simon J, et al. (2018) Conserved and ubiquitous expression of piRNAs and PIWI genes in mollusks antedates the origin of somatic PIWI / piRNA expression to the root of bilaterians. bioRXiv.

[159] Olovnikov I, Ryazansky S, Shpiz S, Lavrov S, Abramov Y, et al (2013) De novo piRNA cluster formation in the *Drosophila* germ line triggered by transgenes containing a transcribed transposon fragment. Nucleic Acids Res 41: 5757–5768. 10.1093/nar/gkt310 23620285 PMC3675497

[160] Shpiz S, Ryazansky S, Olovnikov I, Abramov Y, Kalmykova A (2014) Euchromatic transposon insertions trigger production of novel pi- and endo-siRNAs at the target sites in the *Drosophila* germline. PLoS Genet 10: e1004138 10.1371/journal.pgen.1004138 24516406 PMC3916259

[161] Biryukova I, Ye T (2015) Endogenous siRNAs and piRNAs derived from transposable elements and genes in the malaria vector mosquito *Anopheles gambiae*. BMC Genomics 16: 1–17. 25879960 10.1186/s12864-015-1436-1 PMC4423592

[162] Apt KE, Clendennen SK, Powers DA, Grossman AR (1995) The gene family encoding the fucoxanthin chlorophyll proteins from the brown alga *Macrocystis pyrifera*. Mol Gen Genet 246: 455–464. 10.1007/BF00290449 7891659

[163] Floyd R, Rogers A, Lambshead P, Smith C (2005) Nematode-specific PCR primers for the 18S small subunit rRNA gene. Mol Ecol Notes 5: 611–612. 10.1111/j.1471-8286.2005.01009.x

[164] Altschul S, Gish W, Miller W, Myers E, Lipman DJ (1990) Basic local alignment search tool. J Mol Biol 215: 403–410. 10.1016/S0022-2836(05)80360-2 2231712

[165] Notredame C, Higgins DG, Heringa J (2000) T-coffee: a novel method for fast and accurate multiple sequence alignment. J Mol Biol 302: 205–217. 10.1006/jmbi.2000.4042 10964570

[166] Wallace IM, O’Sullivan O, Higgins DG, Notredame C (2006) M-Coffee: Combining multiple sequence alignment methods with T-Coffee. Nucleic Acids Res 34: 1692–1699. 10.1093/nar/gkl091 16556910 PMC1410914

[167] Chenna R, Sugawara H, Koike T, Lopez R, Gibson TJ, et al (2003) Multiple sequence alignment with the Clustal series of programs. Nucleic Acids Res 31: 3497–3500. 10.1093/nar/gkg500 12824352 PMC168907

[168] Thompson JD, Higgins DG, Gibson TJ (1994) ClustalW: improving the sensitivity of progressive multiple sequence aligment through sequence weighting, position specific gap penalties and weight matrix choice. Nucl Acids Res 22: 4673–4680. 7984417 10.1093/nar/22.22.4673 PMC308517

[169] Lee C, Grasso C, Sharlow MF (2002) Multiple sequence alignment using partial order graphs. Bioinformatics 18: 452–464. 11934745 10.1093/bioinformatics/18.3.452

[170] Edgar RC (2004) MUSCLE: Multiple sequence alignment with high accuracy and high throughput. Nucleic Acids Res 32: 1792–1797. 10.1093/nar/gkh340 15034147 PMC390337

[171] Katoh K, Standley DM (2013) MAFFT multiple sequence alignment software version 7: Improvements in performance and usability. Mol Biol Evol 30: 772–780. 10.1093/molbev/mst010 23329690 PMC3603318

[172] Morgenstern B (2004) DIALIGN: Multiple DNA and protein sequence alignment at BiBiServ. Nucleic Acids Res 32: 33–36. 10.1093/nar/gkh373 15215344 PMC441511

[173] Pei J, Sadreyev R, Grishin N V. (2003) PCMA: Fast and accurate multiple sequence alignment based on profile consistency. Bioinformatics 19: 427–428. 10.1093/bioinformatics/btg008 12584134

[174] Do CB, Mahabhashyam MSP, Brudno M, Batzoglou S (2005) ProbCons: Probabilistic consistency-based multiple sequence alignment. Genome Res 15: 330–340. 10.1101/gr.2821705 15687296 PMC546535

[175] Guindon S, Gascuel O (2003) A simple, fast, and accurate algorithm to estimate large phylogenies by maximum likelihood. Syst Biol 52: 696–704. 10.1080/10635150390235520 14530136

[176] Koressaar T, Remm M (2007) Enhancements and modifications of primer design program Primer3. Bioinformatics 23: 1289–1291. 10.1093/bioinformatics/btm091 17379693

[177] Untergasser A, Cutcutache I, Koressaar T, Ye J, Faircloth BC, et al (2012) Primer3-new capabilities and interfaces. Nucleic Acids Res 40: 1–12. 22730293 10.1093/nar/gks596 PMC3424584

[178] Martin M (n.d.) Cutadapt removes adapter sequences from high-throughput sequencing reads. EMBnet.journal 17: 10–12. 10.14806/ej.17.1.200

[179] Langmead B, Salzberg SL (2012) Fast gapped-read alignment with Bowtie 2. Nat Methods 9: 357–359. 10.1038/nmeth.1923 22388286 PMC3322381

[180] Giribet G (2016) New animal phylogeny: future challenges for animal phylogeny in the age of phylogenomics. Org Divers Evol 16: 419–426. 10.1007/s13127-015-0236-4

[181] Mukherjee K, Korithoski B, Kolaczkowski B (2014) Ancient origins of vertebrate-specific innate antiviral immunity. Mol Biol Evol 31: 140–153. 10.1093/molbev/mst184 24109602 PMC3879448

