# Correction: Metagenomic sequencing suggests a diversity of RNA interference-like responses to viruses across multicellular eukaryotes

**DOI:** 10.1371/journal.pgen.1008679

**Published:** 2020-03-02

**Authors:** Fergal M. Waldron, Graham N. Stone, Darren J. Obbard

There are two errors in the article [1] that the authors would like to bring to the readers’ attention.

Firstly, this article reports that miRNAs are likely to be 3' 2'-0-methylated in the cnidarian, *Actinia equina*. The authors fail to note that this has previously been shown for *Nematostella* miRNAs in Supplementary table 1 in Grimson *et al* [2].

In the Results section under the subheading “New virus-like sequences identified by metagenomic sequencing”, there is an error in the penultimate sentence.

Specifically, the authors state with reference to Barns Ness dog whelk orthomyxo-like virus 1, that “the PB2 polymerase subunit falls between Infectious Salmon Anaemia virus and the Influenza/Thogoto virus clade”. This is incorrect, as PB2 should read PB1.

The correct sentence is: These include the Caledonia dog whelk rhabdo-like virus 2 sequence, which is represented by a nucleoprotein that falls between the Rabies/Lyssaviruses and other rhabdoviruses, and Barns Ness dog whelk orthomyxo-like virus 1—for which the PB1 polymerase subunit falls between Infectious Salmon Anaemia virus and the Influenza/Thogoto virus clade (Fig 2; the PA polymerase subunit shows similarity to the Thogoto viruses, but not other Orthomyxoviruses).

The authors do not report, or analyse, the PB2 subunit of Barns Ness dog whelk orthomyxo-like virus 1 in this paper. The sequences for the Barns Ness dog whelk orthomyxo-like virus 1 PB1 and PA subunits are available through GenBank (accession numbers MF190044, MF190045).
