# Supplementary figures and images for: Metagenomic sequencing suggests a diversity of RNA interference-like responses to viruses across multicellular eukaryotes
Source: PLoS Genet. 2018 Jul 30;14(7):e1007533. doi: 10.1371/journal.pgen.1007533 (PMC6085071; doi:10.1371/journal.pgen.1007533)

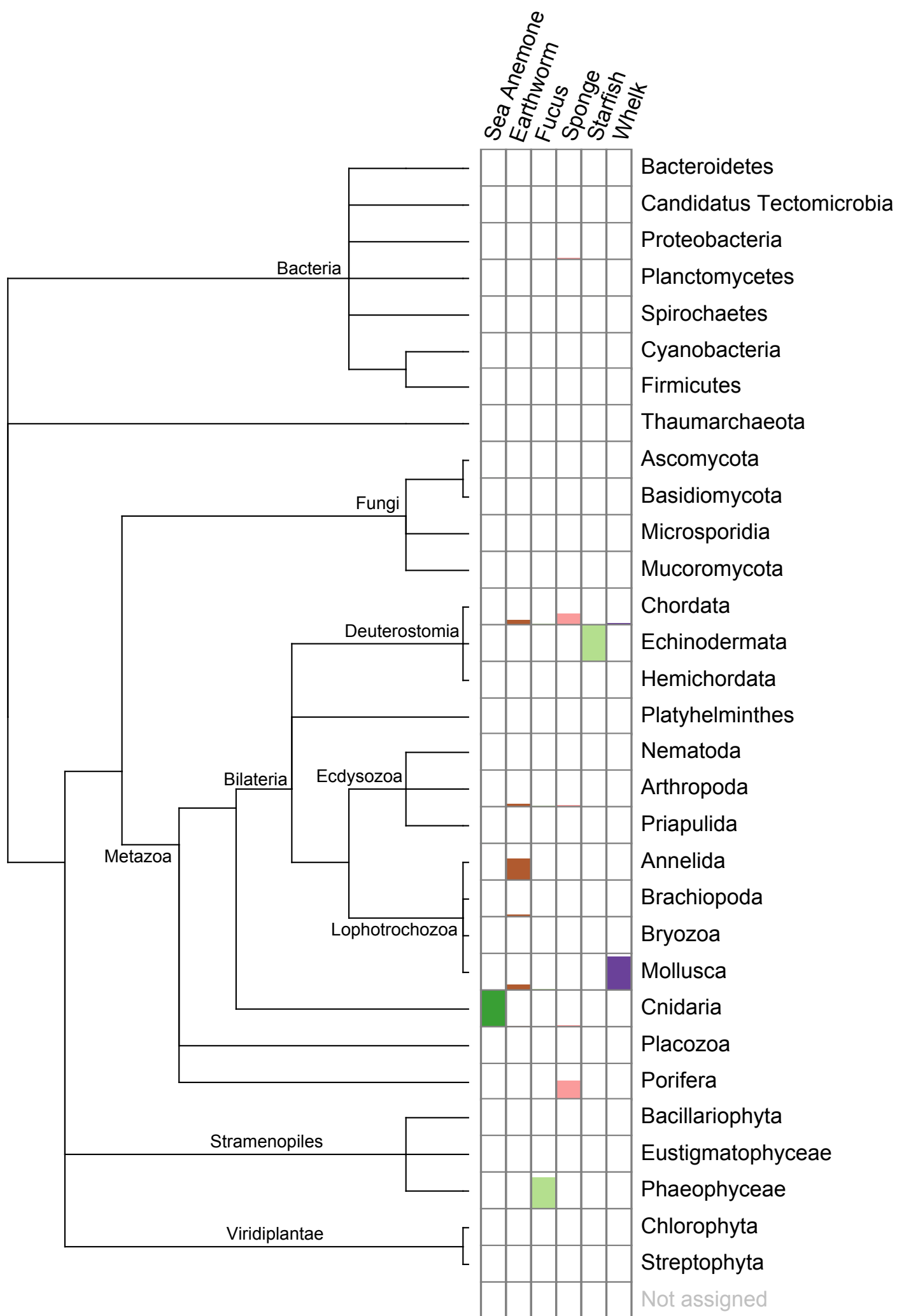

Supplement: S1 Fig — For each of the six organisms, the coloured bars show (on a linear scale), the proportion of all Trinity contigs assigned to each major lineage using Diamond [97] and MEGAN6 [98] with ‘long reads’. (PDF) [file pgen.1007533.s001.pdf]

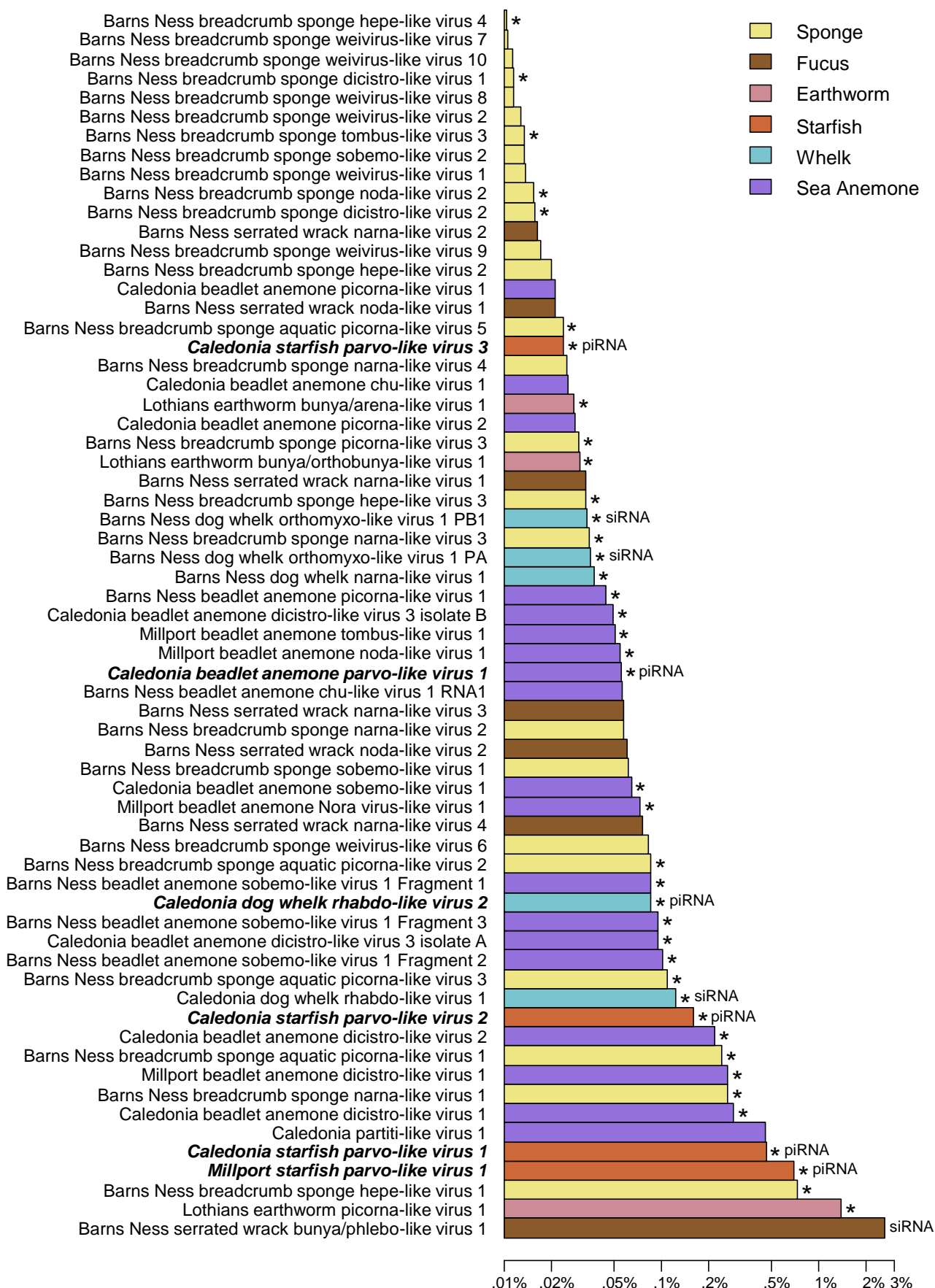

Virus RNAseq reads relative to cytochrome oxidase I

Supplement: S3 Fig — The bar plot shows the relative number of RNAseq reads that mapped to each of virus contigs, as a percentage relative to the read count of host COI reads (both normalised by contig length). Both positive and negative sense reads were included, from library ‘B’ only. Viruses with less than 0.01% of the COI read count were excluded. Contigs marked in bold and italic are thought to be DNA viruses or endogenous viral elements, and contigs marked with an asterisk were surveyed by (RT-PCR). Those contigs that were a source of detectable small RNAs are marked ‘viRNA’ or ‘piRNA’ as appropriate. (PDF) [file pgen.1007533.s003.pdf]

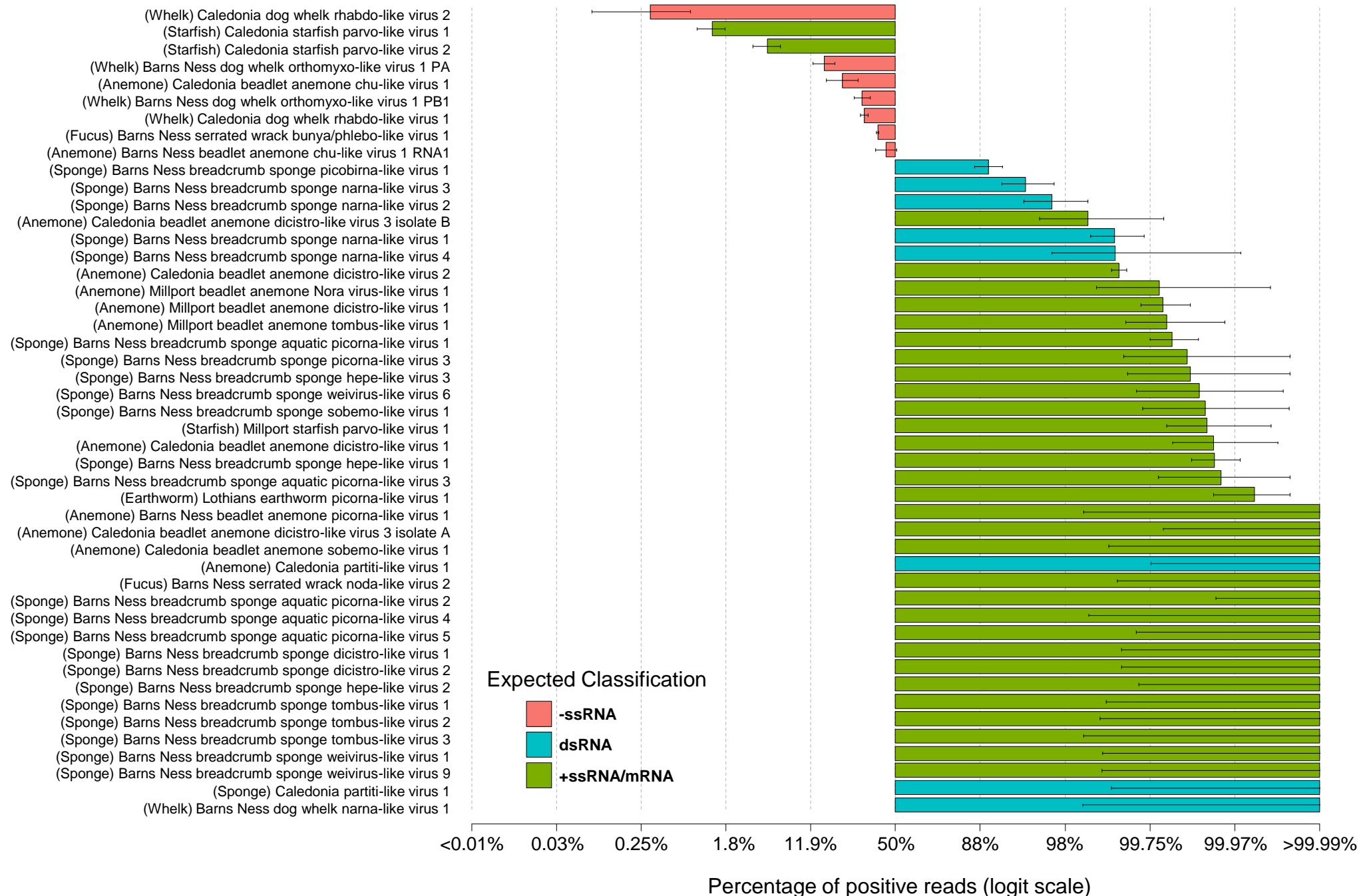

Supplement: S4 Fig — Bars show the proportion of RNA sequencing reads (combined across libraries) derived from the positive (sense) strand of the virus, for all viruses represented by >150 read pairs. Error bars reflect 95% confidence intervals based on a likelihood ratio test, assuming a binomial distribution, and to clearly display ratios close to zero and one the results are plotted on a logit scale. All -ssRNA viruses and dsRNA viruses show strong evidence of replication, as their respective proportions of positive sense reads are >>0% and >>50%. Many of the +ssRNA viruses show evidence of replication, as the proportion of positive reads is <100%. However, the positive to negative strand ratio for replicating +ssRNA reads can be very high, making this a conservative test. Note that two of the putative DNA parvovirus EVEs display negative sense reads, constant with host-driven expression rather than functional mRNA expression. (PDF) [file pgen.1007533.s004.pdf]

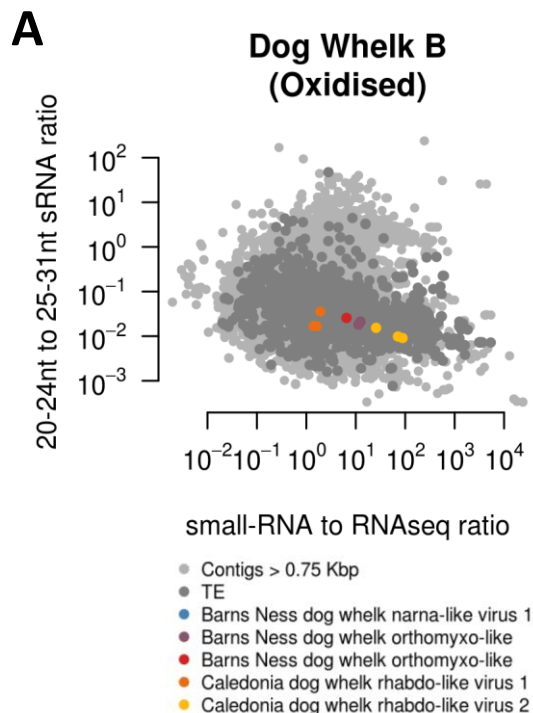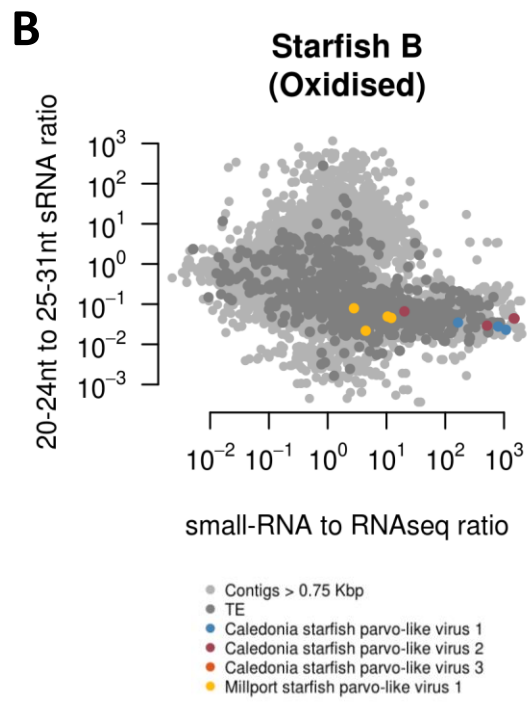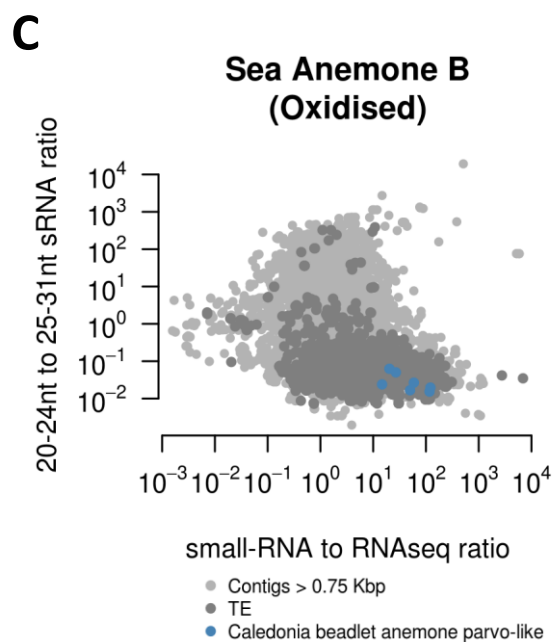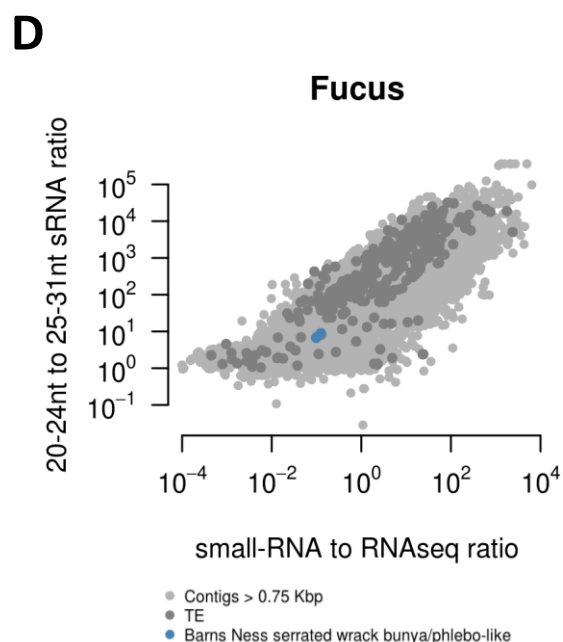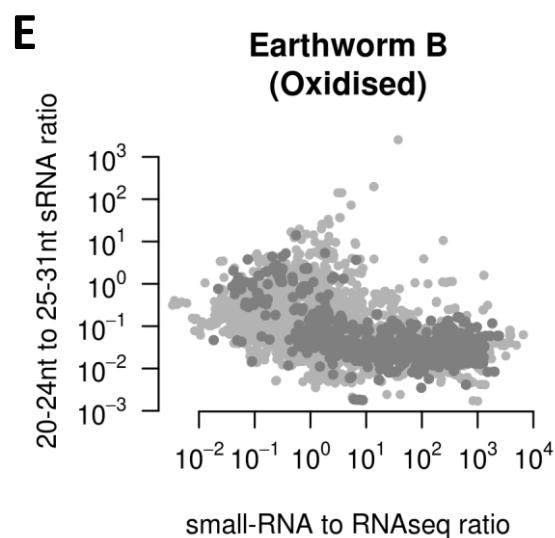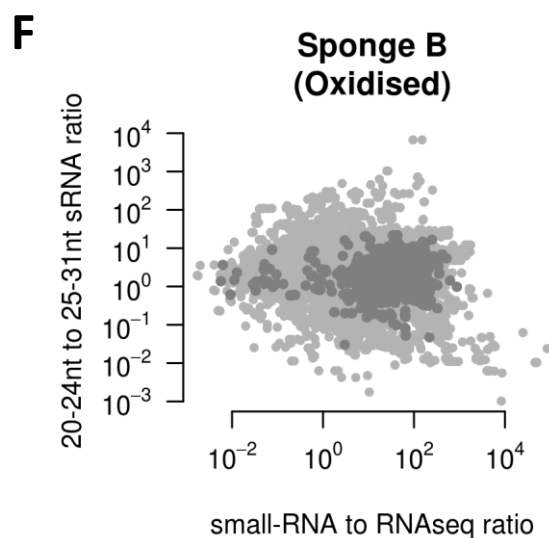

Supplement: S9 Fig — For each metagenomic contig (pale grey) the ratio of sRNAs (20-31nt) to RNAseq reads is shown on the x-axis, and the ratio of 20-24nt sRNAs (expected viRNAs) to 25-31nt sRNAs (expected piRNAs) is shown on the y-axis. Contigs are only included if they are >0.75Kbp in length and produced at least 20 small RNAs; Contigs in dark grey have sequence similarity to known TEs, and contigs in colour correspond to the curated viruses. Based on Drosophila, TEs (dark grey) are expected to appear in the lower right quadrant of each plot, and viruses (colour) in the upper right [see 149]. Only the dog whelk (panel A) and the brown alga (panel D) display sRNAs from RNA virus contigs, although DNA virus-like contigs display piRNA-like small RNAs in the sea anemone (panel C) and the starfish (panel B). No other viruses produced sufficient viRNAs to appear on these figs. All figures (except the brown alga) use data from RNAseq library B and the corresponding oxidised sRNAs (which is enriched for viRNAs over miRNAs), and sRNA counts exclude those mapping to known (miRbase) miRNAs and rRNAs. (PDF) [file pgen.1007533.s009.pdf]
