# Supplementary material for: Metagenomic sequencing suggests a diversity of RNA interference-like responses to viruses across multicellular eukaryotes
Source: PLoS Genet. 2018 Jul 30;14(7):e1007533. doi: 10.1371/journal.pgen.1007533 (PMC6085071; doi:10.1371/journal.pgen.1007533)
Supplement: S2 Fig — Maximum likelihood phylogenetic trees. Support values (approximate likelihood ratio test) and NCBI accession identifiers are provided. Viruses newly identified here are highlighted in red, and unannotated virus-like sequences from publicly-available transcriptome datasets are denoted ‘TSA’. Clade names follow [100,104]. Alignments are provided in S2 Data and Newick format trees in S3 Data. (PDF) [file pgen.1007533.s002.pdf]

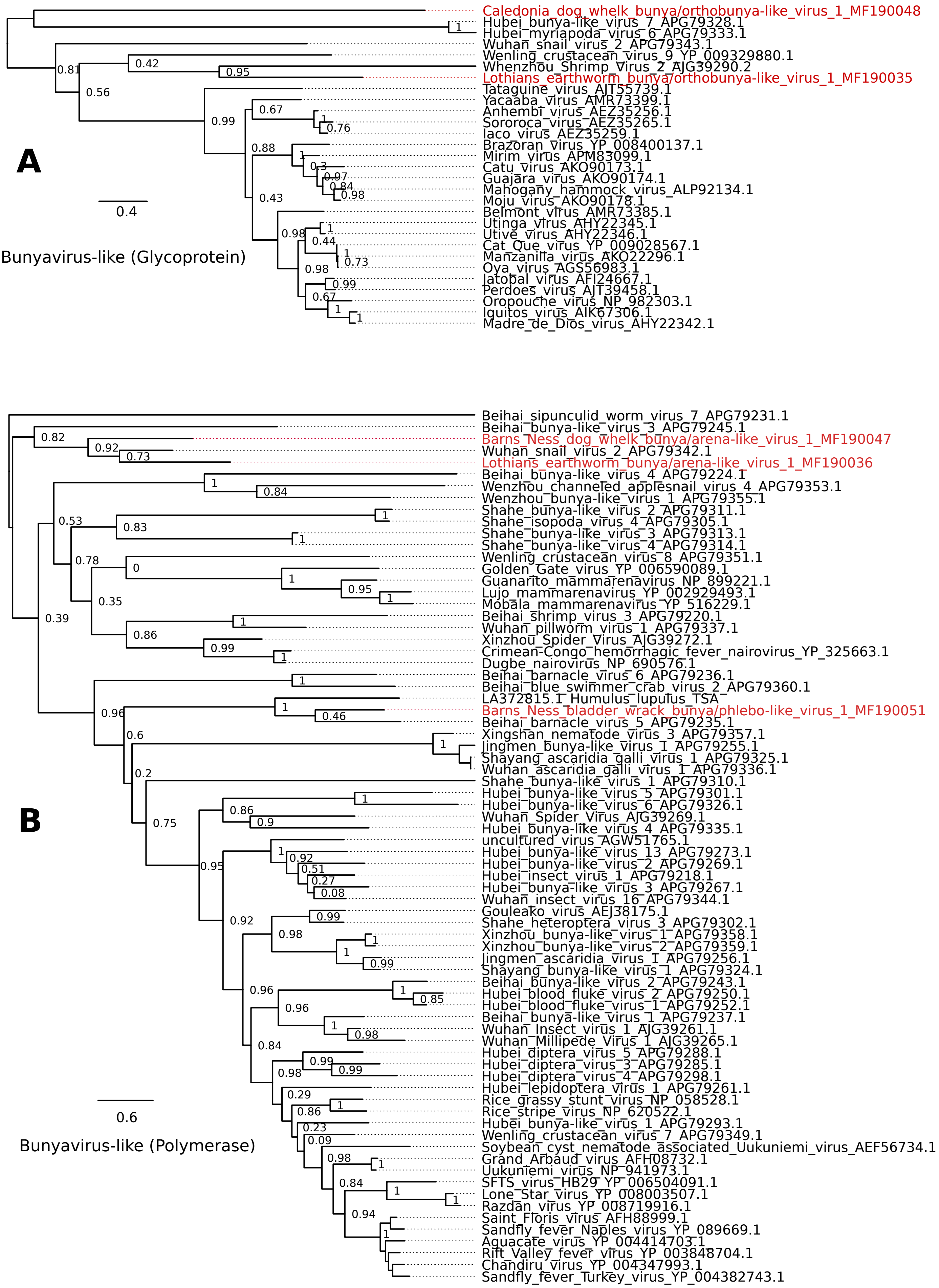
A
0.4
Bunyavirus-like (Glycoprotein)
Caledonia_dog_whelk_bunya/orthobunya-like_virus_1_MF190048
Hubei_bunya-like_virus_7_APG79328.1
Hubei_myriapoda_virus_6_APG79333.1
Wuhan_snail_virus_2_APG79343.1
Wenling_crustacean_virus_9_YP_009329880.1
Whenzhou_Shrimp_Virus_2_AJG39290.2
Lothians_earthworm_bunya/orthobunya-like_virus_1_MF190035
Tataguine_virus_AJT55739.1
Yacaaba_virus_AMR73399.1
Anhembi_virus_AEZ35256.1
Sororoca_virus_AEZ35265.1
Iaco_virus_AEZ35259.1
Brazoran_virus_YP_008400137.1
Mirim_virus_APM83099.1
Catu_virus_AKO90173.1
Guajara_virus_AKO90174.1
Mahogany_hammock_virus_ALP92134.1
Moju_virus_AKO90178.1
Belmont_virus_AMR73385.1
Utinga_virus_AHY22345.1
Utive_virus_AHY22346.1
Cat_Que_virus_YP_009028567.1
Manzanilla_virus_AKO22296.1
Oya_virus_AGS56983.1
Jatobal_virus_AFI24667.1
Perdoes_virus_AJT39458.1
Oropouche_virus_NP_982303.1
Iquitos_virus_AIK67306.1
Madre_de_Dios_virus_AHY22342.1
B
0.6
Bunyavirus-like (Polymerase)
Beihai_sipunculid_worm_virus_7_APG79231.1
Beihai_bunya-like_virus_3_APG79245.1
Barns_Ness_dog_whelk_bunya/arena-like_virus_1_MF190047
Wuhan_snail_virus_2_APG79342.1
Lothians_earthworm_bunya/arena-like_virus_1_MF190036
Beihai_bunya-like_virus_4_APG79224.1
Wenzhou_channeled_applesnail_virus_4_APG79353.1
Wenzhou_bunya-like_virus_1_APG79355.1
Shahe_bunya-like_virus_2_APG79311.1
Shahe_isopoda_virus_4_APG79305.1
Shahe_bunya-like_virus_3_APG79313.1
Shahe_bunya-like_virus_4_APG79314.1
Wenling_crustacean_virus_8_APG79351.1
Golden_Gate_virus_YP_006590089.1
Guanarito_mammarenavirus_NP_899221.1
Lujo_mammarenavirus_YP_002929493.1
Mobala_mammarenavirus_YP_516229.1
Beihai_shrimp_virus_3_APG79220.1
Wuhan_pillworm_virus_1_APG79337.1
Xinzhou_Spider_Virus_AJG39272.1
Crimean-Congo_hemorrhagic_fever_nairovirus_YP_325663.1
Dugbe_nairovirus_NP_690576.1
Beihai_barnacle_virus_6_APG79236.1
Beihai_blue_swimmer_crab_virus_2_APG79360.1
LA372815.1_Humulus_lupulus_TSA
Barns_Ness_bladder_wrack_bunya/phlebo-like_virus_1_MF190051
Beihai_barnacle_virus_5_APG79235.1
Xingshan_nematode_virus_3_APG79357.1
Jingmen_bunya-like_virus_1_APG79255.1
Shayang_ascaridia_galli_virus_1_APG79325.1
Wuhan_ascaridia_galli_virus_1_APG79336.1
Shahe_bunya-like_virus_1_APG79310.1
Hubei_bunya-like_virus_5_APG79301.1
Hubei_bunya-like_virus_6_APG79326.1
Wuhan_Spider_Virus_AJG39269.1
Hubei_bunya-like_virus_4_APG79335.1
uncultured_virus_AGW51765.1
Hubei_bunya-like_virus_13_APG79273.1
Hubei_bunya-like_virus_2_APG79269.1
Hubei_insect_virus_1_APG79218.1
Hubei_bunya-like_virus_3_APG79267.1
Wuhan_insect_virus_16_APG79344.1
Gouleako_virus_AEJ38175.1
Shahe_heteroptera_virus_3_APG79302.1
Xinzhou_bunya-like_virus_1_APG79358.1
Xinzhou_bunya-like_virus_2_APG79359.1
Jingmen_ascaridia_virus_1_APG79256.1
Shayang_bunya-like_virus_1_APG79324.1
Beihai_bunya-like_virus_2_APG79243.1
Hubei_blood_fluke_virus_2_APG79250.1
Hubei_blood_fluke_virus_1_APG79252.1
Beihai_bunya-like_virus_1_APG79237.1
Wuhan_Insect_virus_1_AJG39261.1
Wuhan_Millipede_Virus_1_AJG39265.1
Hubei_diptera_virus_5_APG79288.1
Hubei_diptera_virus_3_APG79285.1
Hubei_diptera_virus_4_APG79298.1
Hubei_lepidoptera_virus_1_APG79261.1
Rice_grassy_stunt_virus_NP_058528.1
Rice_stripe_virus_NP_620522.1
Hubei_bunya-like_virus_1_APG79293.1
Wenling_crustacean_virus_7_APG79349.1
Soybean_cyst_nematode_associated_Uukuniemi_virus_AEF56734.1
Grand_Arbaud_virus_AFH08732.1
Uukuniemi_virus_NP_941973.1
SFTS_virus_HB29_YP_006504091.1
Lone_Star_virus_YP_008003507.1
Razdan_virus_YP_008719916.1
Saint_Floris_virus_AFH88999.1
Sandfly_fever_Naples_virus_YP_089669.1
Aguacate_virus_YP_004414703.1
Rift_Valley_fever_virus_YP_003848704.1
Chandiru_virus_YP_004347993.1
Sandfly_fever_Turkey_virus_YP_004382743.1

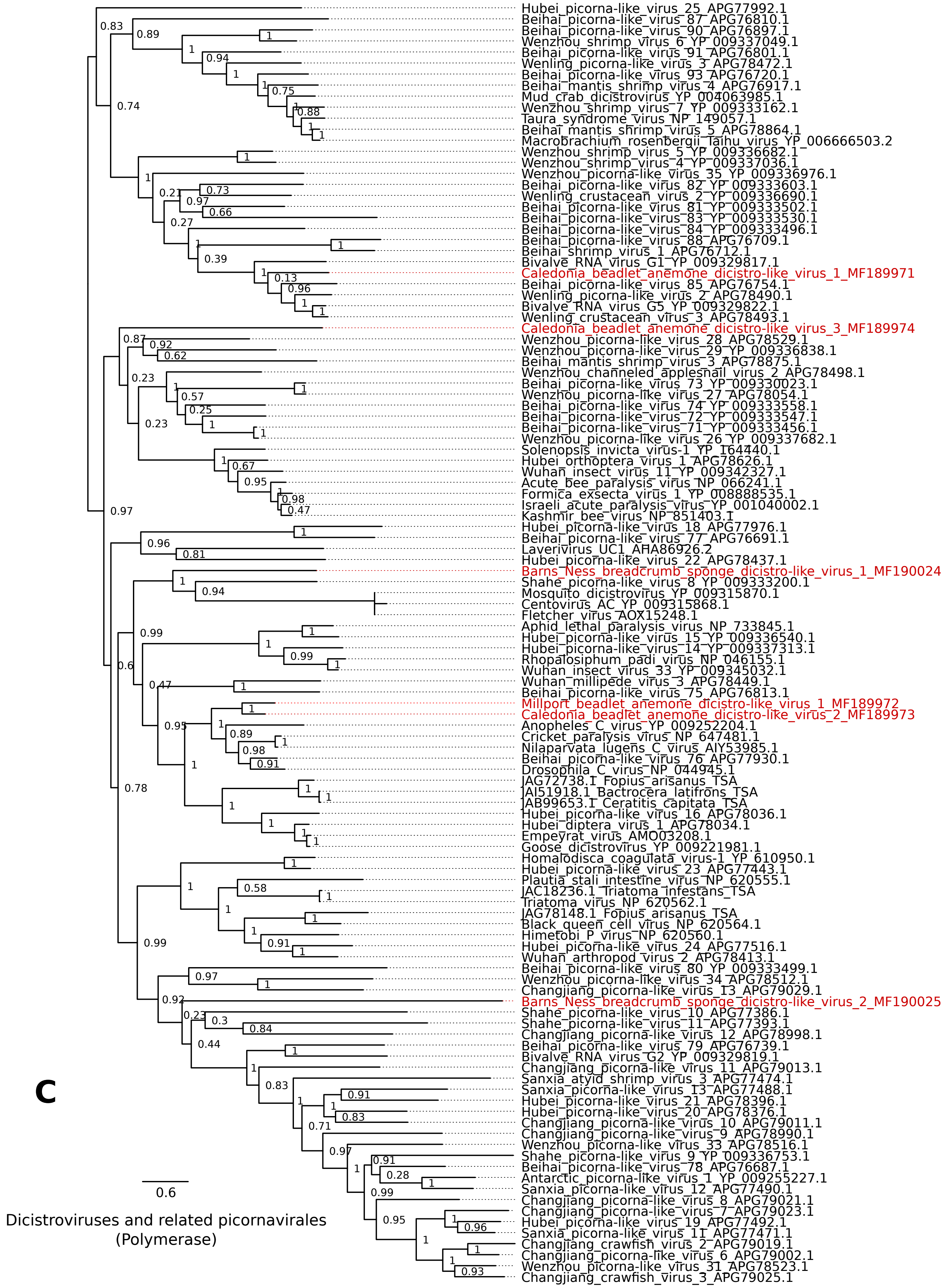
Hubei_picorna-like_virus_25_APG77992.1
Beihai_picorna-like_virus_87_APG76810.1
Beihai_picorna-like_virus_90_APG76897.1
Wenzhou_shrimp_virus_6_YP_009337049.1
Beihai_picorna-like_virus_91_APG76801.1
Wenling_picorna-like_virus_3_APG78472.1
Beihai_picorna-like_virus_93_APG76720.1
Beihai_mantis_shrimp_virus_4_APG76917.1
Mud_crab_dicistrovirus_YP_004063985.1
Wenzhou_shrimp_virus_7_YP_009333162.1
Taura_syndrome_virus_NP_149057.1
Beihai_mantis_shrimp_virus_5_APG78864.1
Macrobrachium_rosenbergii_Taihu_virus_YP_006666503.2
Wenzhou_shrimp_virus_5_YP_009336682.1
Wenzhou_shrimp_virus_4_YP_009337036.1
Wenzhou_picorna-like_virus_35_YP_009336976.1
Beihai_picorna-like_virus_82_YP_009333603.1
Wenling_crustacean_virus_2_YP_009336690.1
Beihai_picorna-like_virus_81_YP_009333502.1
Beihai_picorna-like_virus_83_YP_009333530.1
Beihai_picorna-like_virus_84_YP_009333496.1
Beihai_picorna-like_virus_88_APG76709.1
Beihai_shrimp_virus_1_APG76712.1
Bivalve_RNA_virus_G1_YP_009329817.1
Caledonia_beadlet_anemone_dicistro-like_virus_1_MF189971
Beihai_picorna-like_virus_85_APG76754.1
Wenling_picorna-like_virus_2_APG78490.1
Bivalve_RNA_virus_G5_YP_009329822.1
Wenling_crustacean_virus_3_APG78493.1
Caledonia_beadlet_anemone_dicistro-like_virus_3_MF189974
Wenzhou_picorna-like_virus_28_APG78529.1
Wenzhou_picorna-like_virus_29_YP_009336838.1
Beihai_mantis_shrimp_virus_3_APG78875.1
Wenzhou_channeled_applesnail_virus_2_APG78498.1
Beihai_picorna-like_virus_73_YP_009330023.1
Wenzhou_picorna-like_virus_27_APG78054.1
Beihai_picorna-like_virus_74_YP_009333558.1
Beihai_picorna-like_virus_72_YP_009333547.1
Beihai_picorna-like_virus_71_YP_009333456.1
Wenzhou_picorna-like_virus_26_YP_009337682.1
Solenopsis_invicta_virus-1_YP_164440.1
Hubei_orthoptera_virus_1_APG78626.1
Wuhan_insect_virus_11_YP_009342327.1
Acute_bee_paralysis_virus_NP_066241.1
Formica_exsecta_virus_1_YP_008888535.1
Israeli_acute_paralysis_virus_YP_001040002.1
Kashmir_bee_virus_NP_851403.1
Hubei_picorna-like_virus_18_APG77976.1
Beihai_picorna-like_virus_77_APG76691.1
Laverivirus_UC1_AHA86926.2
Hubei_picorna-like_virus_22_APG78437.1
Barns_Ness_breadcrumb_sponge_dicistro-like_virus_1_MF190024
Shahe_picorna-like_virus_8_YP_009333200.1
Mosquito_dicistrovirus_YP_009315870.1
Centovirus_AC_YP_009315868.1
Fletcher_virus_AOX15248.1
Aphid_lethal_paralysis_virus_NP_733845.1
Hubei_picorna-like_virus_15_YP_009336540.1
Hubei_picorna-like_virus_14_YP_009337313.1
Rhopalosiphum_padi_virus_NP_046155.1
Wuhan_insect_virus_33_YP_009345032.1
Wuhan_millipede_virus_3_APG78449.1
Beihai_picorna-like_virus_75_APG76813.1
Millport_beadlet_anemone_dicistro-like_virus_1_MF189972
Caledonia_beadlet_anemone_dicistro-like_virus_2_MF189973
Anopheles_C_virus_YP_009252204.1
Cricket_paralysis_virus_NP_647481.1
Nilaparvata_lugens_C_virus_AIY53985.1
Beihai_picorna-like_virus_76_APG77930.1
Drosophila_C_virus_NP_044945.1
JAG72738.1_Fopius_arisanus_TSA
JAI51918.1_Bactrocera_latifrons_TSA
JAB99653.1_Ceratitis_capitata_TSA
Hubei_picorna-like_virus_16_APG78036.1
Hubei_diptera_virus_1_APG78034.1
Empeyrat_virus_AMO03208.1
Goose_dicistrovirus_YP_009221981.1
Homalodisca_coagulata_virus-1_YP_610950.1
Hubei_picorna-like_virus_23_APG77443.1
Plautia_stali_intestine_virus_NP_620555.1
JAC18236.1_Triatoma_infestans_TSA
Triatoma_virus_NP_620562.1
JAG78148.1_Fopius_arisanus_TSA
Black_queen_cell_virus_NP_620564.1
Himetobi_P_virus_NP_620560.1
Hubei_picorna-like_virus_24_APG77516.1
Wuhan_arthropod_virus_2_APG78413.1
Beihai_picorna-like_virus_80_YP_009333499.1
Wenzhou_picorna-like_virus_34_APG78512.1
Changjiang_picorna-like_virus_13_APG79029.1
Barns_Ness_breadcrumb_sponge_dicistro-like_virus_2_MF190025
Shahe_picorna-like_virus_10_APG77386.1
Shahe_picorna-like_virus_11_APG77393.1
Changjiang_picorna-like_virus_12_APG78998.1
Beihai_picorna-like_virus_79_APG76739.1
Bivalve_RNA_virus_G2_YP_009329819.1
Changjiang_picorna-like_virus_11_APG79013.1
Sanxia_atyid_shrimp_virus_3_APG77474.1
Sanxia_picorna-like_virus_13_APG77488.1
Hubei_picorna-like_virus_21_APG78396.1
Hubei_picorna-like_virus_20_APG78376.1
Changjiang_picorna-like_virus_10_APG79011.1
Changjiang_picorna-like_virus_9_APG78990.1
Wenzhou_picorna-like_virus_33_APG78516.1
Shahe_picorna-like_virus_9_YP_009336753.1
Beihai_picorna-like_virus_78_APG76687.1
Antarctic_picorna-like_virus_1_YP_009255227.1
Sanxia_picorna-like_virus_12_APG77490.1
Changjiang_picorna-like_virus_8_APG79021.1
Changjiang_picorna-like_virus_7_APG79023.1
Hubei_picorna-like_virus_19_APG77492.1
Sanxia_picorna-like_virus_11_APG77471.1
Changjiang_crawfish_virus_2_APG79019.1
Changjiang_picorna-like_virus_6_APG79002.1
Wenzhou_picorna-like_virus_31_APG78523.1
Changjiang_crawfish_virus_3_APG79025.1
C
0.6
Dicistroviruses and related picornavirales
(Polymerase)

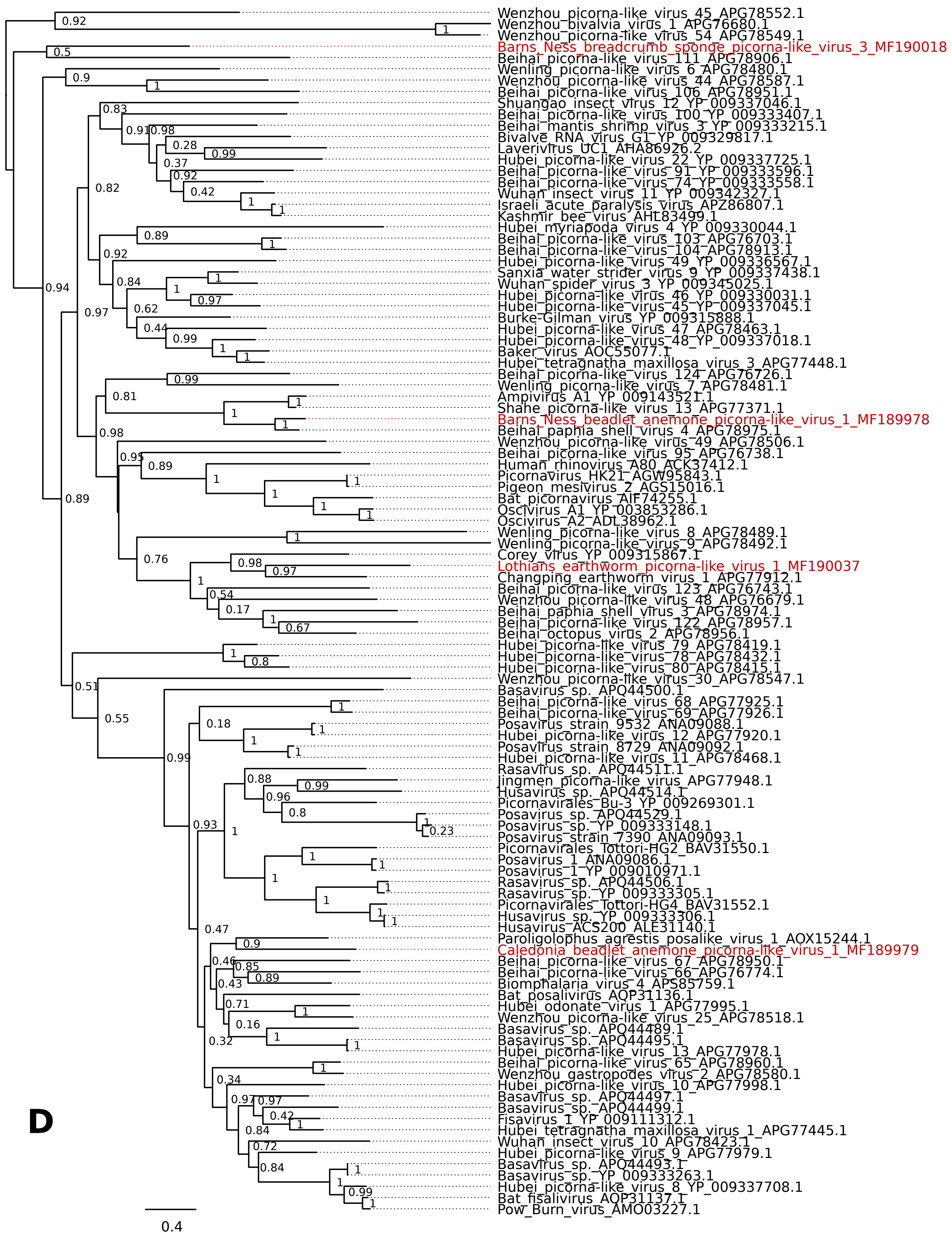

Fisaviruses, Posaviruses, and related picornavirales
(Polymerase)

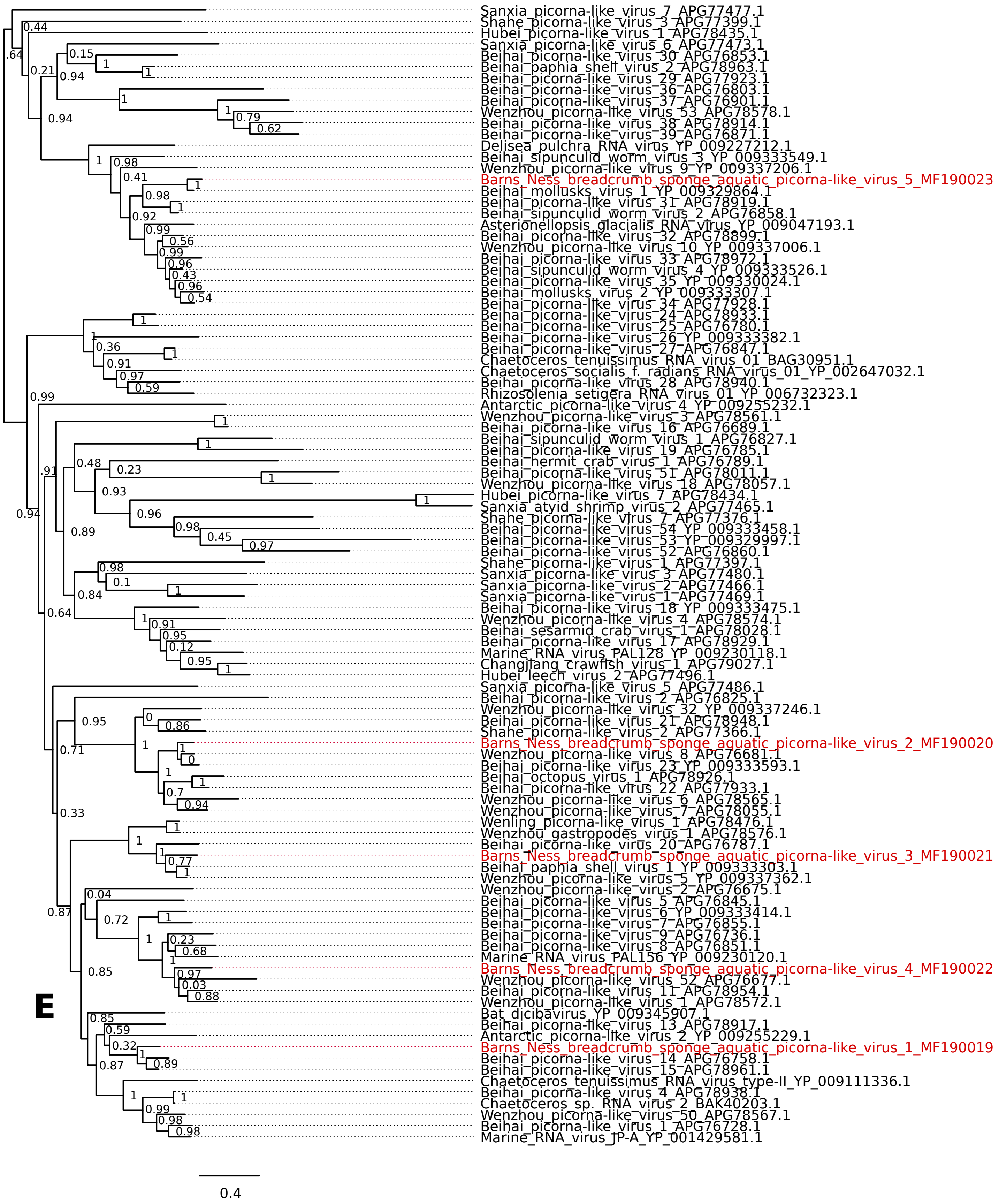

Aquatic picornaviruses and related picornavirales
(Polymerase)

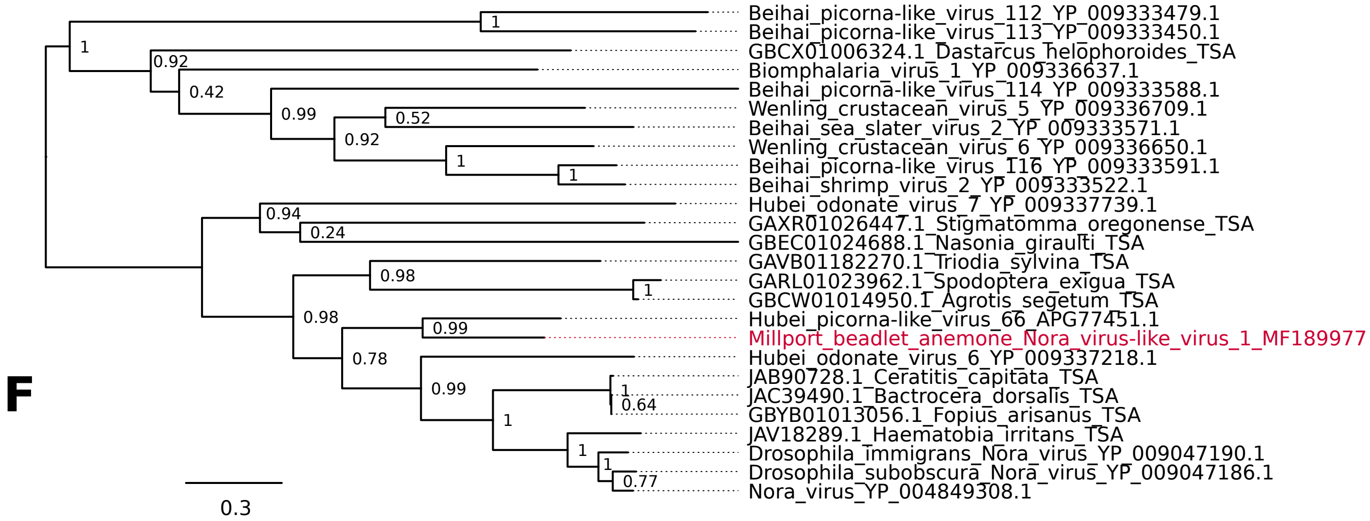

Noraviruses and related picornavirales
(Polymerase)

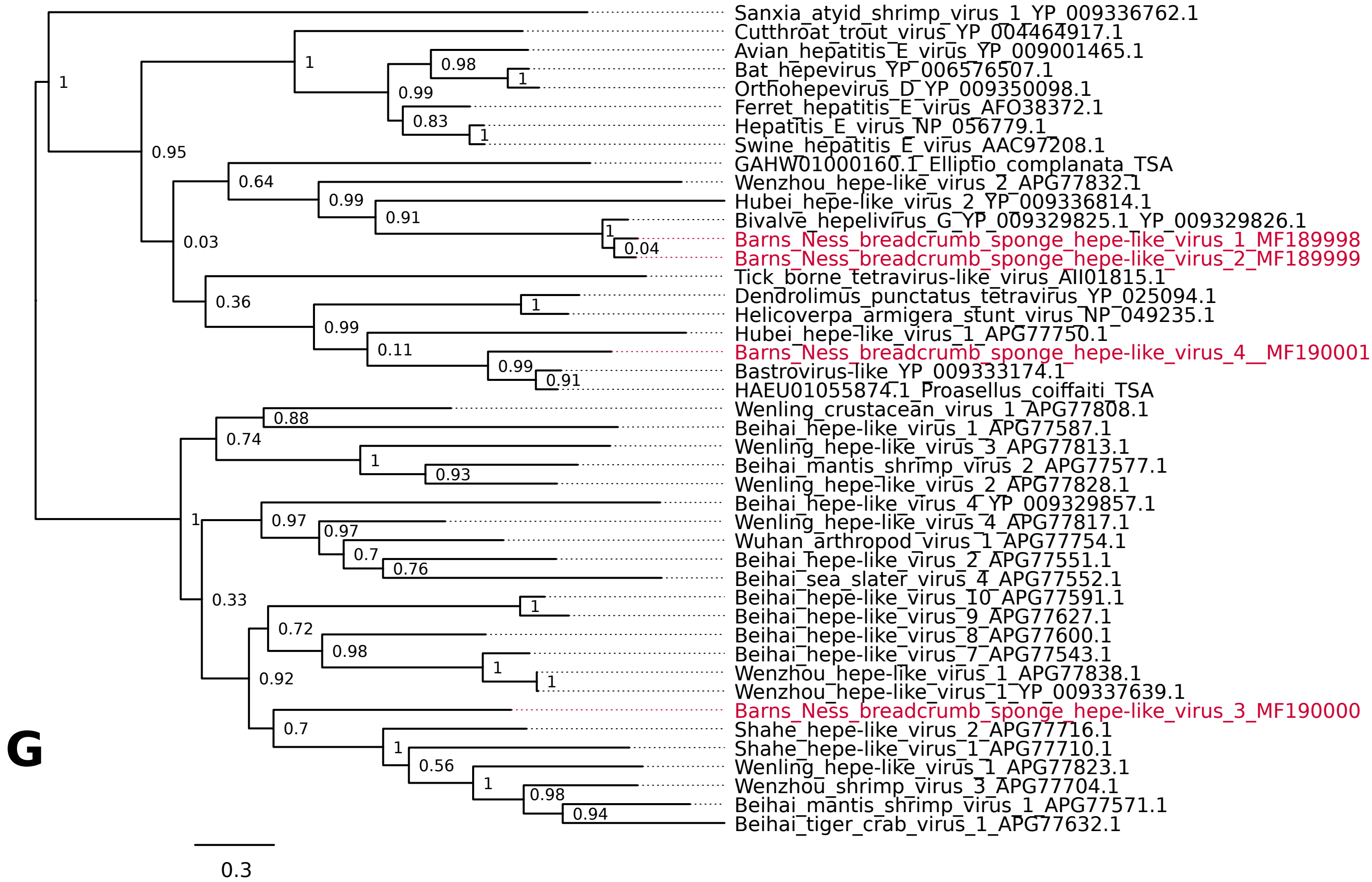

Hepe-like viruses (Polymerase)

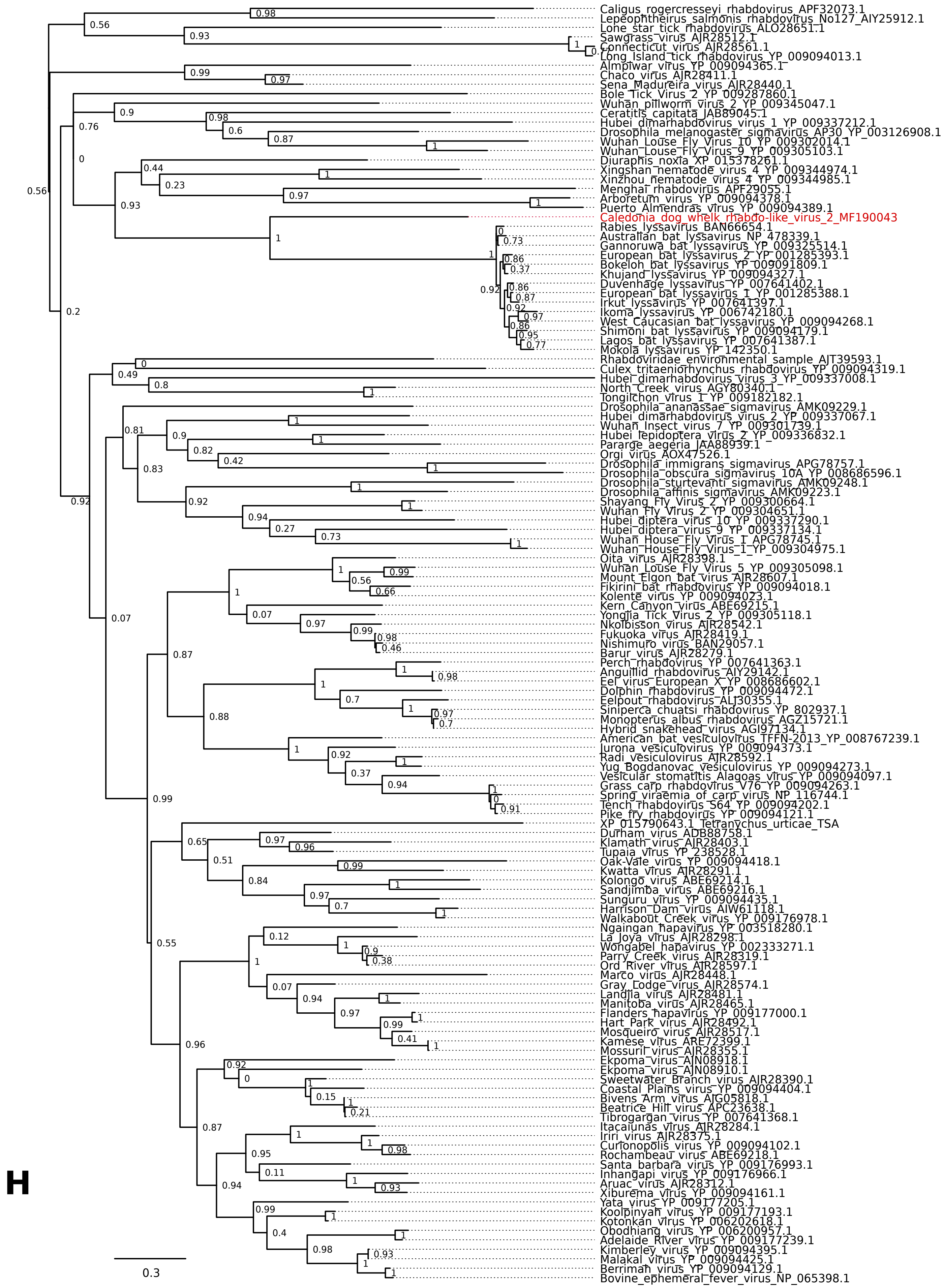

Lyssaviruses and related Rhabdoviruses
(Nucleoprotein)

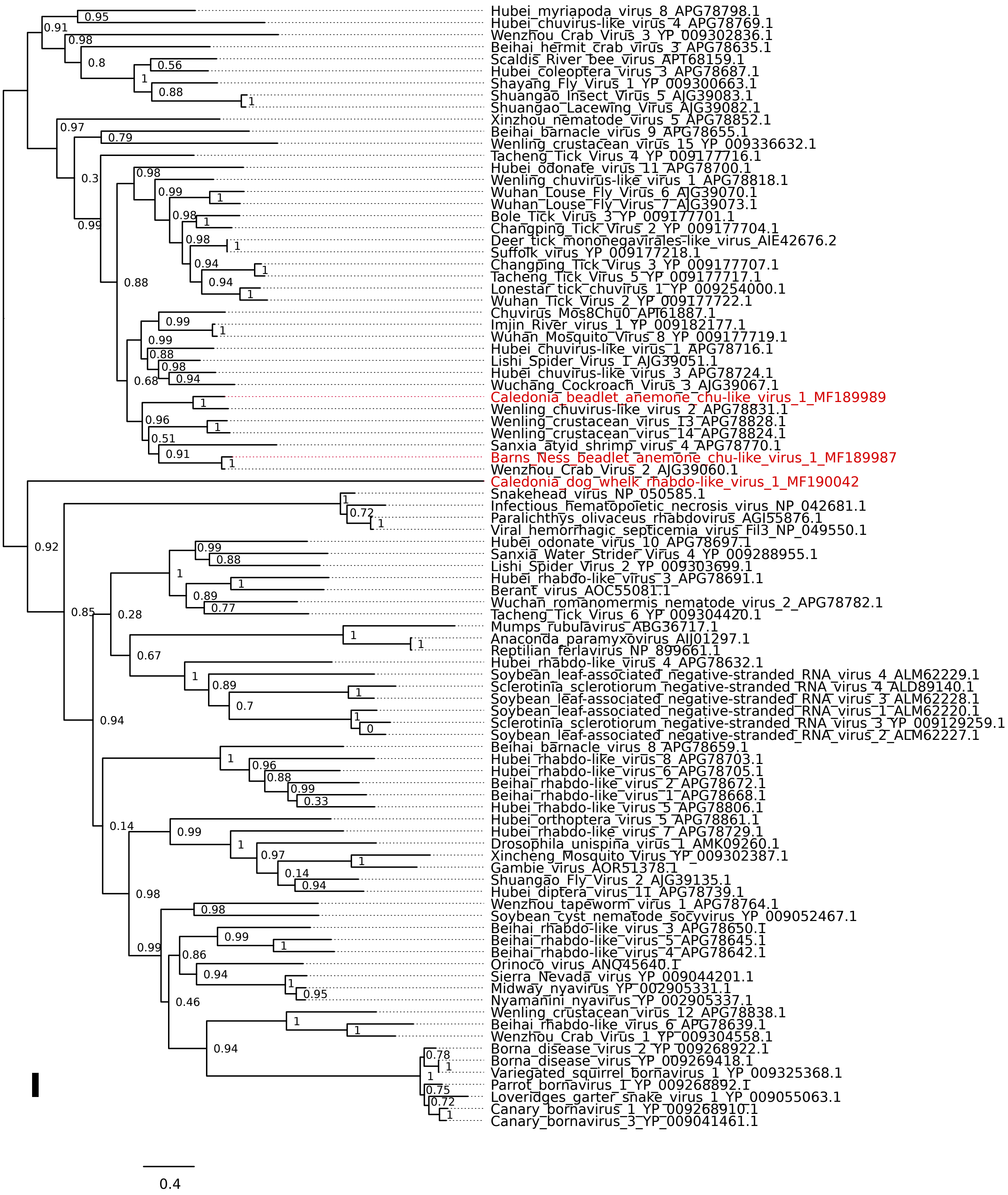

Chuviruses and related Mononegavirales
(Polymerase)

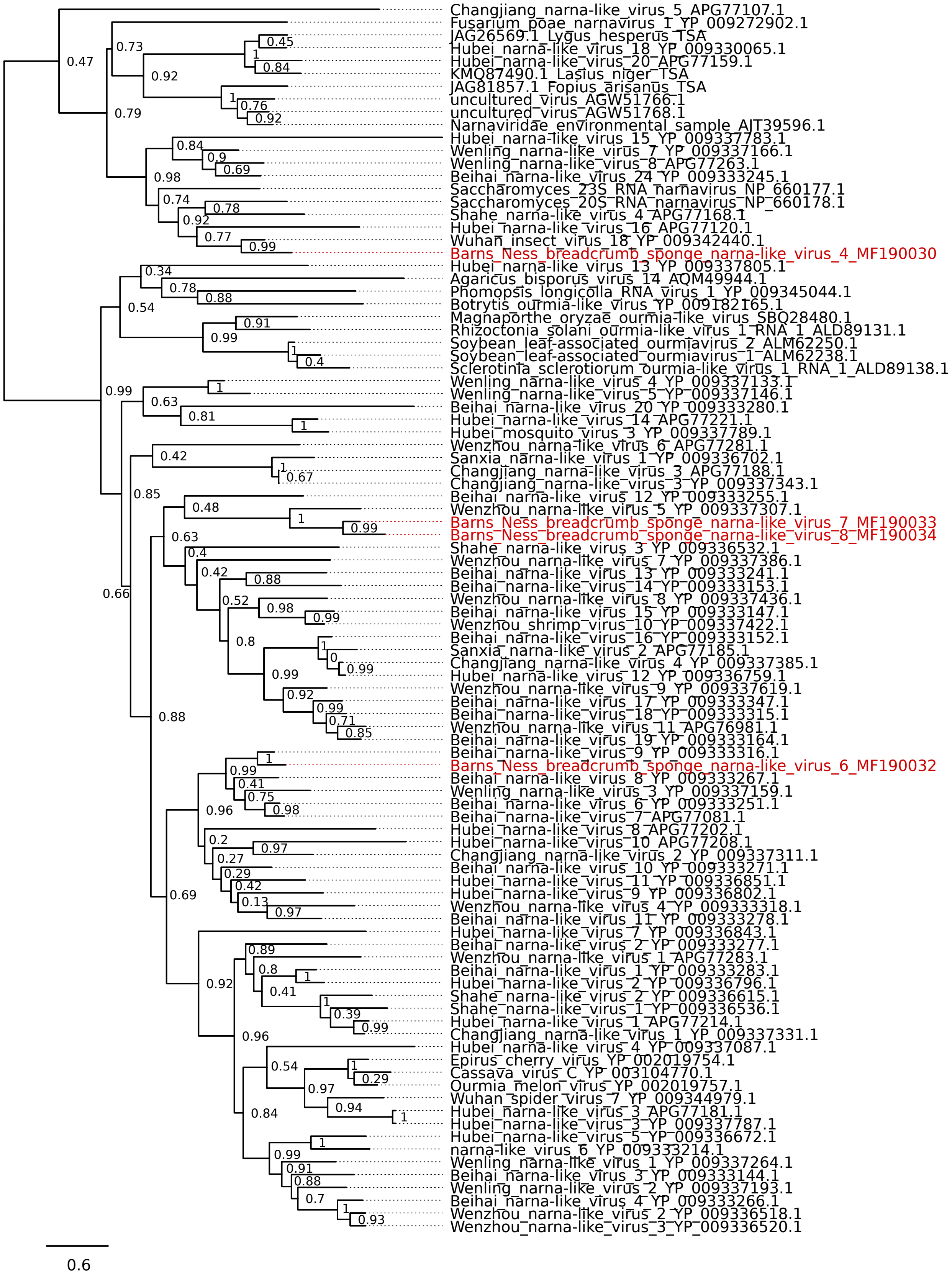

J

Narnaviruses (Polymerase)

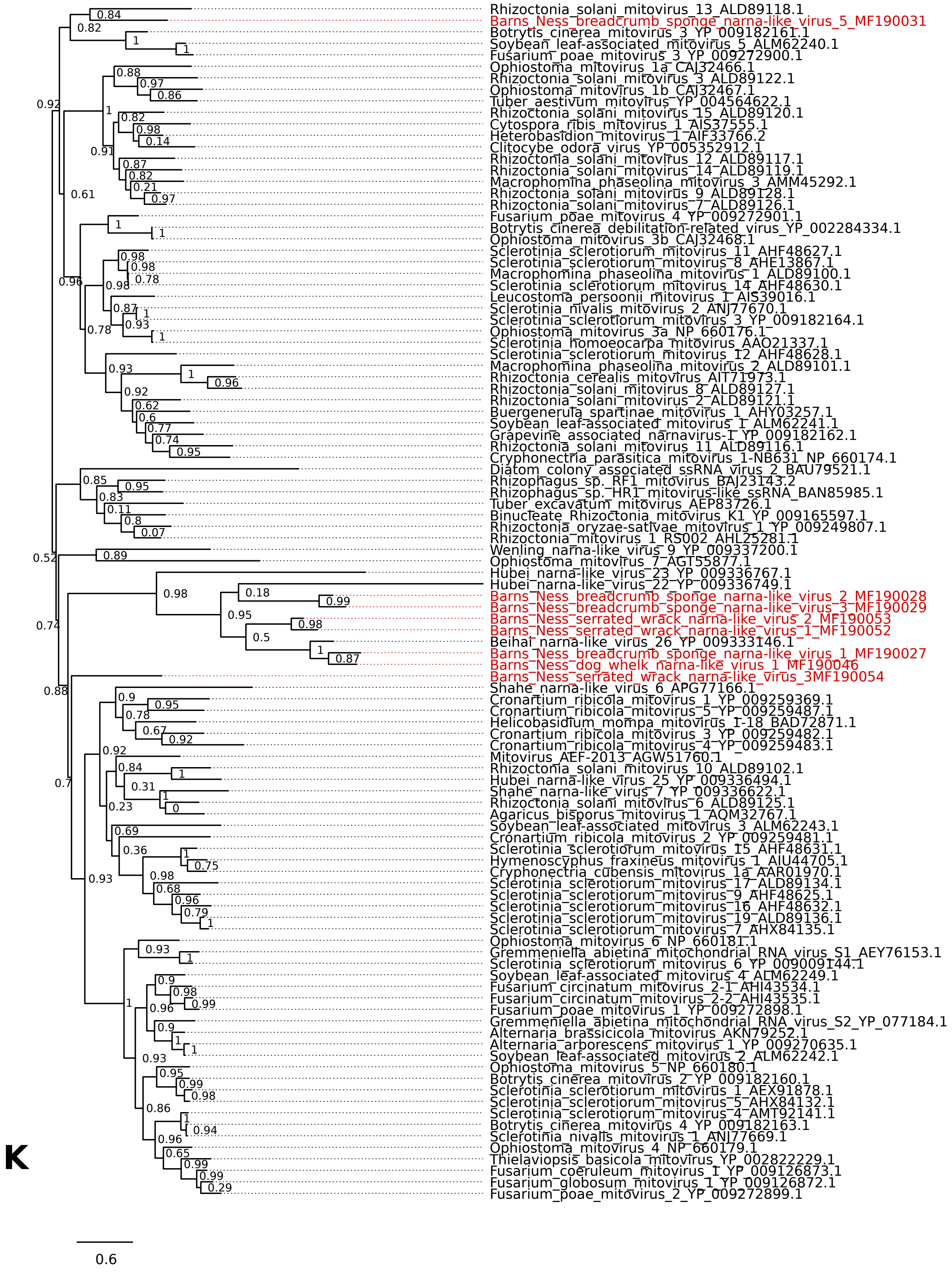

Mitoviruses and related Narnaviruses
(Polymerase)

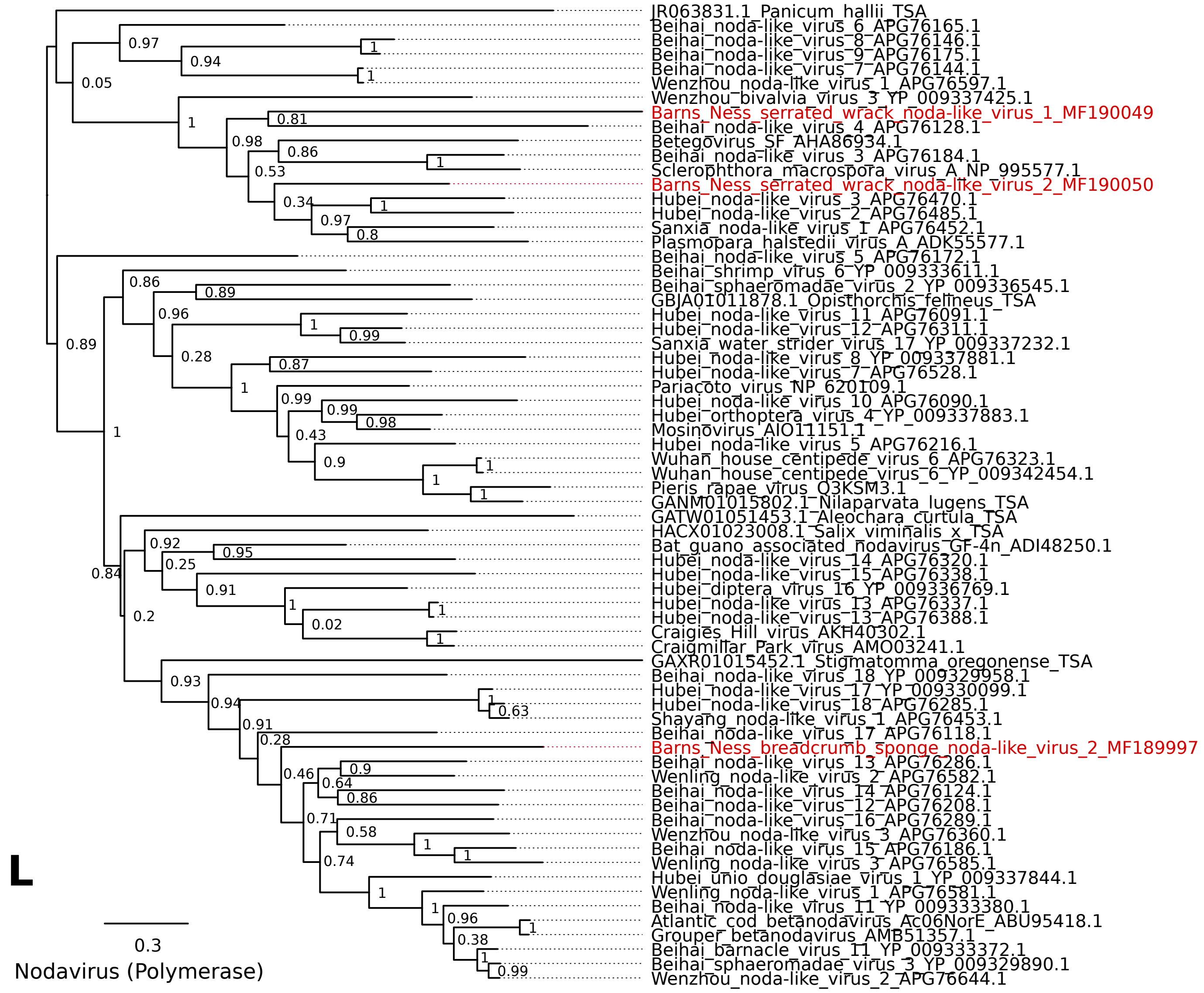
JR063831.1_Panicum_hallii_TSA
Beihai_noda-like_virus_6_APG76165.1
Beihai_noda-like_virus_8_APG76146.1
Beihai_noda-like_virus_9_APG76175.1
Beihai_noda-like_virus_7_APG76144.1
Wenzhou_noda-like_virus_1_APG76597.1
Wenzhou_bivalvia_virus_3_YP_009337425.1
Barns_Ness_serrated_wrack_noda-like_virus_1_MF190049
Beihai_noda-like_virus_4_APG76128.1
Betegovirus_SF_AHA86934.1
Beihai_noda-like_virus_3_APG76184.1
Sclerophthora_macrospora_virus_A_NP_995577.1
Barns_Ness_serrated_wrack_noda-like_virus_2_MF190050
Hubei_noda-like_virus_3_APG76470.1
Hubei_noda-like_virus_2_APG76485.1
Sanxia_noda-like_virus_1_APG76452.1
Plasmopara_halstedii_virus_A_ADK55577.1
Beihai_noda-like_virus_5_APG76172.1
Beihai_shrimp_virus_6_YP_009333611.1
Beihai_sphaeromadae_virus_2_YP_009336545.1
GBJA01011878.1_Opisthorchis_felineus_TSA
Hubei_noda-like_virus_11_APG76091.1
Hubei_noda-like_virus_12_APG76311.1
Sanxia_water_strider_virus_17_YP_009337232.1
Hubei_noda-like_virus_8_YP_009337881.1
Hubei_noda-like_virus_7_APG76528.1
Pariacoto_virus_NP_620109.1
Hubei_noda-like_virus_10_APG76090.1
Hubei_orthoptera_virus_4_YP_009337883.1
Mosinovirus_AIO11151.1
Hubei_noda-like_virus_5_APG76216.1
Wuhan_house_centipede_virus_6_APG76323.1
Wuhan_house_centipede_virus_6_YP_009342454.1
Pieris_rapae_virus_Q3KSM3.1
GANM01015802.1_Nilaparvata_lugens_TSA
GATW01051453.1_Aleochara_curtula_TSA
HACX01023008.1_Salix_viminalis_x_TSA
Bat_guano_associated_nodavirus_GF-4n_ADI48250.1
Hubei_noda-like_virus_14_APG76320.1
Hubei_noda-like_virus_15_APG76338.1
Hubei_diptera_virus_16_YP_009336769.1
Hubei_noda-like_virus_13_APG76337.1
Hubei_noda-like_virus_13_APG76388.1
Craigies_Hill_virus_AKH40302.1
Craigmillar_Park_virus_AMO03241.1
GAXR01015452.1_Stigmatomma_oregonense_TSA
Beihai_noda-like_virus_18_YP_009329958.1
Hubei_noda-like_virus_17_YP_009330099.1
Hubei_noda-like_virus_18_APG76285.1
Shayang_noda-like_virus_1_APG76453.1
Beihai_noda-like_virus_17_APG76118.1
Barns_Ness_breadcrumb_sponge_noda-like_virus_2_MF189997
Beihai_noda-like_virus_13_APG76286.1
Wenling_noda-like_virus_2_APG76582.1
Beihai_noda-like_virus_14_APG76124.1
Beihai_noda-like_virus_12_APG76208.1
Beihai_noda-like_virus_16_APG76289.1
Wenzhou_noda-like_virus_3_APG76360.1
Beihai_noda-like_virus_15_APG76186.1
Wenling_noda-like_virus_3_APG76585.1
Hubei_unio_douglasiae_virus_1_YP_009337844.1
Wenling_noda-like_virus_1_APG76581.1
Beihai_noda-like_virus_11_YP_009333380.1
Atlantic_cod_betanodavirus_Ac06NorE_ABU95418.1
Grouper_betanodavirus_AMB51357.1
Beihai_barnacle_virus_11_YP_009333372.1
Beihai_sphaeromadae_virus_3_YP_009329890.1
Wenzhou_noda-like_virus_2_APG76644.1
L
0.3
Nodavirus (Polymerase)

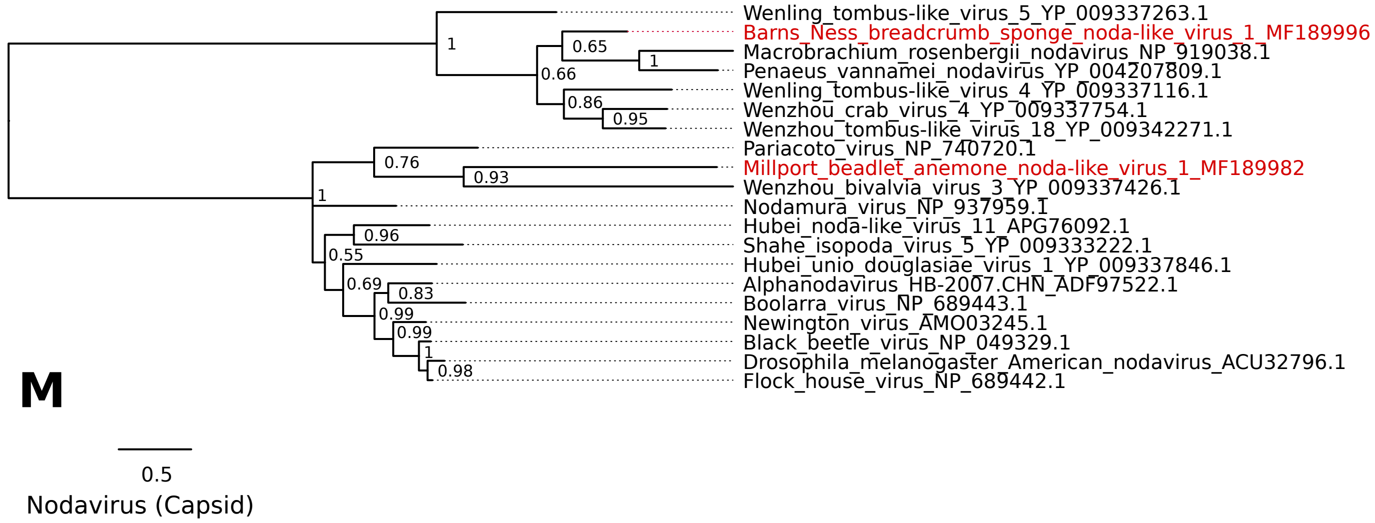
Wenling_tombus-like_virus_5_YP_009337263.1
Barns_Ness_breadcrumb_sponge_noda-like_virus_1_MF189996
Macrobrachium_rosenbergii_nodavirus_NP_919038.1
Penaeus_vannamei_nodavirus_YP_004207809.1
Wenling_tombus-like_virus_4_YP_009337116.1
Wenzhou_crab_virus_4_YP_009337754.1
Wenzhou_tombus-like_virus_18_YP_009342271.1
Pariacoto_virus_NP_740720.1
Millport_beadlet_anemone_noda-like_virus_1_MF189982
Wenzhou_bivalvia_virus_3_YP_009337426.1
Nodamura_virus_NP_937959.1
Hubei_noda-like_virus_11_APG76092.1
Shahe_isopoda_virus_5_YP_009333222.1
Hubei_unio_douglasiae_virus_1_YP_009337846.1
Alphanodavirus_HB-2007.CHN_ADF97522.1
Boolarra_virus_NP_689443.1
Newington_virus_AMO03245.1
Black_beetle_virus_NP_049329.1
Drosophila_melanogaster_American_nodavirus_ACU32796.1
Flock_house_virus_NP_689442.1
M
0.5
Nodavirus (Capsid)

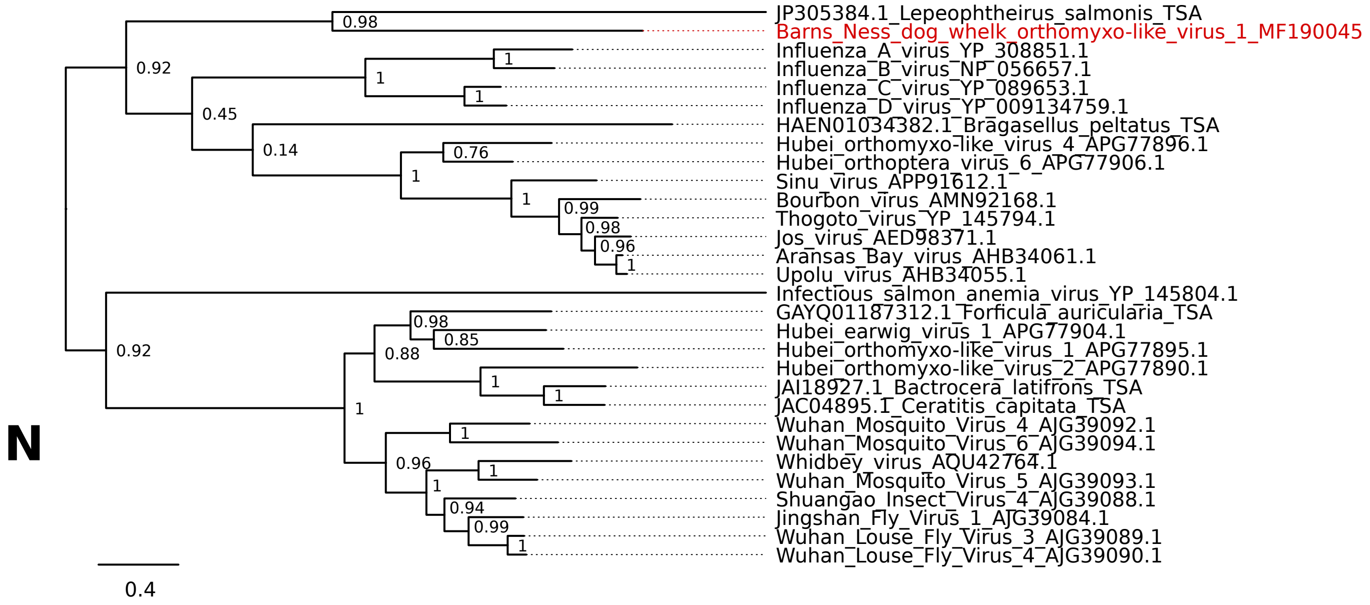

Orthomyxoviruses (PB1)

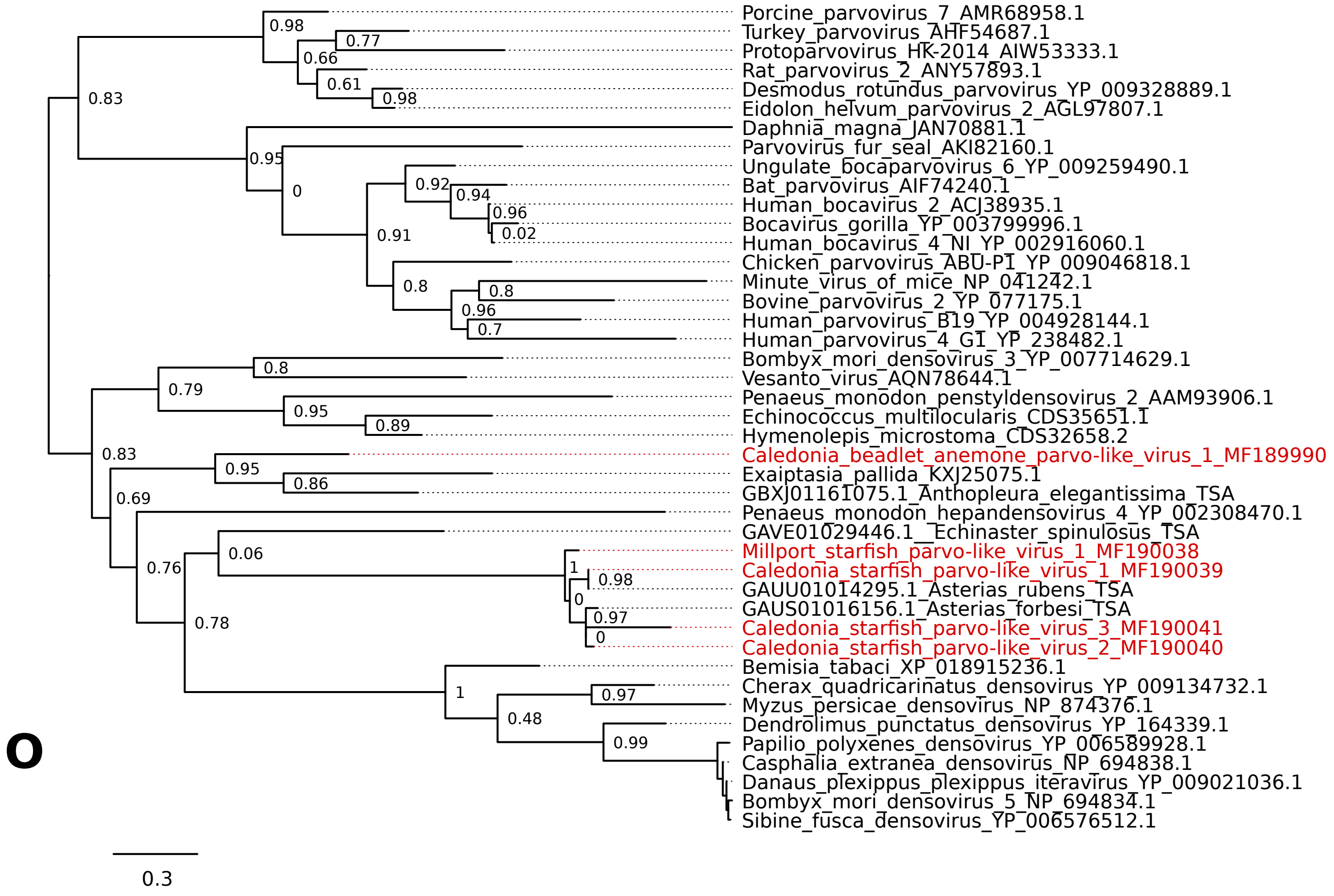

Densoviruses and Parvoviruses

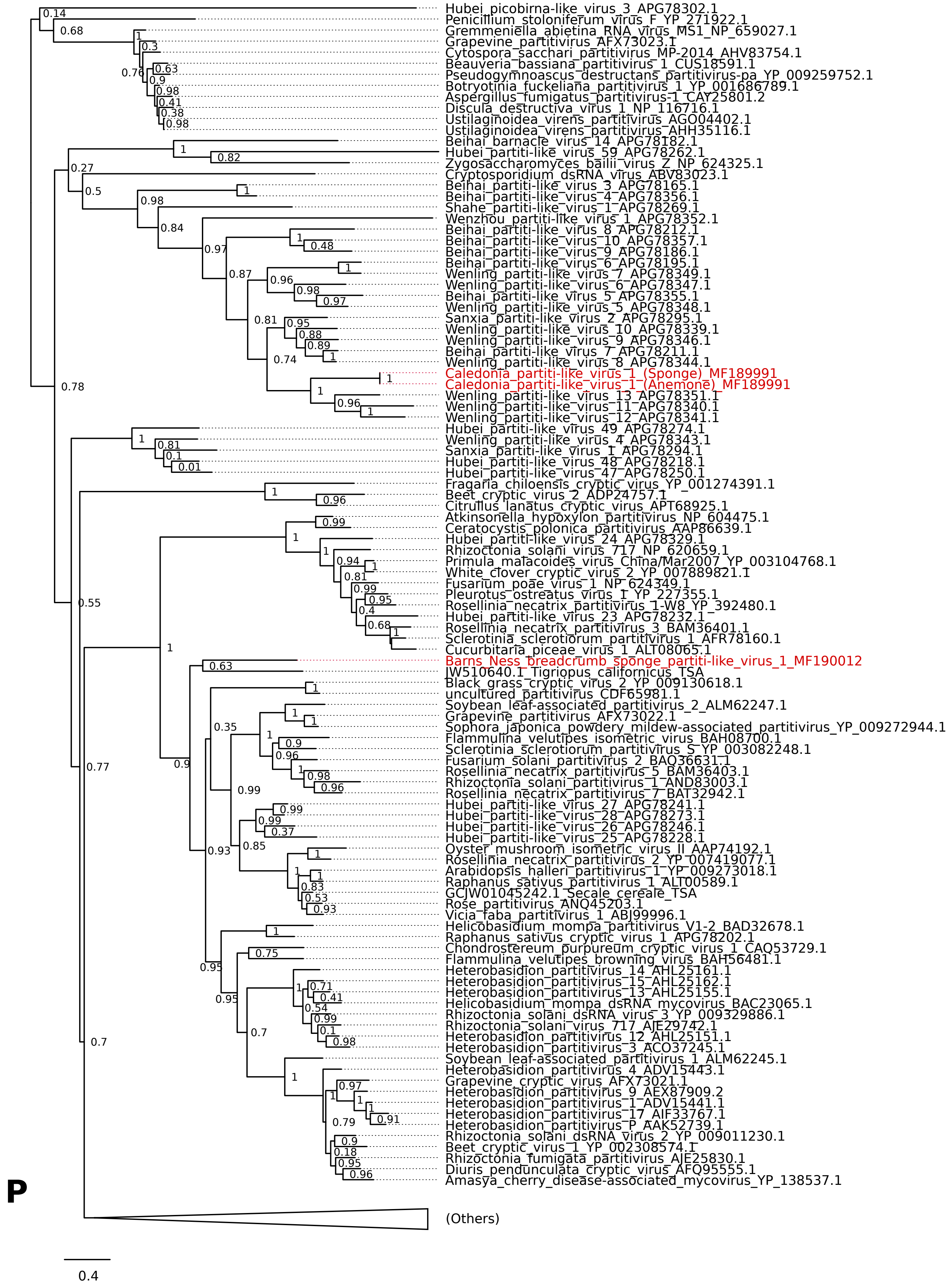

Partitiviruses (Polymerase)

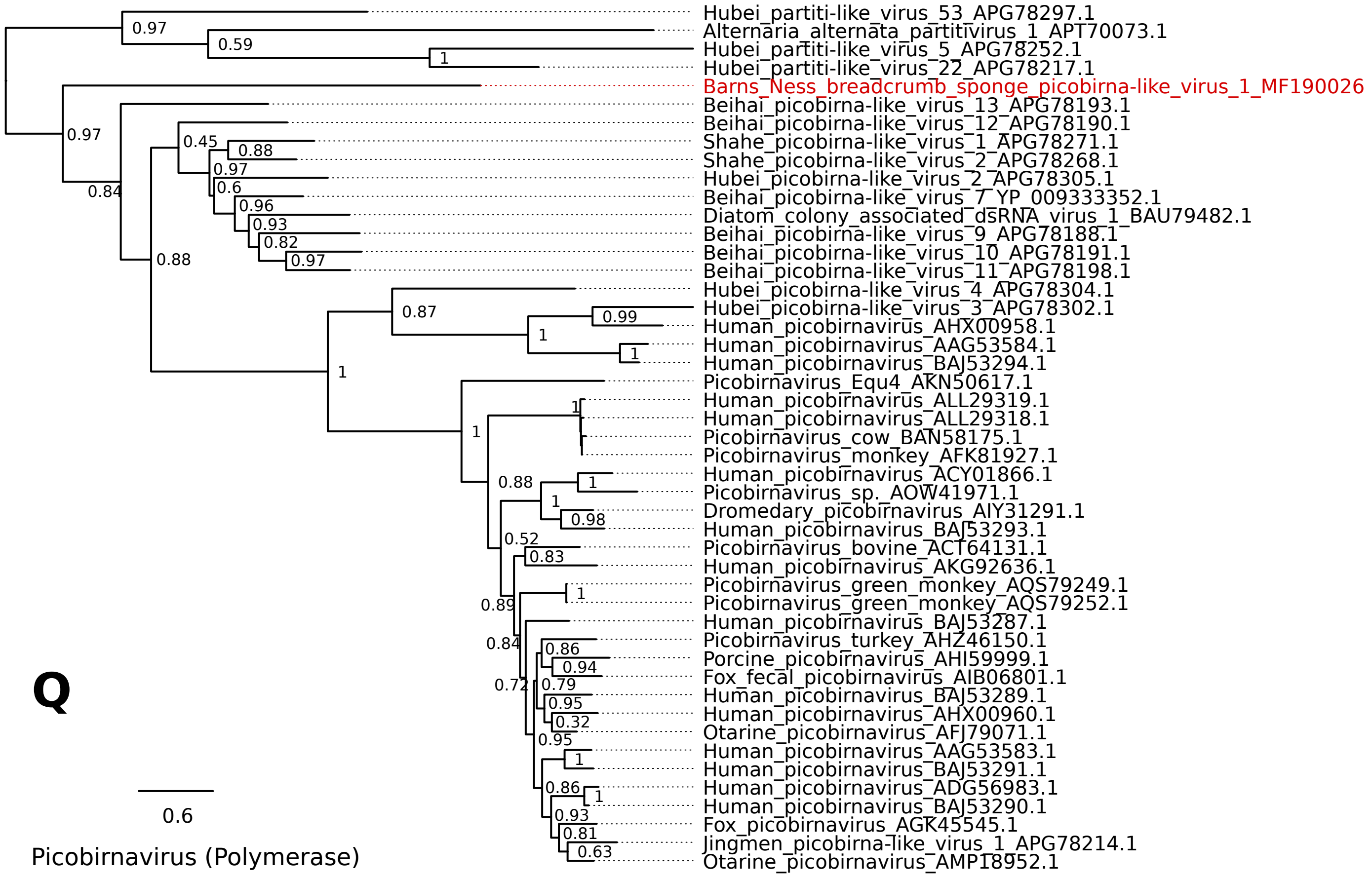

Q

Picobirnavirus (Polymerase)

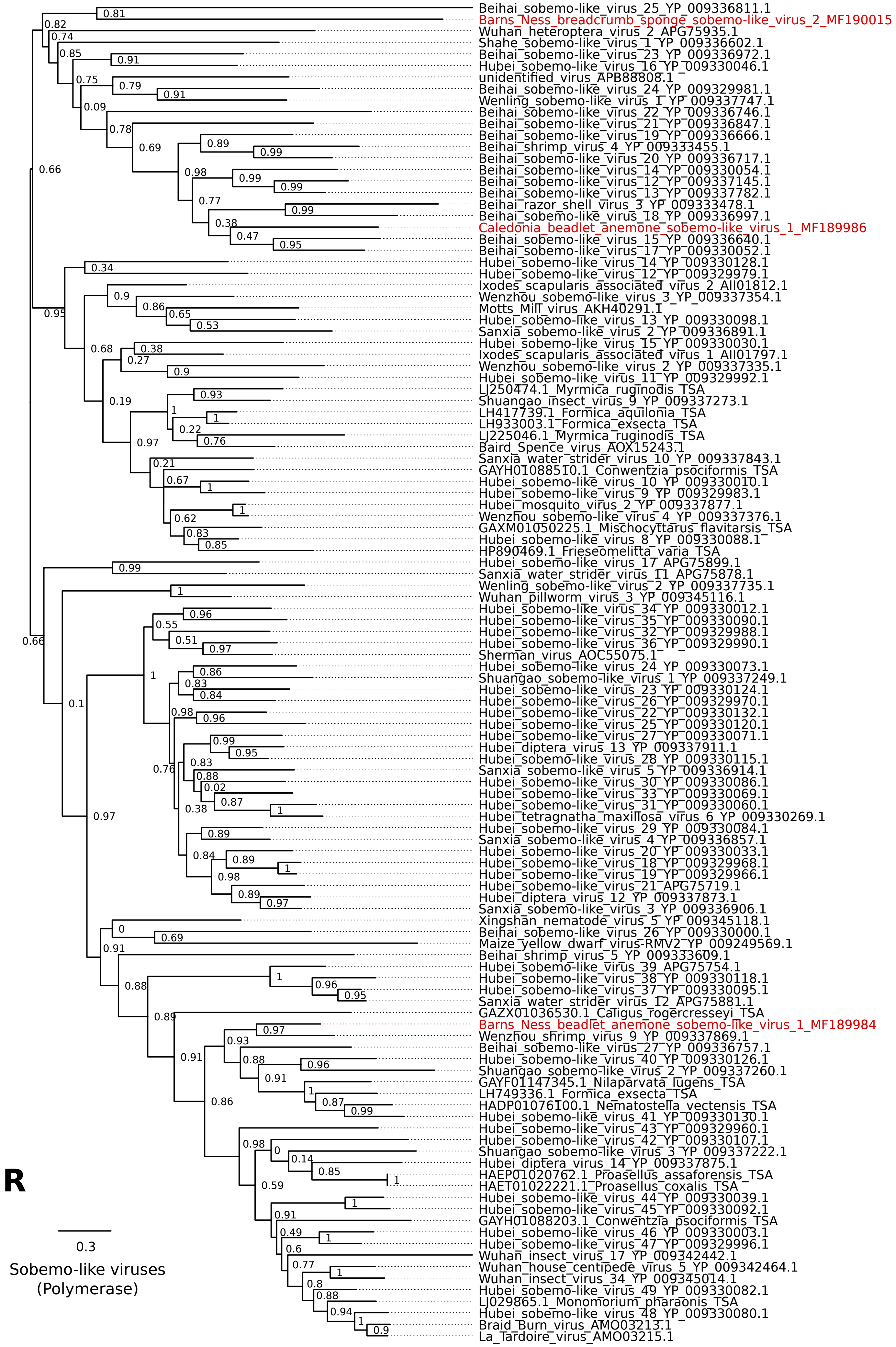

Beihai_sobemo-like_virus_25_YP_009336811.1
Barns_Ness_breadcrumb_sponge_sobemo-like_virus_2_MF190015
Wuhan_heteroptera_virus_2_APG75935.1
Shahe_sobemo-like_virus_1_YP_009336602.1
Beihai_sobemo-like_virus_23_YP_009336972.1
Hubei_sobemo-like_virus_16_YP_009330046.1
unidentified_virus_APB88808.1
Beihai_sobemo-like_virus_24_YP_009329981.1
Wenling_sobemo-like_virus_1_YP_009337747.1
Beihai_sobemo-like_virus_22_YP_009336746.1
Beihai_sobemo-like_virus_21_YP_009336847.1
Beihai_sobemo-like_virus_19_YP_009336666.1
Beihai_shrimp_virus_4_YP_009333455.1
Beihai_sobemo-like_virus_20_YP_009336717.1
Beihai_sobemo-like_virus_14_YP_009330054.1
Beihai_sobemo-like_virus_12_YP_009337145.1
Beihai_sobemo-like_virus_13_YP_009337782.1
Beihai_razor_shell_virus_3_YP_009333478.1
Beihai_sobemo-like_virus_18_YP_009336997.1
Caledonia_beadlet_anemone_sobemo-like_virus_1_MF189986
Beihai_sobemo-like_virus_15_YP_009336640.1
Beihai_sobemo-like_virus_17_YP_009330052.1
Hubei_sobemo-like_virus_14_YP_009330128.1
Hubei_sobemo-like_virus_12_YP_009329979.1
Ixodes_scapularis_associated_virus_2_AII01812.1
Wenzhou_sobemo-like_virus_3_YP_009337354.1
Motts_Mill_virus_AKH40291.1
Hubei_sobemo-like_virus_13_YP_009330098.1
Sanxia_sobemo-like_virus_2_YP_009336891.1
Hubei_sobemo-like_virus_15_YP_009330030.1
Ixodes_scapularis_associated_virus_1_AII01797.1
Wenzhou_sobemo-like_virus_2_YP_009337335.1
Hubei_sobemo-like_virus_11_YP_009329992.1
LJ250474.1_Myrmica_ruginodis_TSA
Shuangao_insect_virus_9_YP_009337273.1
LH417739.1_Formica_aquilonia_TSA
LH933003.1_Formica_exsecta_TSA
LJ225046.1_Myrmica_ruginodis_TSA
Baird_Spence_virus_AOX15243.1
Sanxia_water_strider_virus_10_YP_009337843.1
GAYH01088510.1_Conwentzia_psociformis_TSA
Hubei_sobemo-like_virus_10_YP_009330010.1
Hubei_sobemo-like_virus_9_YP_009329983.1
Hubei_mosquito_virus_2_YP_009337877.1
Wenzhou_sobemo-like_virus_4_YP_009337376.1
GAXM01050225.1_Mischocyttarus_flavitarsis_TSA
Hubei_sobemo-like_virus_8_YP_009330088.1
HP890469.1_Frieseomelitta_varia_TSA
Hubei_sobemo-like_virus_17_APG75899.1
Sanxia_water_strider_virus_11_APG75878.1
Wenling_sobemo-like_virus_2_YP_009337735.1
Wuhan_pillworm_virus_3_YP_009345116.1
Hubei_sobemo-like_virus_34_YP_009330012.1
Hubei_sobemo-like_virus_35_YP_009330090.1
Hubei_sobemo-like_virus_32_YP_009329988.1
Hubei_sobemo-like_virus_36_YP_009329990.1
Sherman_virus_AOC55075.1
Hubei_sobemo-like_virus_24_YP_009330073.1
Shuangao_sobemo-like_virus_1_YP_009337249.1
Hubei_sobemo-like_virus_23_YP_009330124.1
Hubei_sobemo-like_virus_26_YP_009329970.1
Hubei_sobemo-like_virus_22_YP_009330132.1
Hubei_sobemo-like_virus_25_YP_009330120.1
Hubei_sobemo-like_virus_27_YP_009330071.1
Hubei_diptera_virus_13_YP_009337911.1
Hubei_sobemo-like_virus_28_YP_009330115.1
Sanxia_sobemo-like_virus_5_YP_009336914.1
Hubei_sobemo-like_virus_30_YP_009330086.1
Hubei_sobemo-like_virus_33_YP_009330069.1
Hubei_sobemo-like_virus_31_YP_009330060.1
Hubei_tetragnatha_maxillosa_virus_6_YP_009330269.1
Hubei_sobemo-like_virus_29_YP_009330084.1
Sanxia_sobemo-like_virus_4_YP_009336857.1
Hubei_sobemo-like_virus_20_YP_009330033.1
Hubei_sobemo-like_virus_18_YP_009329968.1
Hubei_sobemo-like_virus_19_YP_009329966.1
Hubei_sobemo-like_virus_21_APG75719.1
Hubei_diptera_virus_12_YP_009337873.1
Sanxia_sobemo-like_virus_3_YP_009336906.1
Xingshan_nematode_virus_5_YP_009345118.1
Beihai_sobemo-like_virus_26_YP_009330000.1
Maize_yellow_dwarf_virus-RMV2_YP_009249569.1
Beihai_shrimp_virus_5_YP_009333609.1
Hubei_sobemo-like_virus_39_APG75754.1
Hubei_sobemo-like_virus_38_YP_009330118.1
Hubei_sobemo-like_virus_37_YP_009330095.1
Sanxia_water_strider_virus_12_APG75881.1
GAZX01036530.1_Caligus_rogercresseyi_TSA
Barns_Ness_beadlet_anemone_sobemo-like_virus_1_MF189984
Wenzhou_shrimp_virus_9_YP_009337869.1
Beihai_sobemo-like_virus_27_YP_009336757.1
Hubei_sobemo-like_virus_40_YP_009330126.1
Shuangao_sobemo-like_virus_2_YP_009337260.1
GAYF01147345.1_Nilaparvata_lugens_TSA
LH749336.1_Formica_exsecta_TSA
HADP01076100.1_Nematostella_vectensis_TSA
Hubei_sobemo-like_virus_41_YP_009330130.1
Hubei_sobemo-like_virus_43_YP_009329960.1
Hubei_sobemo-like_virus_42_YP_009330107.1
Shuangao_sobemo-like_virus_3_YP_009337222.1
Hubei_diptera_virus_14_YP_009337875.1
HAEP01020762.1_Proasellus_assaforensis_TSA
HAET01022221.1_Proasellus_coxalis_TSA
Hubei_sobemo-like_virus_44_YP_009330039.1
Hubei_sobemo-like_virus_45_YP_009330092.1
GAYH01088203.1_Conwentzia_psociformis_TSA
Hubei_sobemo-like_virus_46_YP_009330003.1
Hubei_sobemo-like_virus_47_YP_009329996.1
Wuhan_insect_virus_17_YP_009342442.1
Wuhan_house_centipede_virus_5_YP_009342464.1
Wuhan_insect_virus_34_YP_009345014.1
Hubei_sobemo-like_virus_49_YP_009330082.1
LJ029865.1_Monomorium_pharaonis_TSA
Hubei_sobemo-like_virus_48_YP_009330080.1
Braid_Burn_virus_AMO03213.1
La_Tardoire_virus_AMO03215.1
R
0.3
Sobemo-like viruses
(Polymerase)

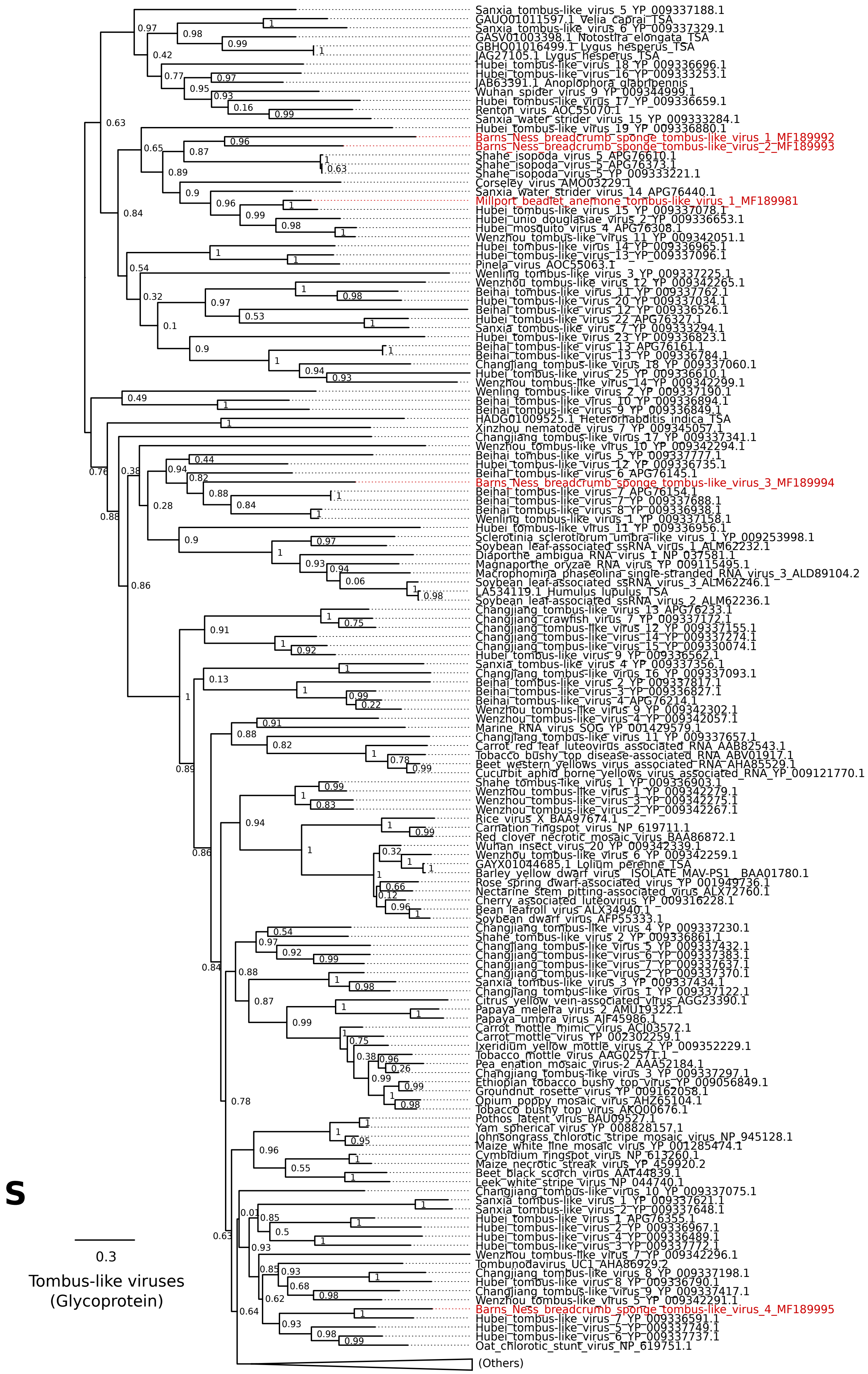
Sanxia_tombus-like_virus_5_YP_009337188.1
GAUQ01011597.1_Velia_caprai_TSA
Sanxia_tombus-like_virus_6_YP_009337329.1
GASV01003398.1_Notostira_elongata_TSA
GBHO01016499.1_Lygus_hesperus_TSA
JAG27105.1_Lygus_hesperus_TSA
Hubei_tombus-like_virus_18_YP_009336696.1
Hubei_tombus-like_virus_16_YP_009333253.1
JAB63391.1_Anoplophora_glabripennis
Wuhan_spider_virus_9_YP_009344999.1
Hubei_tombus-like_virus_17_YP_009336659.1
Renton_virus_AOC55070.1
Sanxia_water_strider_virus_15_YP_009333284.1
Hubei_tombus-like_virus_19_YP_009336880.1
Barns_Ness_breadcrumb_sponge_tombus-like_virus_1_MF189992
Barns_Ness_breadcrumb_sponge_tombus-like_virus_2_MF189993
Shahe_isopoda_virus_5_APG76610.1
Shahe_isopoda_virus_5_APG76373.1
Shahe_isopoda_virus_5_YP_009333221.1
Corseley_virus_AMO03229.1
Sanxia_water_strider_virus_14_APG76440.1
Millport_beadlet_anemone_tombus-like_virus_1_MF189981
Hubei_tombus-like_virus_15_YP_009337078.1
Hubei_unio_douglasiae_virus_2_YP_009336653.1
Hubei_mosquito_virus_4_APG76308.1
Wenzhou_tombus-like_virus_11_YP_009342051.1
Hubei_tombus-like_virus_14_YP_009336965.1
Hubei_tombus-like_virus_13_YP_009337096.1
Pinela_virus_AOC55063.1
Wenling_tombus-like_virus_3_YP_009337225.1
Wenzhou_tombus-like_virus_12_YP_009342265.1
Beihai_tombus-like_virus_11_YP_009337762.1
Hubei_tombus-like_virus_20_YP_009337034.1
Beihai_tombus-like_virus_12_YP_009336526.1
Hubei_tombus-like_virus_22_APG76327.1
Sanxia_tombus-like_virus_7_YP_009333294.1
Hubei_tombus-like_virus_23_YP_009336823.1
Beihai_tombus-like_virus_13_APG76161.1
Beihai_tombus-like_virus_13_YP_009336784.1
Changjiang_tombus-like_virus_18_YP_009337060.1
Hubei_tombus-like_virus_25_YP_009336610.1
Wenzhou_tombus-like_virus_14_YP_009342299.1
Wenling_tombus-like_virus_2_YP_009337190.1
Beihai_tombus-like_virus_10_YP_009336894.1
Beihai_tombus-like_virus_9_YP_009336849.1
HADG01009525.1_Heterorhabditis_indica_TSA
Xinzhou_nematode_virus_7_YP_009345057.1
Changjiang_tombus-like_virus_17_YP_009337341.1
Wenzhou_tombus-like_virus_10_YP_009342294.1
Beihai_tombus-like_virus_5_YP_009337777.1
Hubei_tombus-like_virus_12_YP_009336735.1
Beihai_tombus-like_virus_6_APG76145.1
Barns_Ness_breadcrumb_sponge_tombus-like_virus_3_MF189994
Beihai_tombus-like_virus_7_APG76154.1
Beihai_tombus-like_virus_7_YP_009337688.1
Beihai_tombus-like_virus_8_YP_009336938.1
Wenling_tombus-like_virus_1_YP_009337158.1
Hubei_tombus-like_virus_11_YP_009336956.1
Sclerotinia_sclerotiorum_umbra-like_virus_1_YP_009253998.1
Soybean_leaf-associated_ssRNA_virus_1_ALM62232.1
Diaporthe_ambigua_RNA_virus_1_NP_037581.1
Magnaporthe_oryzae_RNA_virus_YP_009115495.1
Macrophomina_phaseolina_single-stranded_RNA_virus_3_ALD89104.2
Soybean_leaf-associated_ssRNA_virus_3_ALM62246.1
LA534119.1_Humulus_lupulus_TSA
Soybean_leaf-associated_ssRNA_virus_2_ALM62236.1
Changjiang_tombus-like_virus_13_APG76233.1
Changjiang_crawfish_virus_7_YP_009337172.1
Changjiang_tombus-like_virus_12_YP_009337155.1
Changjiang_tombus-like_virus_14_YP_009337274.1
Changjiang_tombus-like_virus_15_YP_009330074.1
Hubei_tombus-like_virus_9_YP_009336562.1
Sanxia_tombus-like_virus_4_YP_009337356.1
Changjiang_tombus-like_virus_16_YP_009337093.1
Beihai_tombus-like_virus_2_YP_009337817.1
Beihai_tombus-like_virus_3_YP_009336827.1
Beihai_tombus-like_virus_4_APG76214.1
Wenzhou_tombus-like_virus_9_YP_009342302.1
Wenzhou_tombus-like_virus_4_YP_009342057.1
Marine_RNA_virus_SOG_YP_001429579.1
Changjiang_tombus-like_virus_11_YP_009337657.1
Carrot_red_leaf_luteovirus_associated_RNA_AAB82543.1
Tobacco_bushy_top_disease-associated_RNA_ABV01917.1
Beet_western_yellows_virus_associated_RNA_AHA85529.1
Cucurbit_aphid_borne_yellows_virus_associated_RNA_YP_009121770.1
Shahe_tombus-like_virus_1_YP_009336903.1
Wenzhou_tombus-like_virus_1_YP_009342279.1
Wenzhou_tombus-like_virus_3_YP_009342275.1
Wenzhou_tombus-like_virus_2_YP_009342267.1
Rice_virus_X_BAA97674.1
Carnation_ringspot_virus_NP_619711.1
Red_clover_necrotic_mosaic_virus_BAA86872.1
Wuhan_insect_virus_20_YP_009342339.1
Wenzhou_tombus-like_virus_6_YP_009342259.1
GAYX01044685.1_Lolium_perenne_TSA
Barley_yellow_dwarf_virus__ISOLATE_MAV-PS1__BAA01780.1
Rose_spring_dwarf-associated_virus_YP_001949736.1
Nectarine_stem_pitting-associated_virus_ALX72760.1
Cherry_associated_luteovirus_YP_009316228.1
Bean_leafroll_virus_ALX34940.1
Soybean_dwarf_virus_AFP55333.1
Changjiang_tombus-like_virus_4_YP_009337230.1
Shahe_tombus-like_virus_2_YP_009336861.1
Changjiang_tombus-like_virus_5_YP_009337432.1
Changjiang_tombus-like_virus_6_YP_009337383.1
Changjiang_tombus-like_virus_7_YP_009337637.1
Changjiang_tombus-like_virus_2_YP_009337370.1
Sanxia_tombus-like_virus_3_YP_009337434.1
Changjiang_tombus-like_virus_1_YP_009337122.1
Citrus_yellow_vein-associated_virus_AGG23390.1
Papaya_meleira_virus_2_AMU19322.1
Papaya_umbra_virus_AJF45986.1
Carrot_mottle_mimic_virus_ACJ03572.1
Carrot_mottle_virus_YP_002302259.1
Ixeridium_yellow_mottle_virus_2_YP_009352229.1
Tobacco_mottle_virus_AAG02571.1
Pea_enation_mosaic_virus-2_AAA52184.1
Changjiang_tombus-like_virus_3_YP_009337297.1
Ethiopian_tobacco_bushy_top_virus_YP_009056849.1
Groundnut_rosette_virus_YP_009162058.1
Opium_poppy_mosaic_virus_AHZ65104.1
Tobacco_bushy_top_virus_AKQ00676.1
Pothos_latent_virus_BAU09527.1
Yam_spherical_virus_YP_008828157.1
Johnsongrass_chlorotic_stripe_mosaic_virus_NP_945128.1
Maize_white_line_mosaic_virus_YP_001285474.1
Cymbidium_ringspot_virus_NP_613260.1
Maize_necrotic_streak_virus_YP_459920.2
Beet_black_scorch_virus_AAT44839.1
Leek_white_stripe_virus_NP_044740.1
Changjiang_tombus-like_virus_10_YP_009337075.1
Sanxia_tombus-like_virus_1_YP_009337621.1
Sanxia_tombus-like_virus_2_YP_009337648.1
Hubei_tombus-like_virus_1_APG76355.1
Hubei_tombus-like_virus_2_YP_009336967.1
Hubei_tombus-like_virus_4_YP_009336489.1
Hubei_tombus-like_virus_3_YP_009337772.1
Wenzhou_tombus-like_virus_7_YP_009342296.1
Tombunodavirus_UC1_AHA86929.2
Changjiang_tombus-like_virus_8_YP_009337198.1
Hubei_tombus-like_virus_8_YP_009336790.1
Changjiang_tombus-like_virus_9_YP_009337417.1
Wenzhou_tombus-like_virus_5_YP_009342291.1
Barns_Ness_breadcrumb_sponge_tombus-like_virus_4_MF189995
Hubei_tombus-like_virus_7_YP_009336591.1
Hubei_tombus-like_virus_5_YP_009337749.1
Hubei_tombus-like_virus_6_YP_009337737.1
Oat_chlorotic_stunt_virus_NP_619751.1
(Others)
S
0.3
Tombus-like viruses
(Glycoprotein)

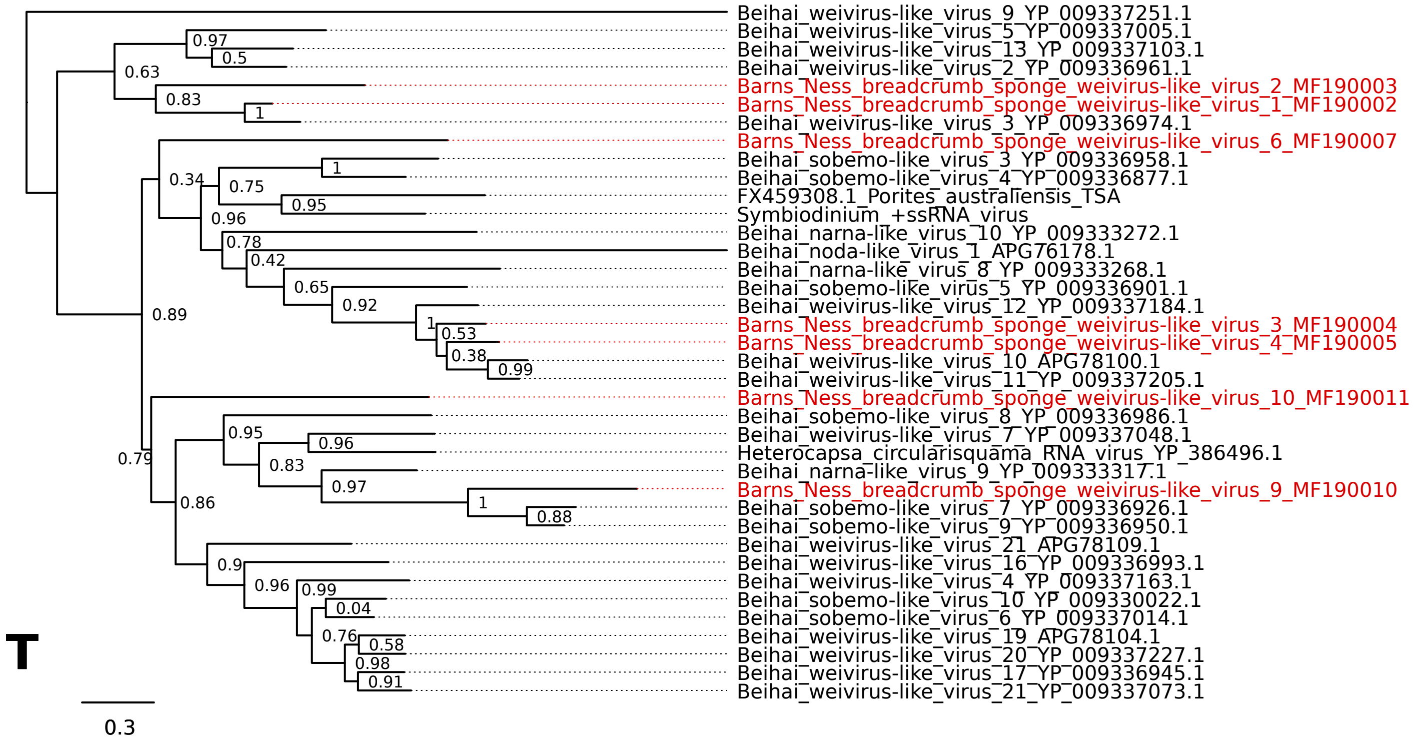

Weivirus (Capsid)

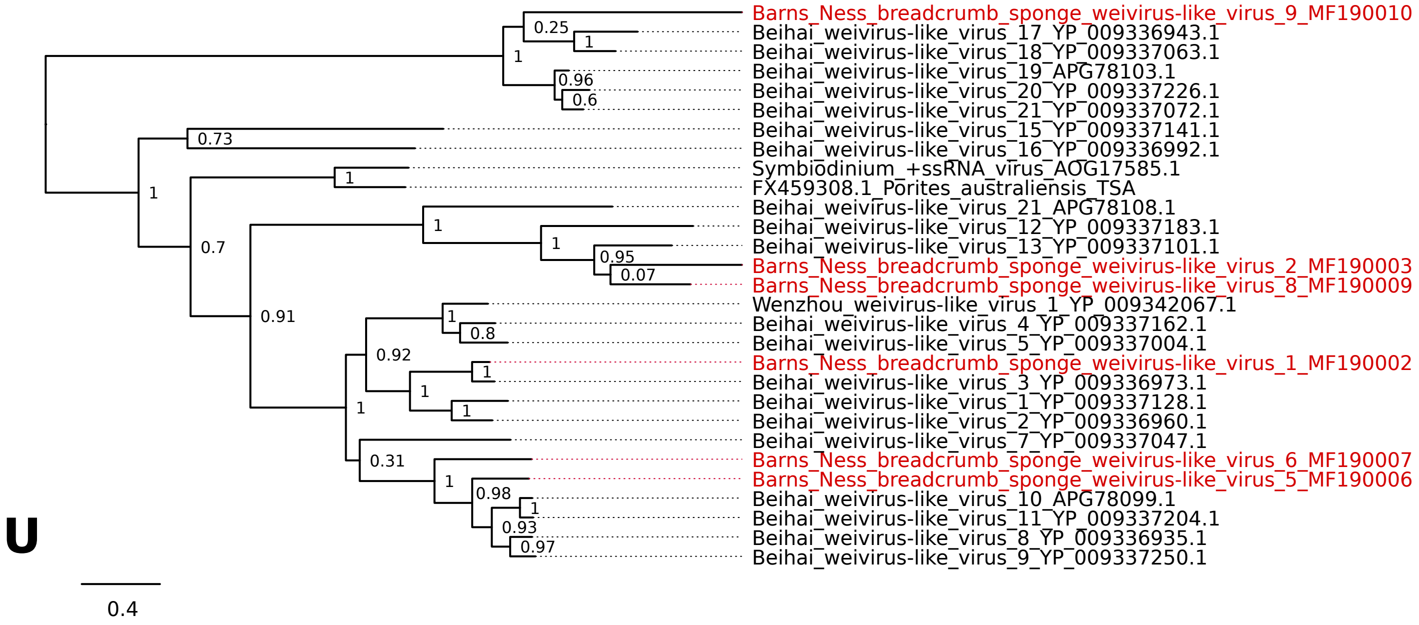

Weivirus (Polymerase)
