# Supplementary material for: Metagenomic sequencing suggests a diversity of RNA interference-like responses to viruses across multicellular eukaryotes
Source: PLoS Genet. 2018 Jul 30;14(7):e1007533. doi: 10.1371/journal.pgen.1007533 (PMC6085071; doi:10.1371/journal.pgen.1007533)
Supplement: S5 Fig — Bar-plot size distributions of all small RNAs sequences. Columns correspond to species, rows to libraries. Panel A: All sRNAs from each library. B: sRNAs mapping to ribosomal sequences. Note that in most species read abundance decreases with size, indicative of degradation products, but that distinct peaks are visible in the oxidised libraries, consistent with specific short rRNAs possessing a 3' 2-O-methyl group. C: sRNAs mapping to known miRNA stem-loops from miRbase [124]. The proportion of putative miRNAs decreases dramatically in all oxidised libraries except the sea anemone, suggesting that miRNAs in this species possess 3' 2-O-methyl groups. The small number of mapped miRNA reads in the brown alga is probably a result of the under-representation of close relatives in miRbase [124]. D: sRNAs mapping to putative RNA virus contigs. Only the dog whelk has a large and distinctive distribution of virus-derived sRNAs, and these increase in the oxidised library, suggesting that they possess 3' 2-O-methyl groups. The small number of very short virus-derived reads in the sponge are consistent with degradation products. E: sRNAs mapping to DNA parvovirus-like contigs. These increase in the oxidised library, suggesting that they possess 3' 2-O-methyl groups. F: sRNAs mapping to selected TE-like contigs. These vary in their size range among species (21nt in the brown alga, bimodal in the sponge, peaking at 28-29nt in the other species), and increase in the oxidised library, suggesting that they possess 3'-2-O-methyl groups. Only a small proportion of TE-like contigs were used as mapping targets, and many TE-derived small RNAs remain unmapped. G: Unmapped sRNAs, comprising those that derived from divergent miRNAs, unrecognised viral contigs, TEs that were excluded from panel F, and all other sources. The data required to plot these figs are provided in S5 Table. (PDF) [file pgen.1007533.s005.pdf]

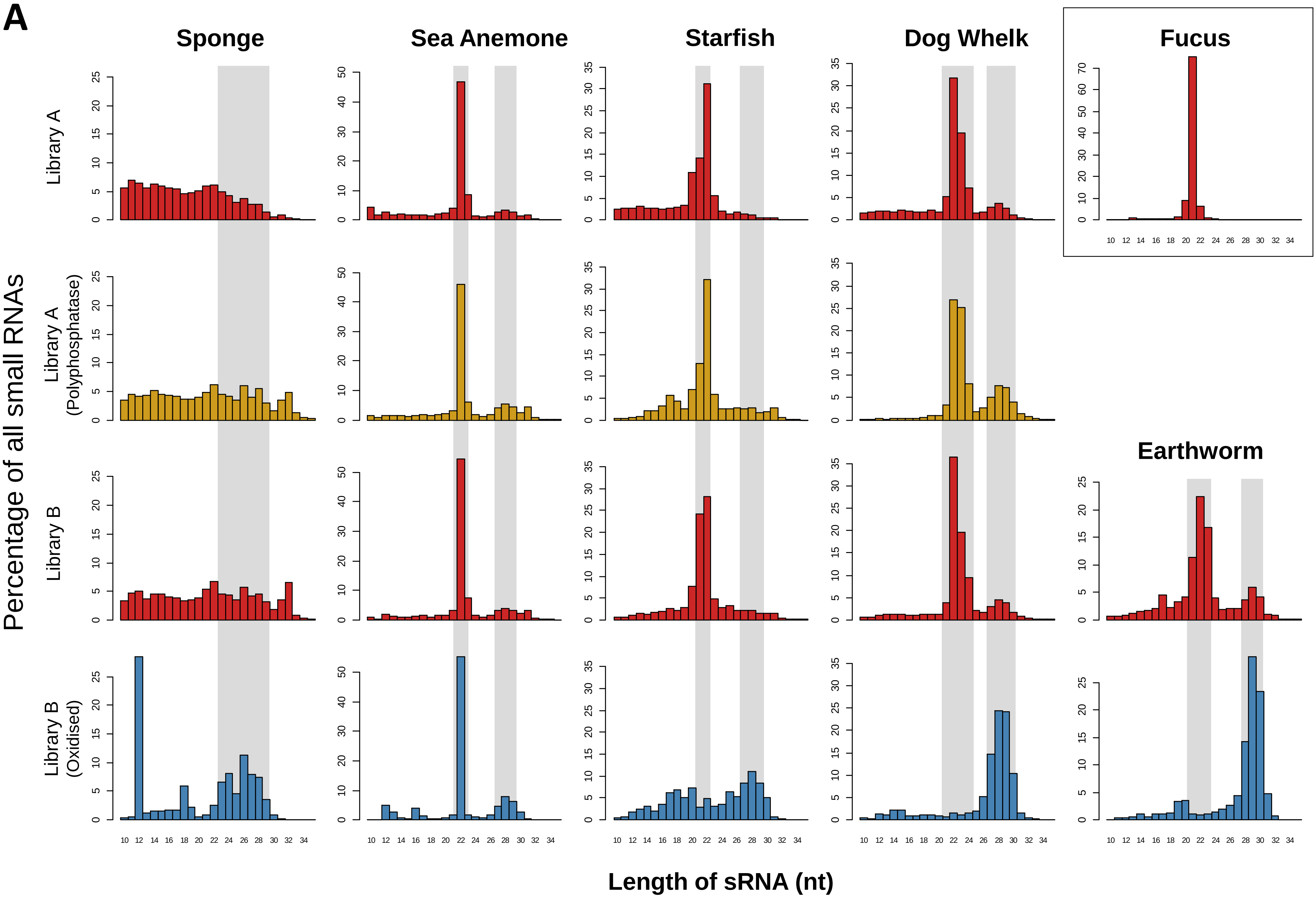

A
Sponge
Sea Anemone
Starfish
Dog Whelk
Fucus
Earthworm
Library A
Library A (Polyphosphatase)
Library B
Library B (Oxidised)
Percentage of all small RNAs
Length of sRNA (nt)

B

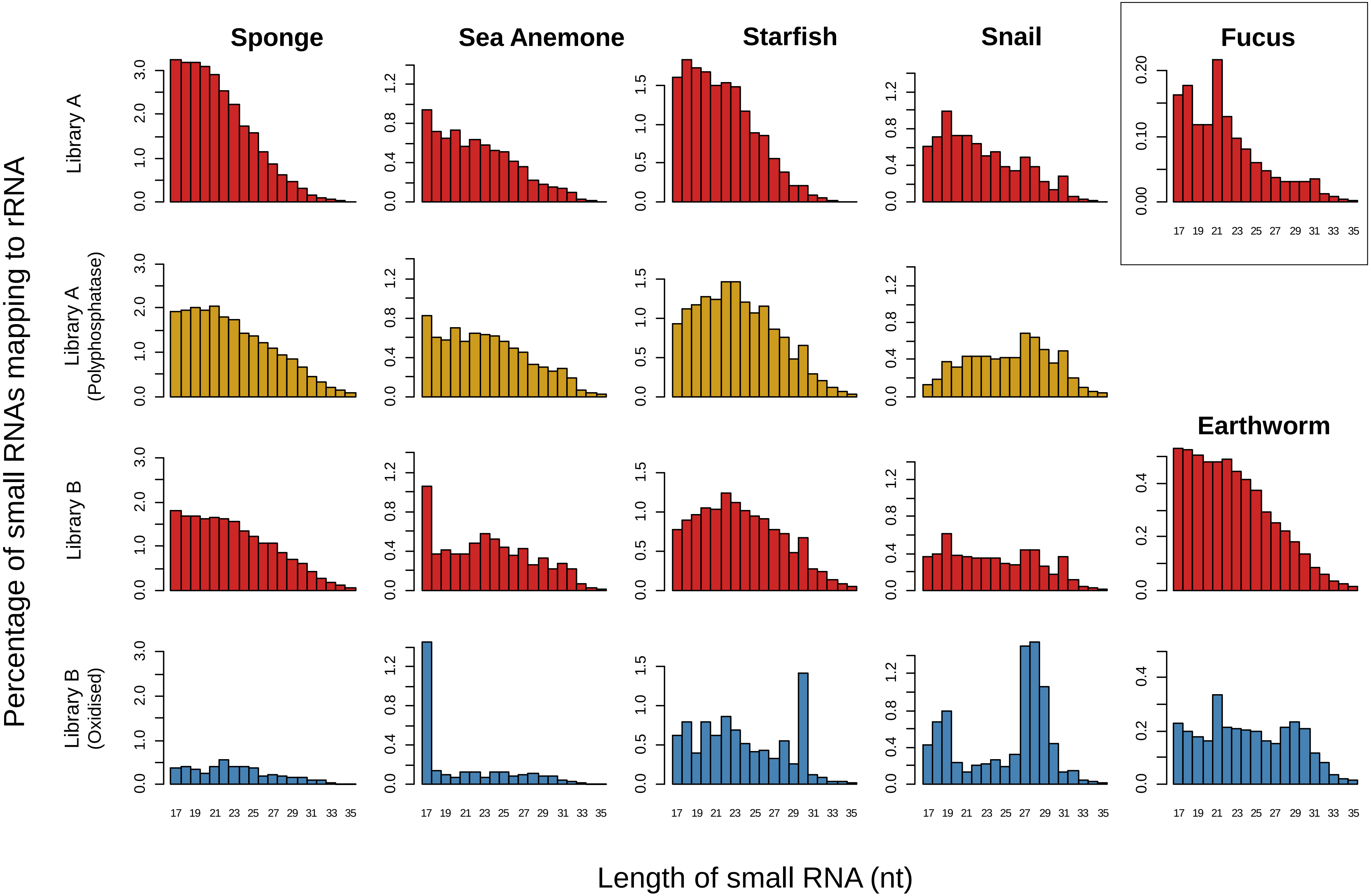
Sponge
Sea Anemone
Starfish
Snail
Fucus
Earthworm
Library A
Library A (Polyphosphatase)
Library B
Library B (Oxidised)
Percentage of small RNAs mapping to rRNA
Length of small RNA (nt)

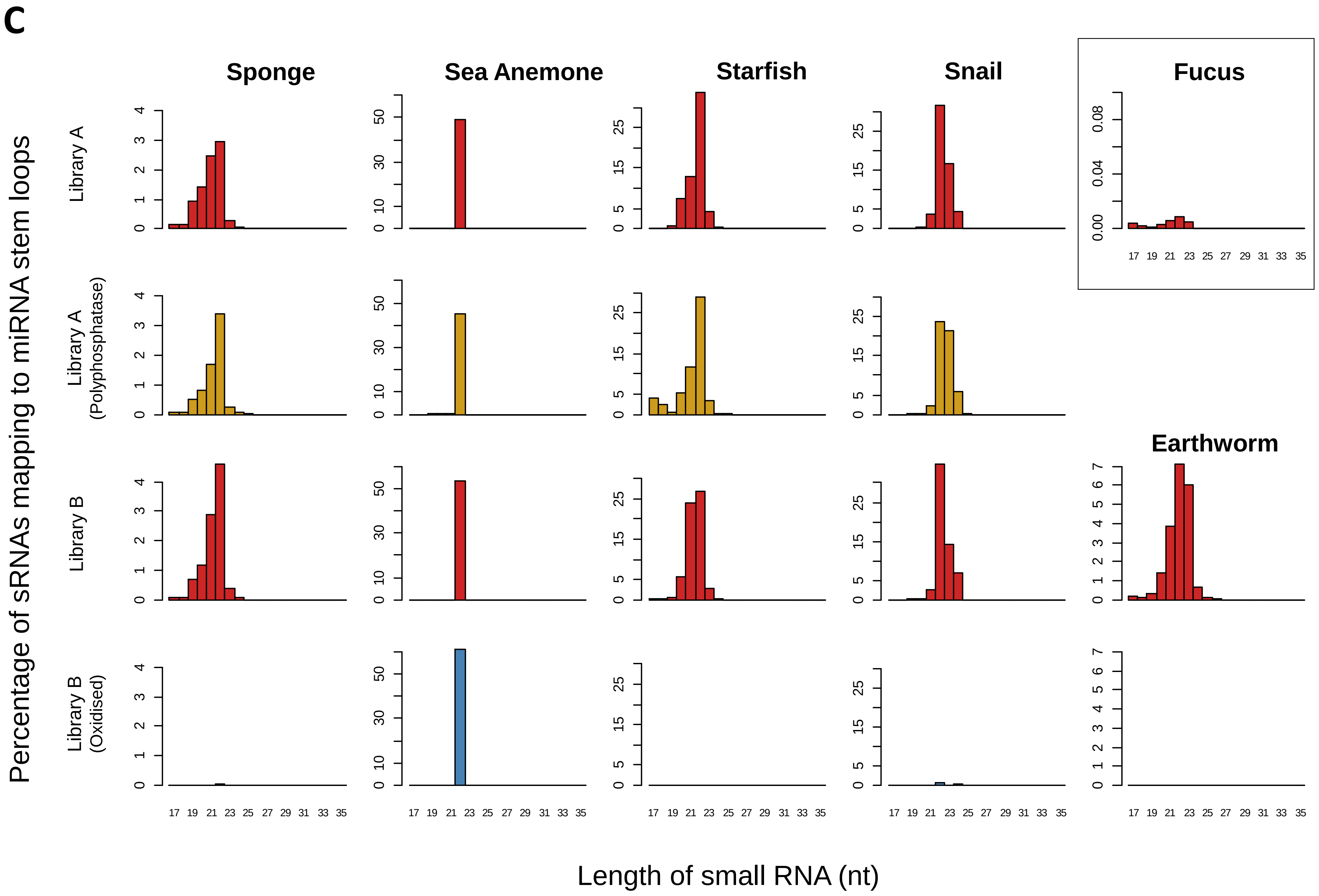
C
Sponge
Sea Anemone
Starfish
Snail
Fucus
Earthworm
Library A
Library A (Polyphosphatase)
Library B
Library B (Oxidised)
Percentage of sRNAs mapping to miRNA stem loops
Length of small RNA (nt)
17 19 21 23 25 27 29 31 33 35

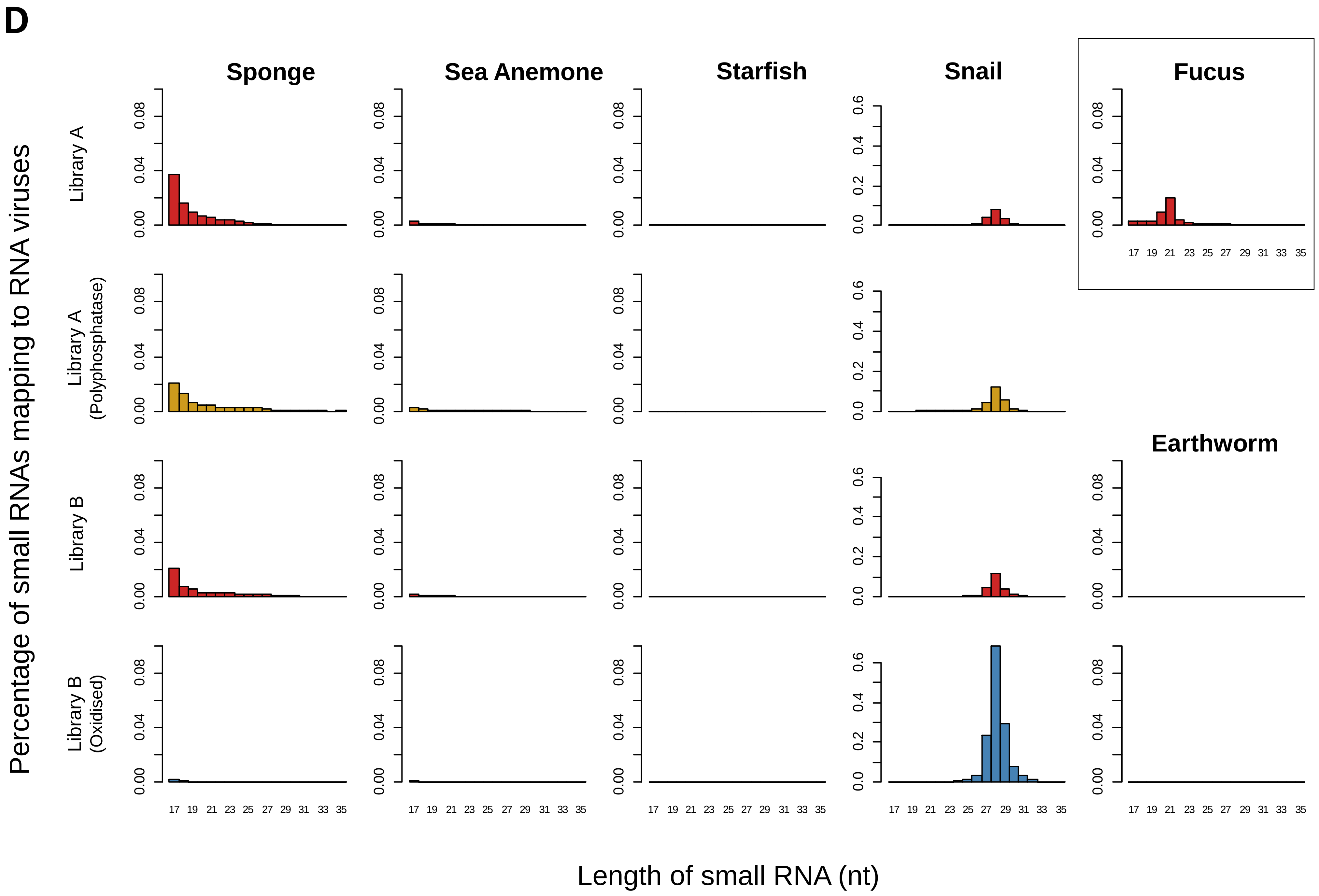
D
Sponge
Sea Anemone
Starfish
Snail
Fucus
Earthworm
Library A
Library A (Polyphosphatase)
Library B
Library B (Oxidised)
Percentage of small RNAs mapping to RNA viruses
Length of small RNA (nt)
0.00
0.04
0.08
0.0
0.2
0.4
0.6
17 19 21 23 25 27 29 31 33 35

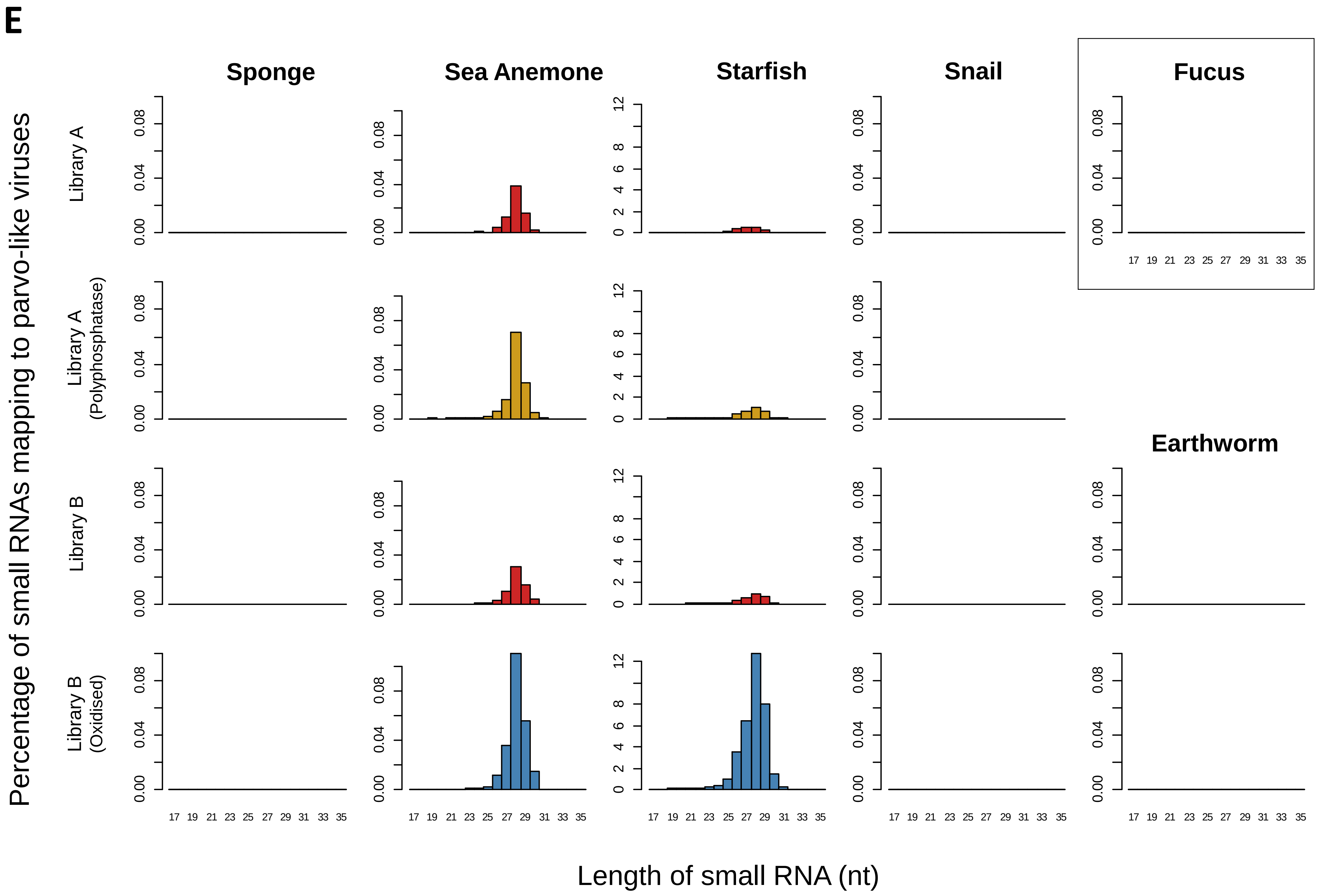
E
Sponge
Sea Anemone
Starfish
Snail
Fucus
Earthworm
Library A
Library A (Polyphosphatase)
Library B
Library B (Oxidised)
Percentage of small RNAs mapping to parvo-like viruses
Length of small RNA (nt)
0.00
0.04
0.08
0
2
4
6
8
12
17 19 21 23 25 27 29 31 33 35

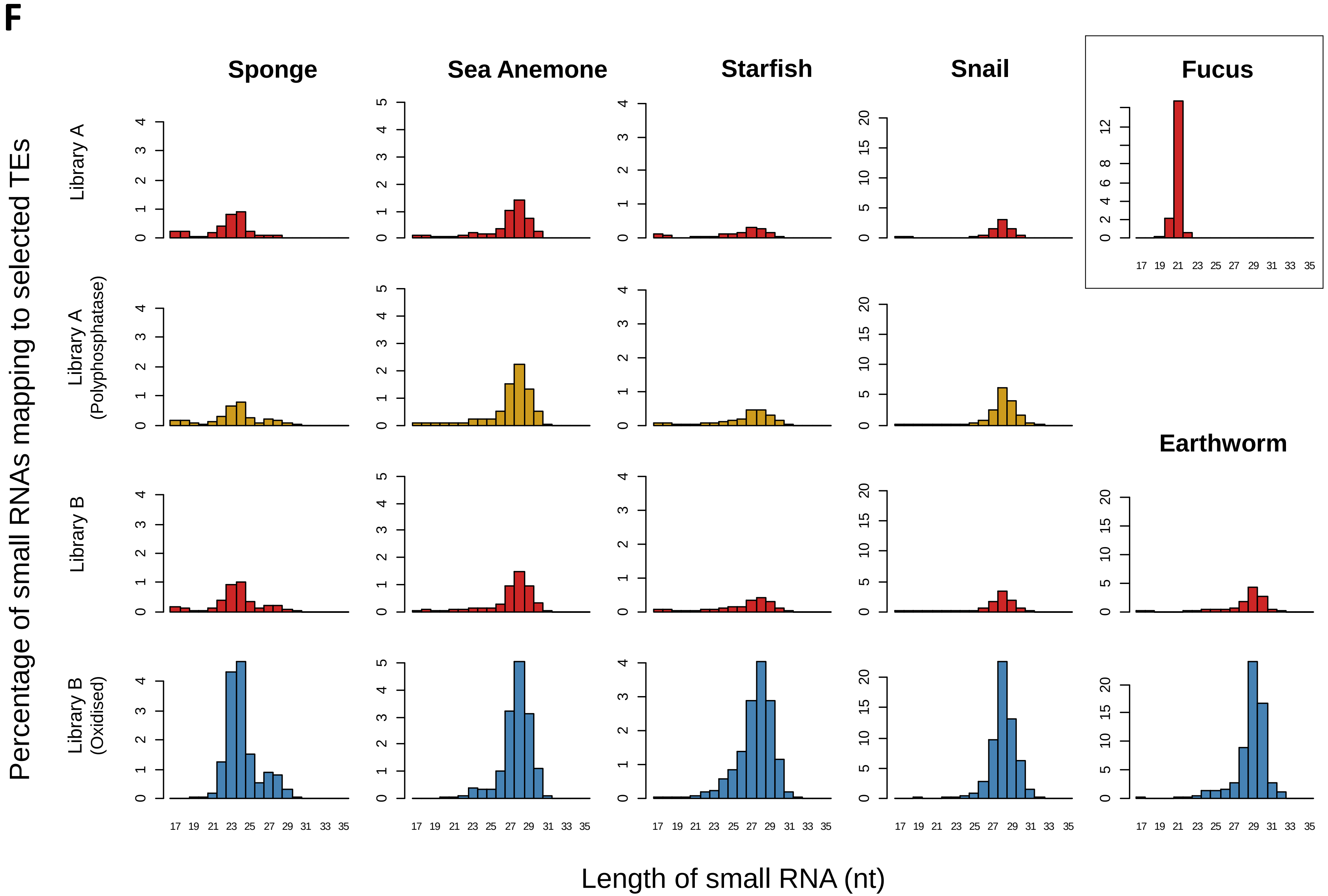
F
Sponge
Sea Anemone
Starfish
Snail
Fucus
Earthworm
Library A
Library A (Polyphosphatase)
Library B
Library B (Oxidised)
Percentage of small RNAs mapping to selected TEs
Length of small RNA (nt)

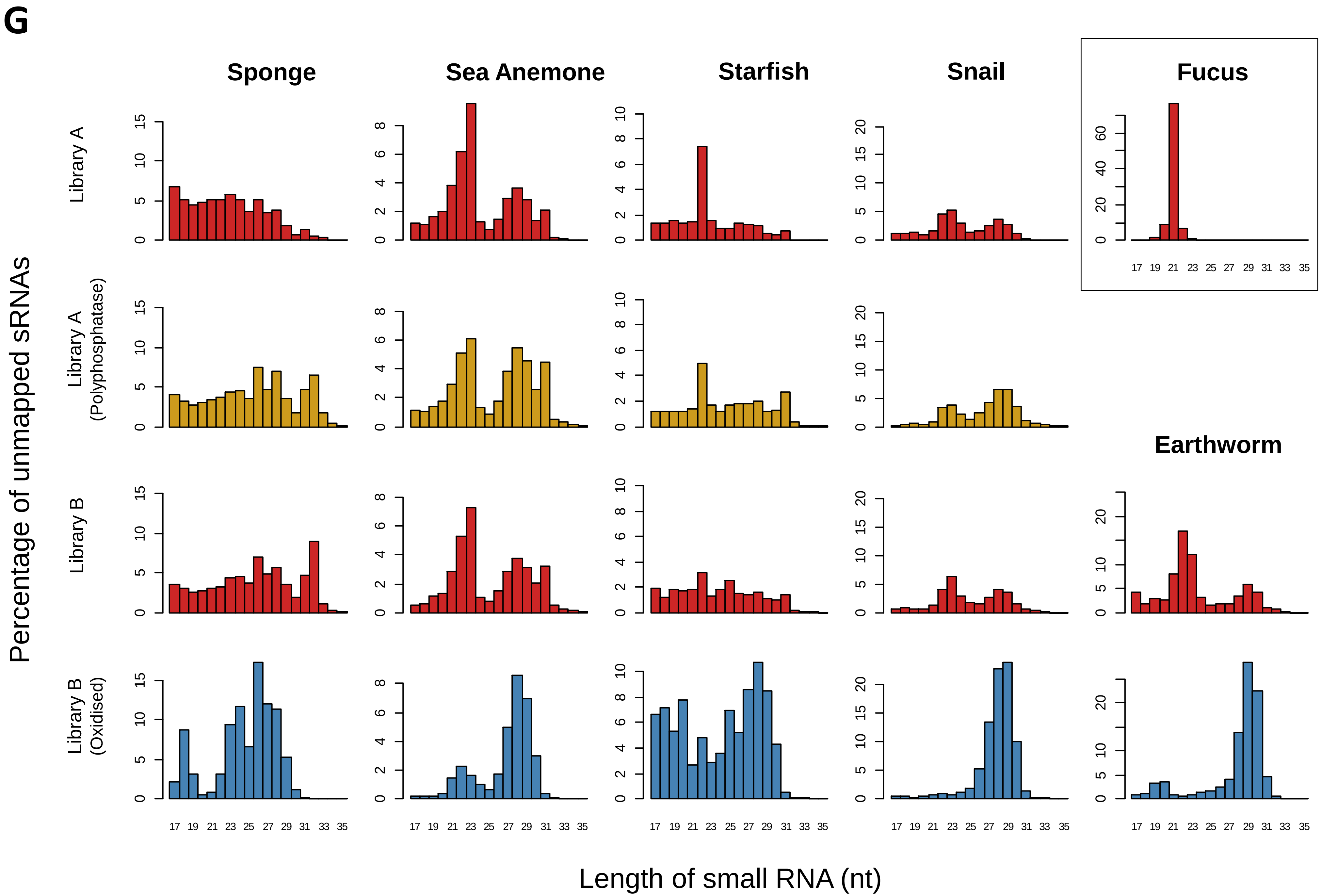
G
Sponge
Sea Anemone
Starfish
Snail
Fucus
Earthworm
Library A
Library A
(Polyphosphatase)
Library B
Library B
(Oxidised)
Percentage of unmapped sRNAs
Length of small RNA (nt)
