# Supplementary material for: Metagenomic sequencing suggests a diversity of RNA interference-like responses to viruses across multicellular eukaryotes
Source: PLoS Genet. 2018 Jul 30;14(7):e1007533. doi: 10.1371/journal.pgen.1007533 (PMC6085071; doi:10.1371/journal.pgen.1007533)

A: Barns Ness dog whelk orthomyxo−like virus 1 PA

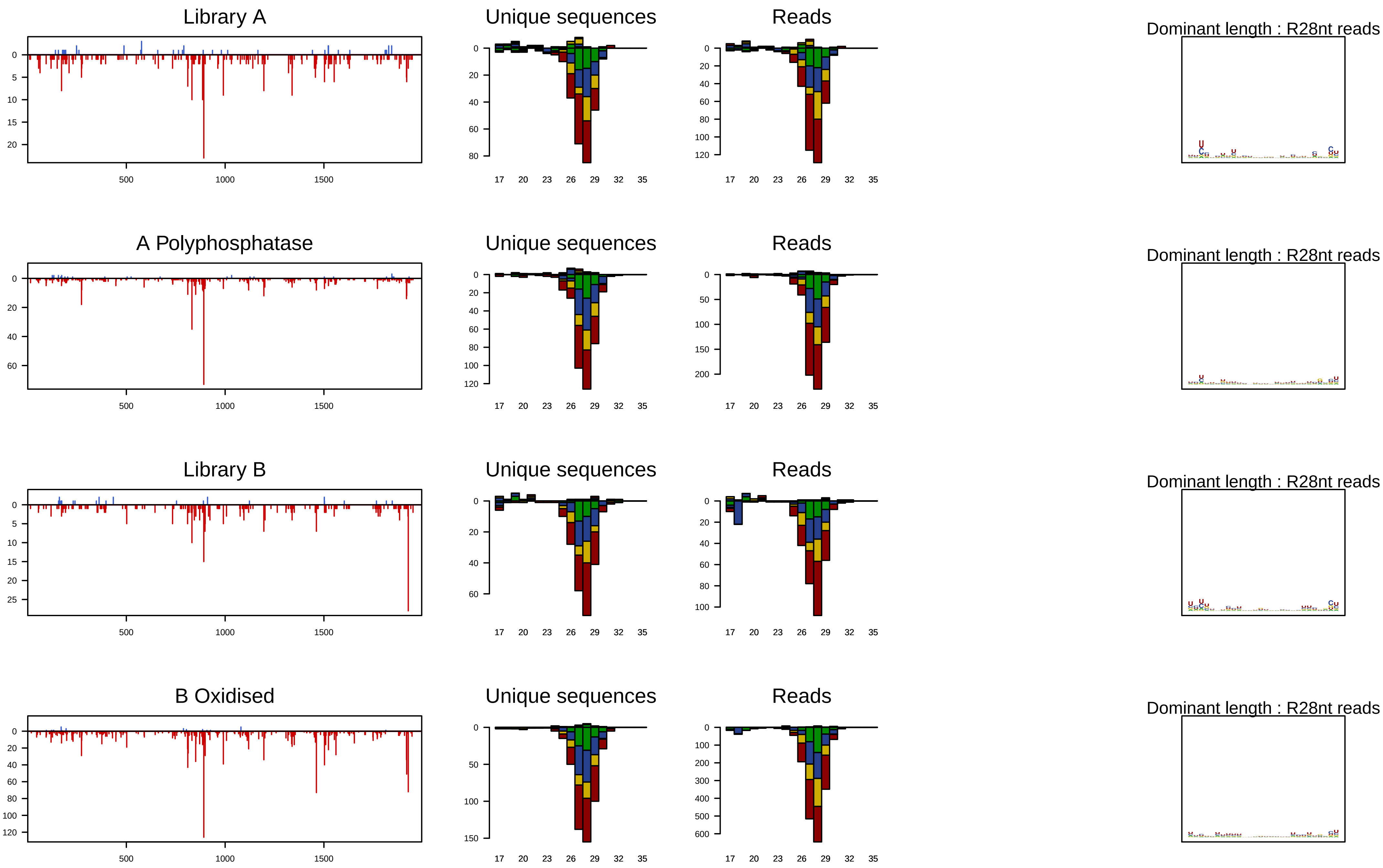

B: Barns Ness dog whelk orthomyxo−like virus 1 PB1

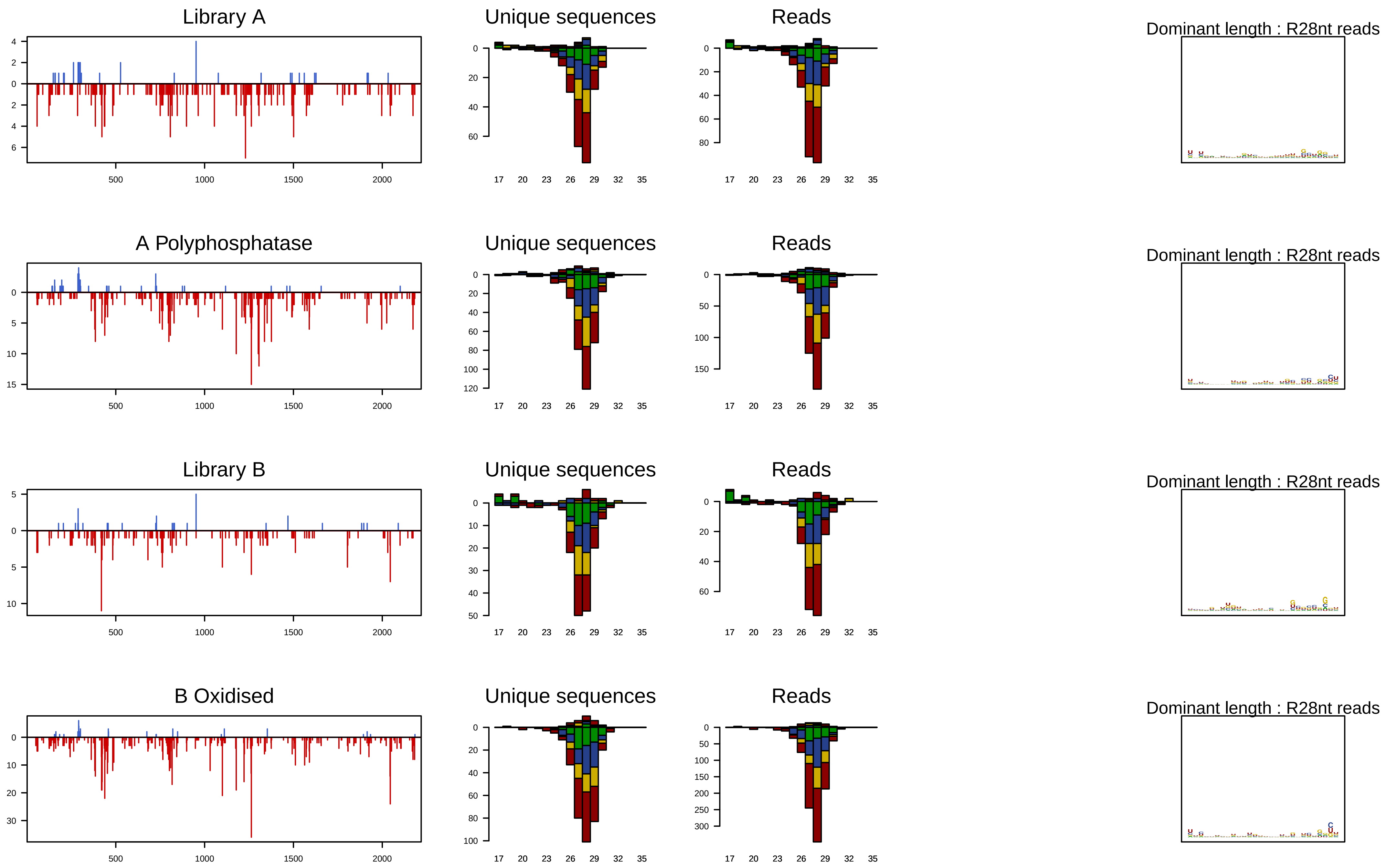

C: Caledonia dog whelk rhabdo−like virus 1

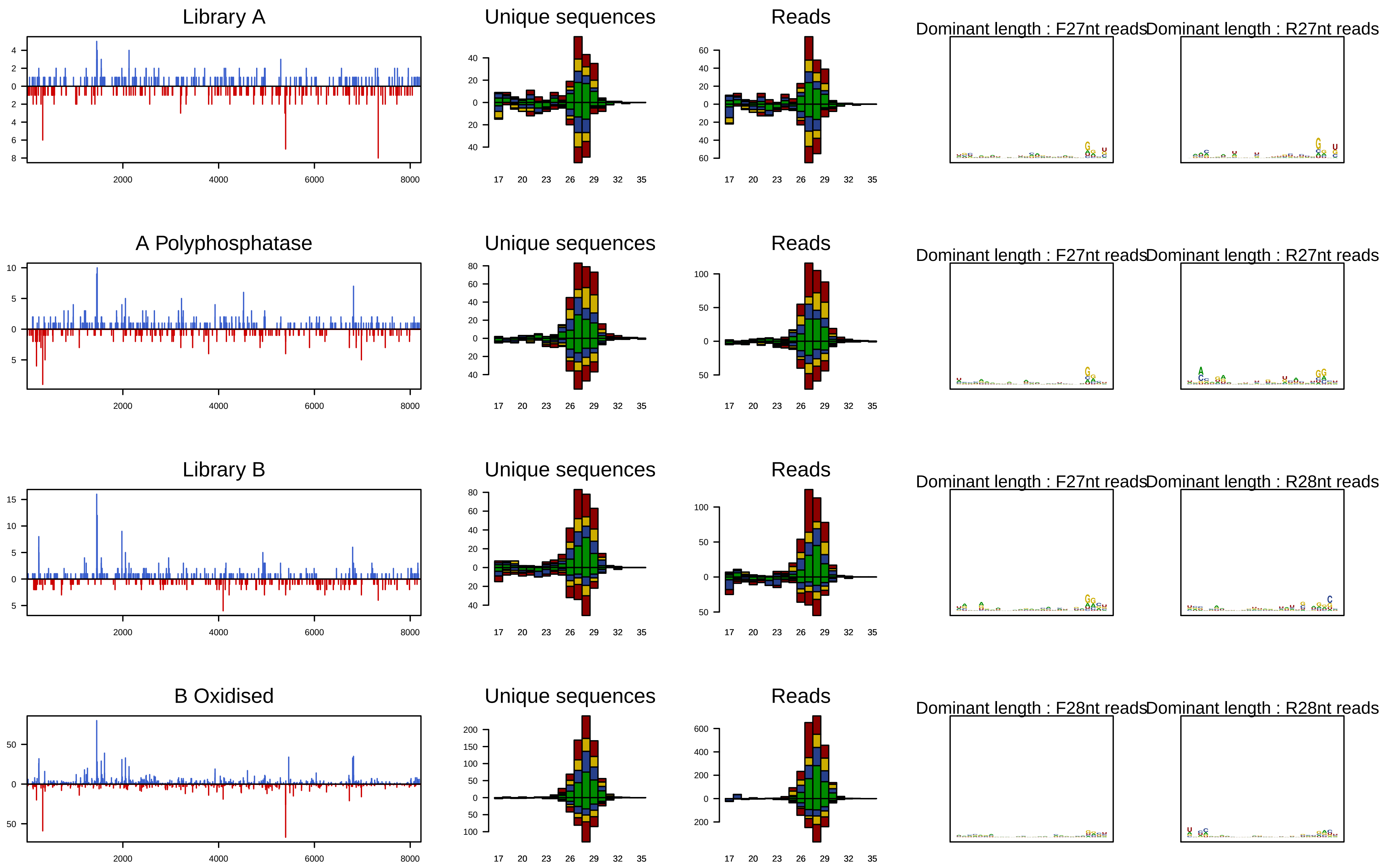

D: Caledonia dog whelk rhabdo−like virus 2

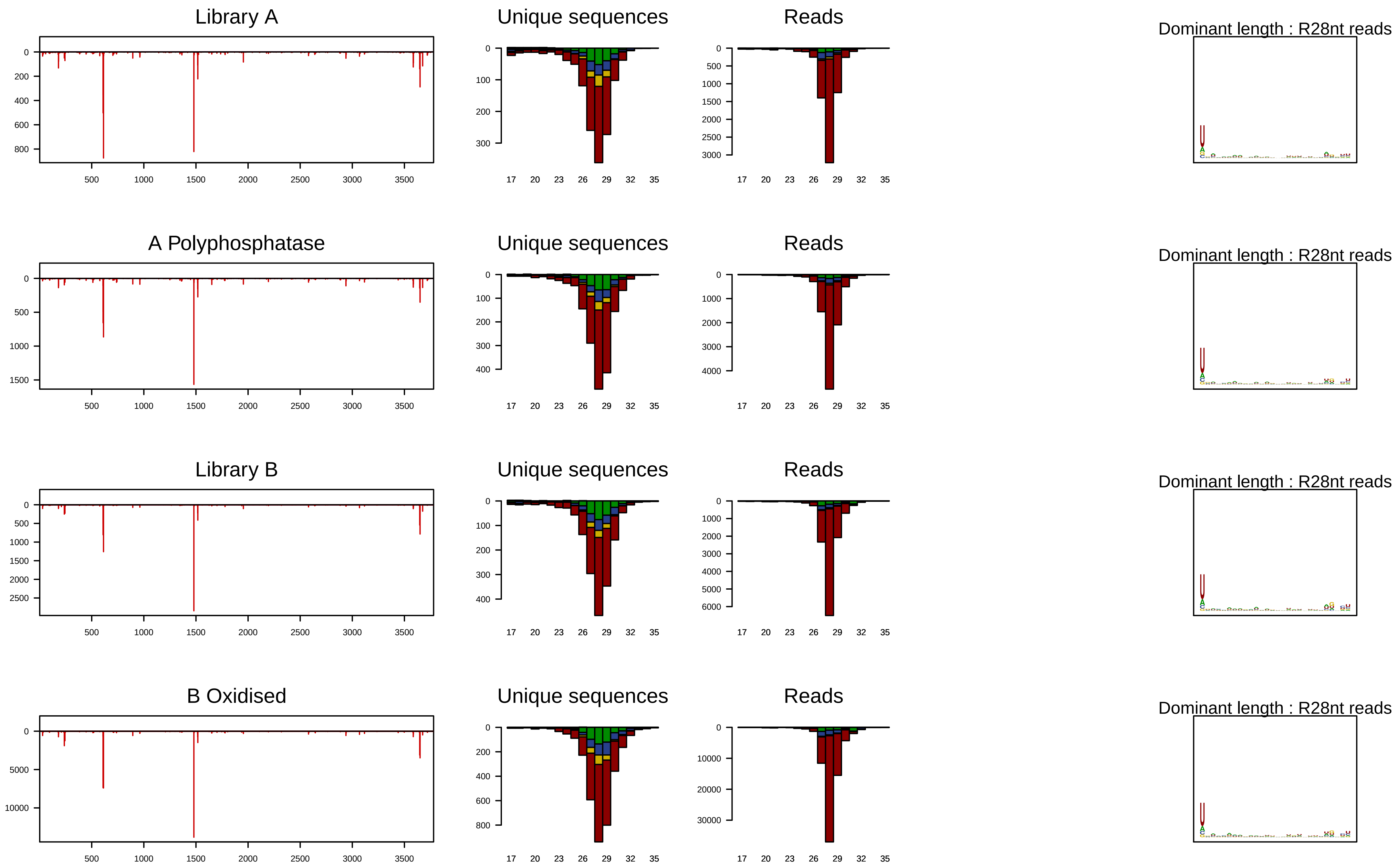

E: Caledonia starfish parvo−like virus 1

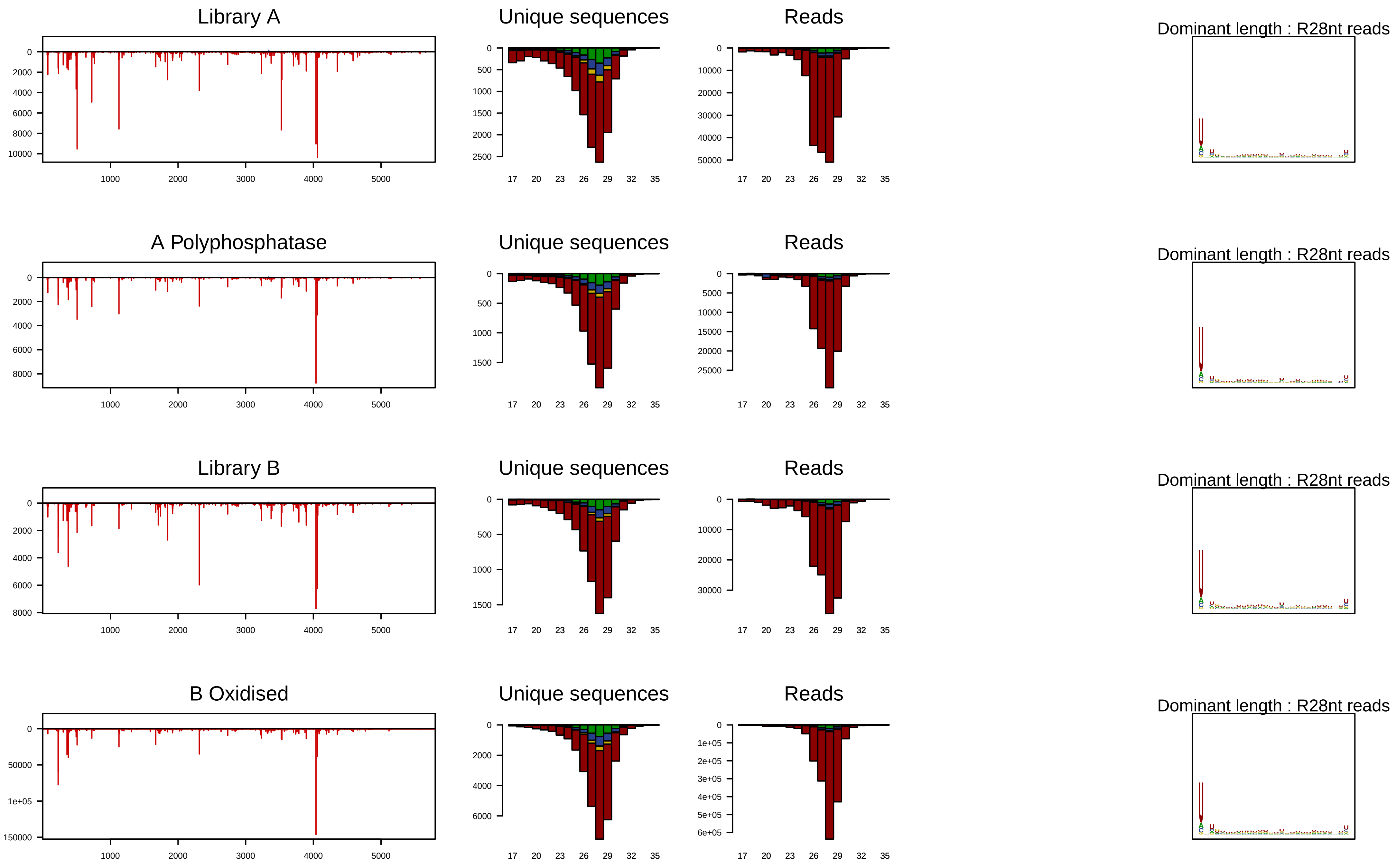

F: Caledonia starfish parvo−like virus 2

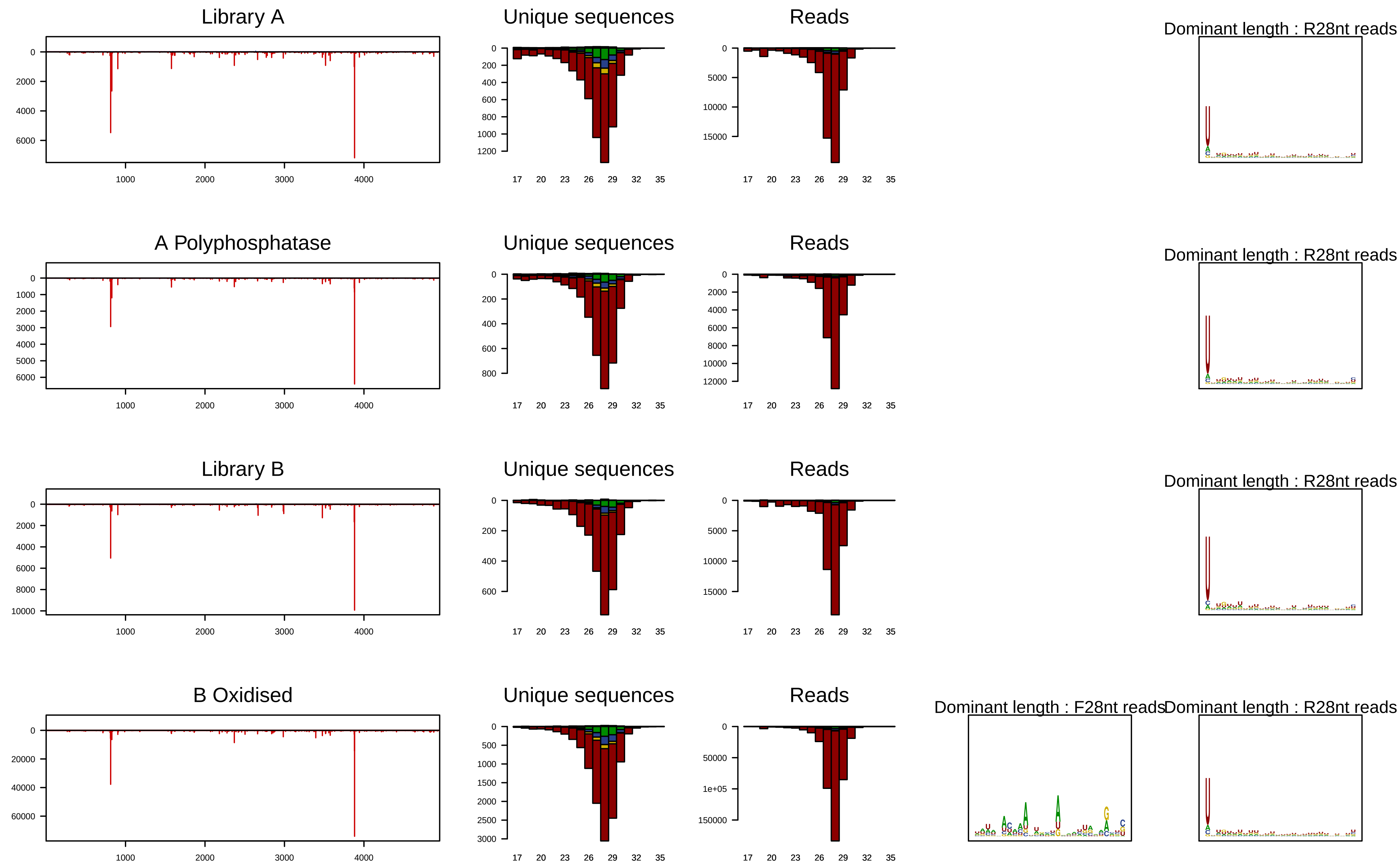

G: Caledonia starfish parvo−like virus 3

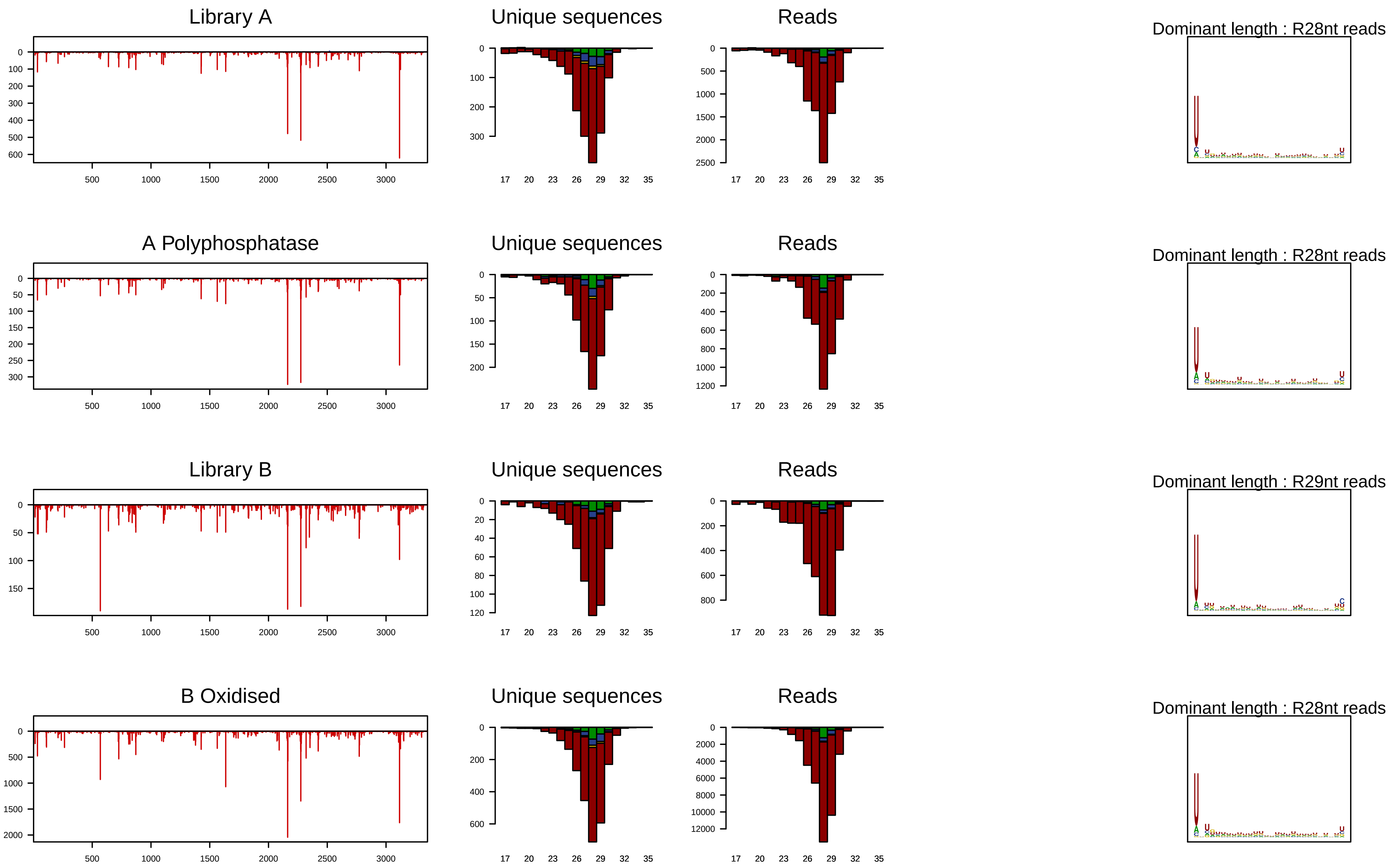

H: Millport starfish parvo−like virus 1

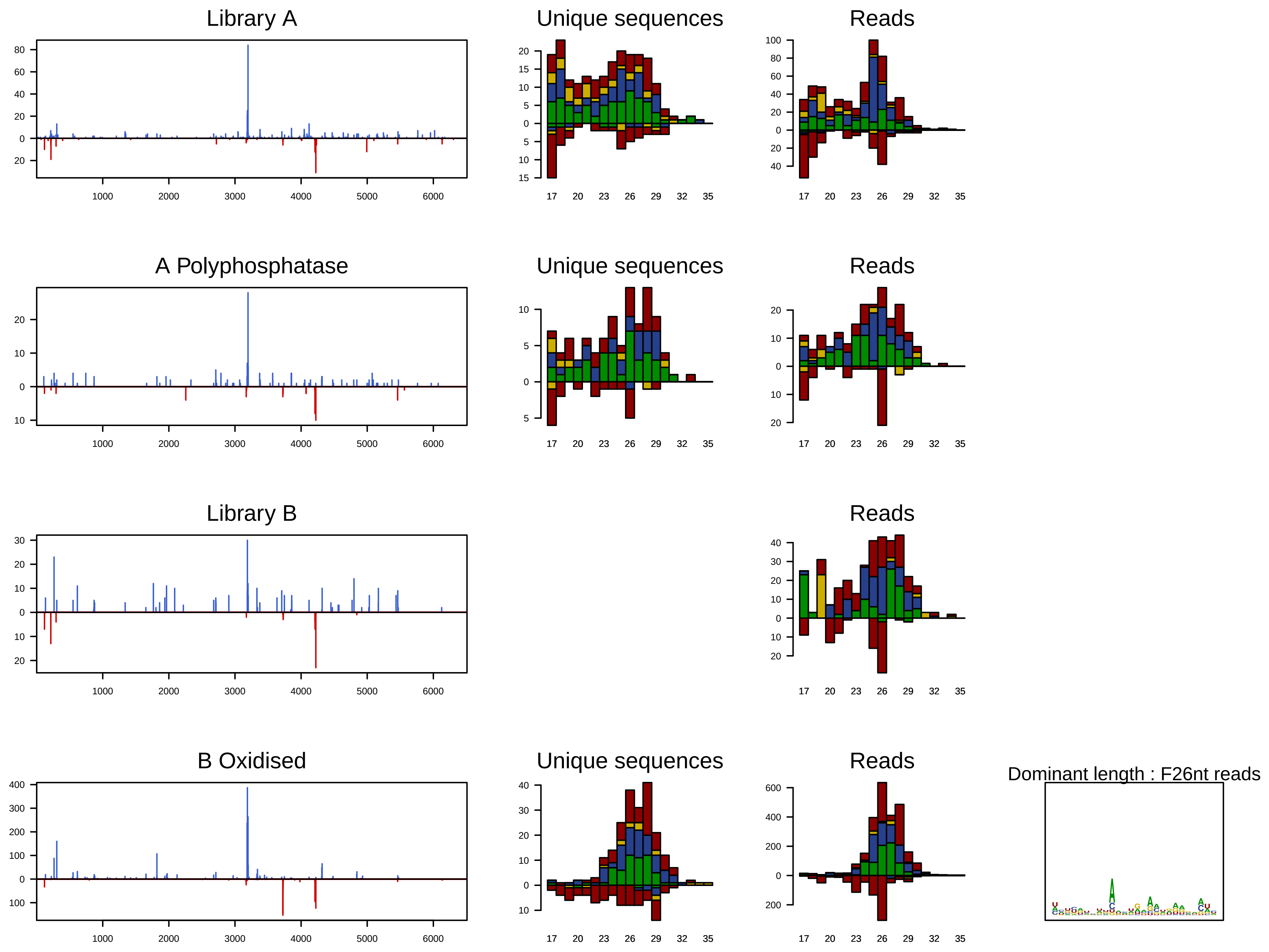

I: Caledonia beadlet anemone parvo−like virus 1

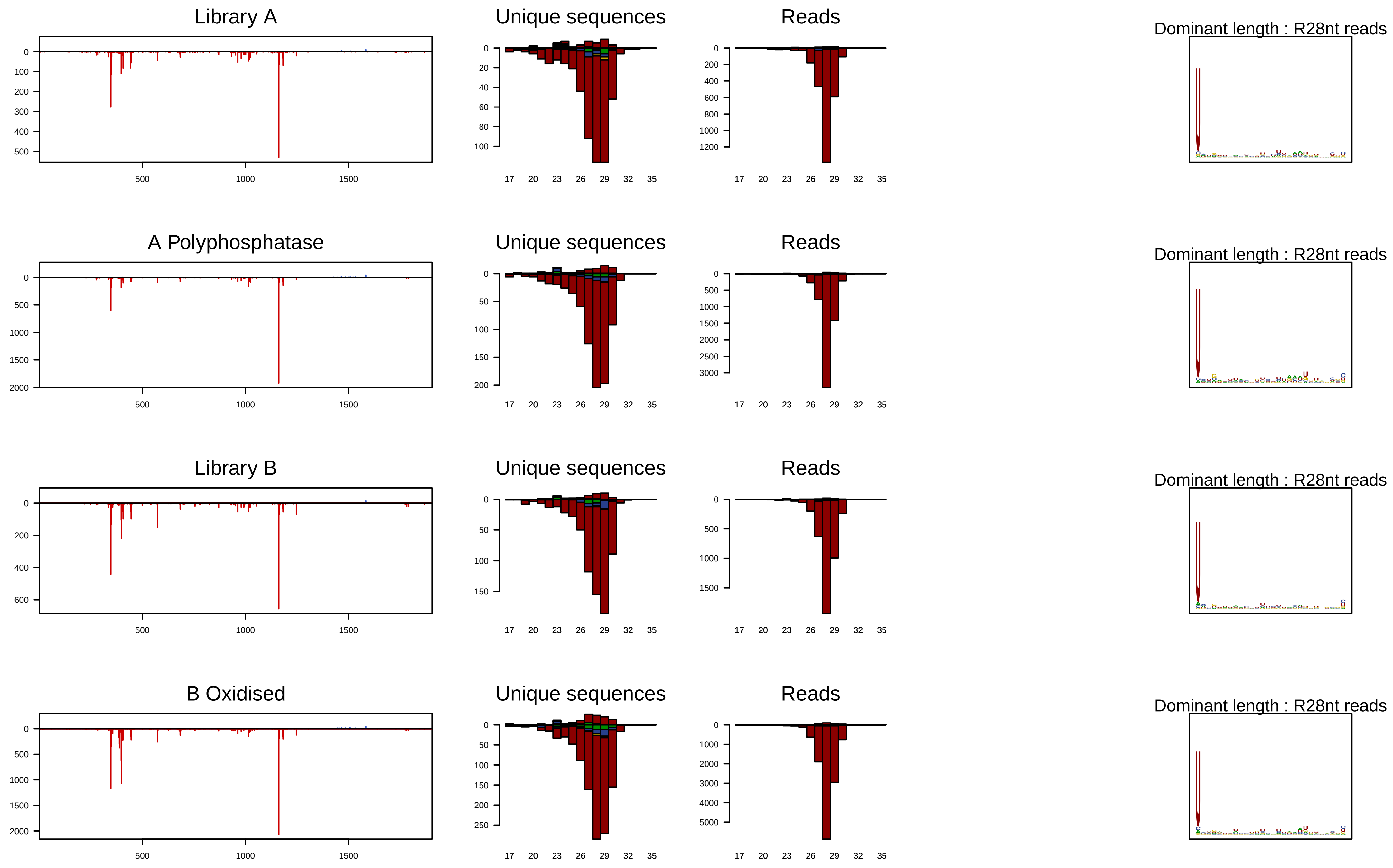

J: Barns Ness serrated wrack bunya/phlebo−like virus 1

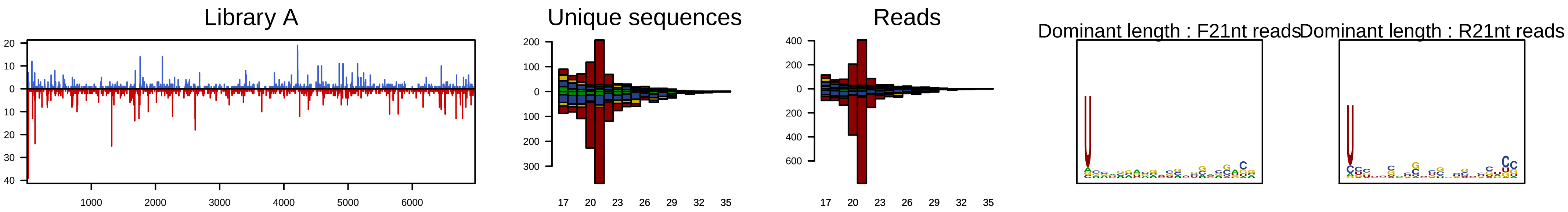

Supplement: S6 Fig — Panels A-D are dog-whelk RNA viruses, panels E-H starfish DNA virus-like contigs, panel I is the anemone DNA virus, and panel J is the brown alga virus (note that only one library was made for this sample). In each panel, rows (top to bottom) represent each library: Library A, polyphosphatase-treated library A, Library B, and oxidised library B. Columns (left to right) are (i) Origin of reads from each genome position (red lines above the x-axis denote reads from the positive sense strand, blue lines below the x-axis denote reads from the negative sense strand; (ii) Bar plot of frequencies of unique sequences, bars above the x-axis denote reads from the positive sense strand, those below the x-axis denote reads from the negative sense strand, colours indicate 5' base (U red, G yellow, C blue and A green); (iii) Barplot of frequencies of reads; (iv) Sequence logo for the unique sequences of the most frequent length deriving from the positive strand; (v) Sequence logo for the unique sequences of the most frequent length deriving from the negative strand. The data required to plot the size distributions are provided in S5 Table. (PDF) [file pgen.1007533.s006.pdf]
