# Supplementary material for: Metagenomic sequencing suggests a diversity of RNA interference-like responses to viruses across multicellular eukaryotes
Source: PLoS Genet. 2018 Jul 30;14(7):e1007533. doi: 10.1371/journal.pgen.1007533 (PMC6085071; doi:10.1371/journal.pgen.1007533)
Supplement: S7 Fig — Columns show (left) the number and size distribution of small RNAs that mapped to contigs provisionally classified as bacterial by similarity search, and the hotspots and size distributions for the nominal bacterial contig that displayed the largest (centre) and second-largest (right) number of small RNA mappings. The combined oxidised libraries are shown for all species except the brown alga (untreated library, as no oxidised library was prepared), and colours and axes are as in Figs 3–5. Read numbers were very small for the Starfish, Dog Whelk and Sea Anemone (<2000 reads) and in Fucus the vast majority of small RNAs derived from a bacterial rRNA, but their size distribution suggests that they represent degradation products. Those in the Earthworm and the Sponge strongly resemble host primary piRNAs in their strand bias, size distribution and base composition (compare with Fig 5), but manual inspection suggests that almost all derived from misclassified host TE contigs. (PDF) [file pgen.1007533.s007.pdf]

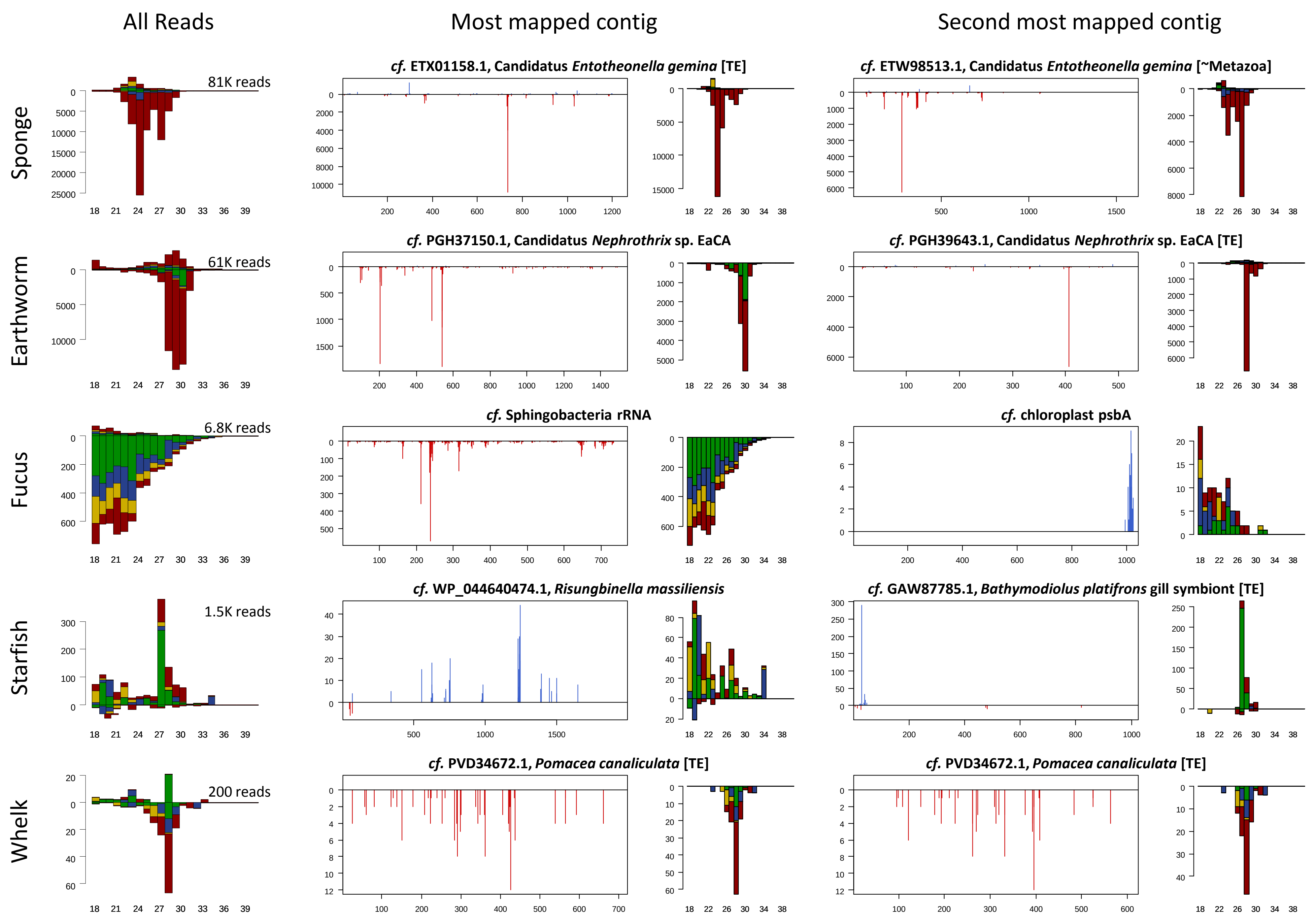

All Reads
Most mapped contig
Second most mapped contig
Sponge
81K reads
cf. ETX01158.1, Candidatus Entotheonella gemina [TE]
cf. ETW98513.1, Candidatus Entotheonella gemina [~Metazoa]
Earthworm
61K reads
cf. PGH37150.1, Candidatus Nephrothrix sp. EaCA
cf. PGH39643.1, Candidatus Nephrothrix sp. EaCA [TE]
Fucus
6.8K reads
cf. Sphingobacteria rRNA
cf. chloroplast psbA
Starfish
1.5K reads
cf. WP_044640474.1, Risungbinella massiliensis
cf. GAW87785.1, Bathymodiolus platifrons gill symbiont [TE]
Whelk
200 reads
cf. PVD34672.1, Pomacea canaliculata [TE]
cf. PVD34672.1, Pomacea canaliculata [TE]
