# Supplementary material for: Metagenomic sequencing suggests a diversity of RNA interference-like responses to viruses across multicellular eukaryotes
Source: PLoS Genet. 2018 Jul 30;14(7):e1007533. doi: 10.1371/journal.pgen.1007533 (PMC6085071; doi:10.1371/journal.pgen.1007533)

A: Starfish DN88845 cf. DIRS−1 CGi DIRS Crassostrea gigas

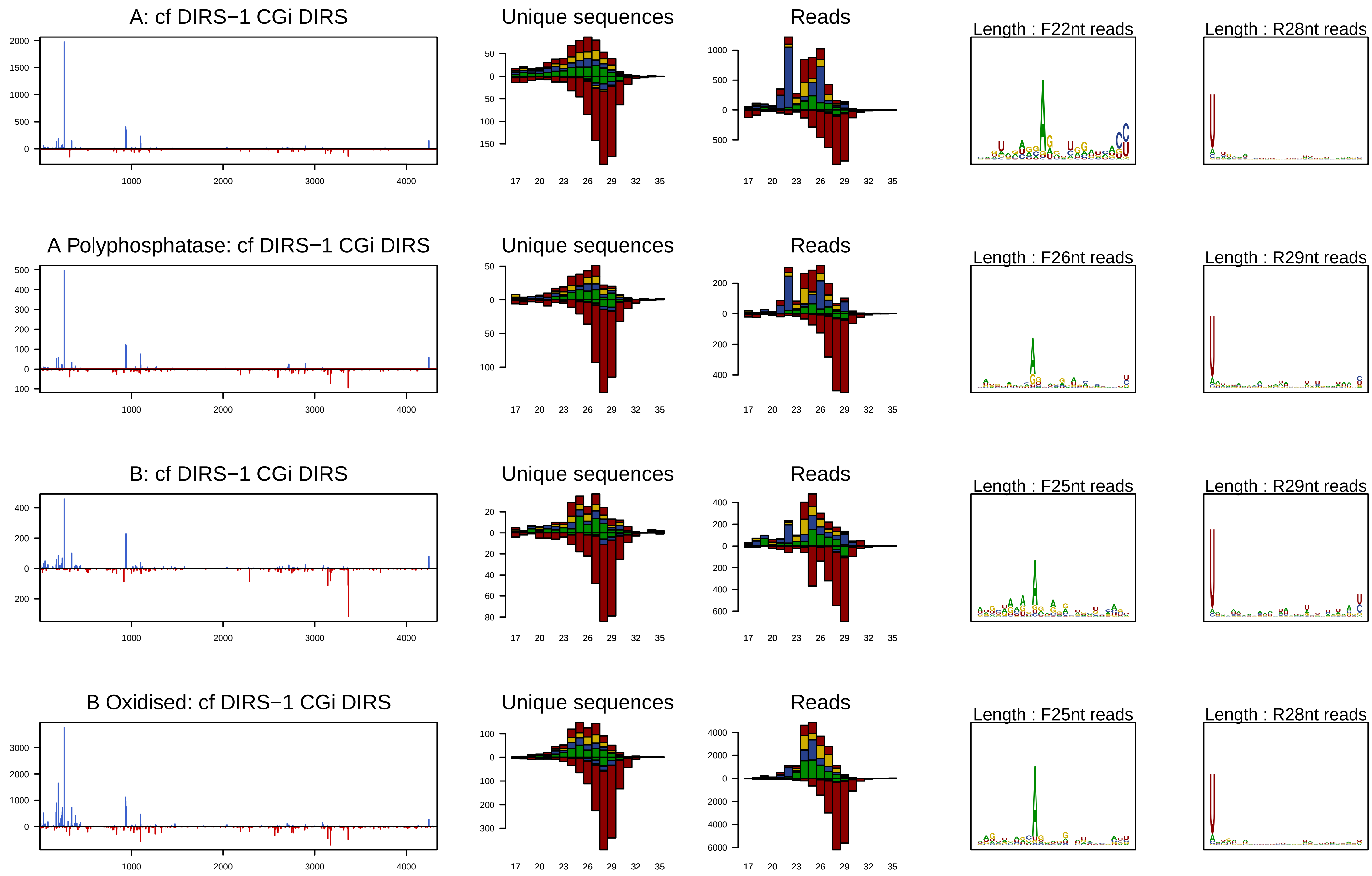

B: Starfish DN89380 cf. Sola2−1 CGi Sola Crassostrea gigas

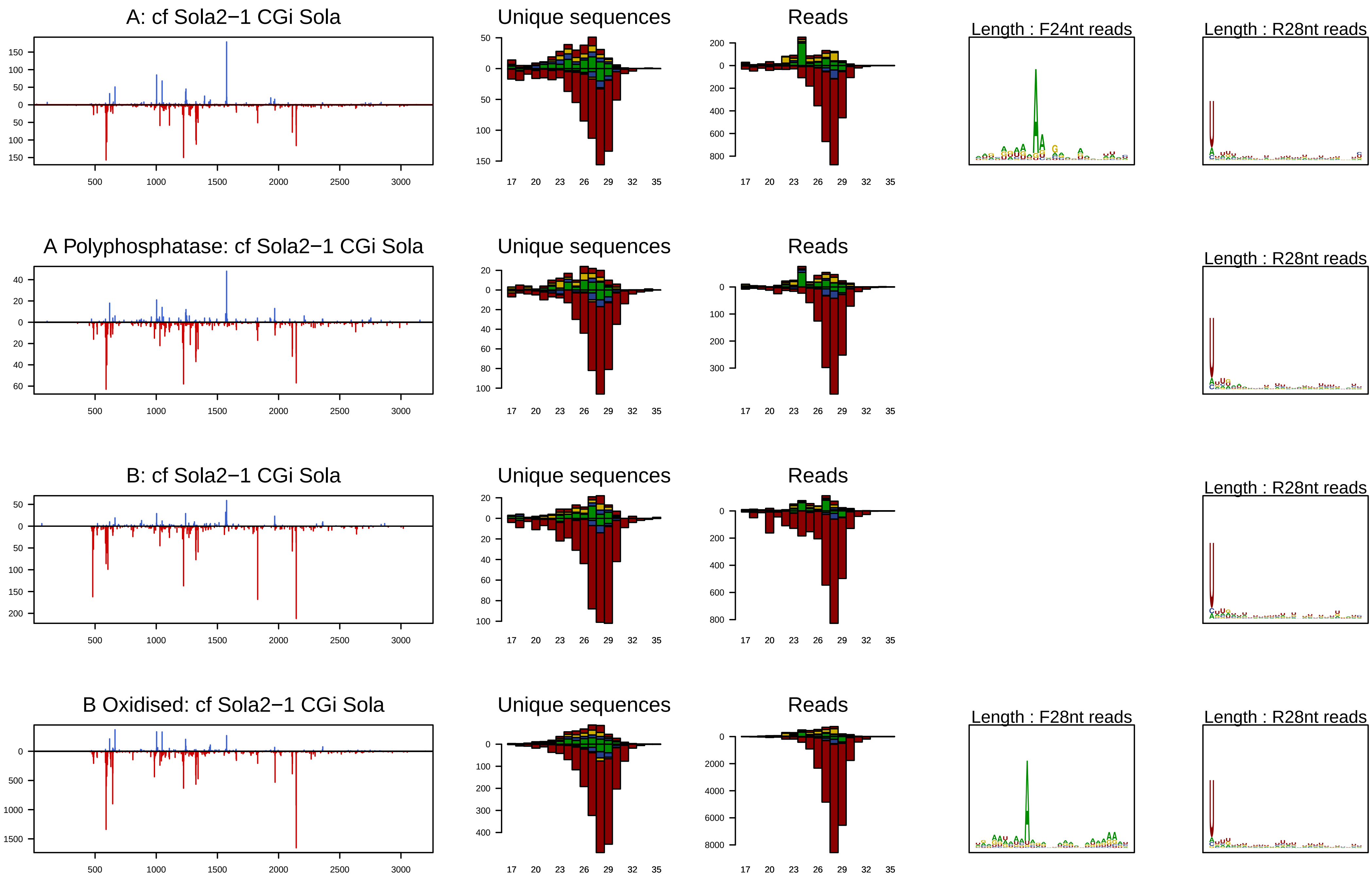

C: Starfish DN88622 cf. RTE−4 CTe RTE Capitella teleta

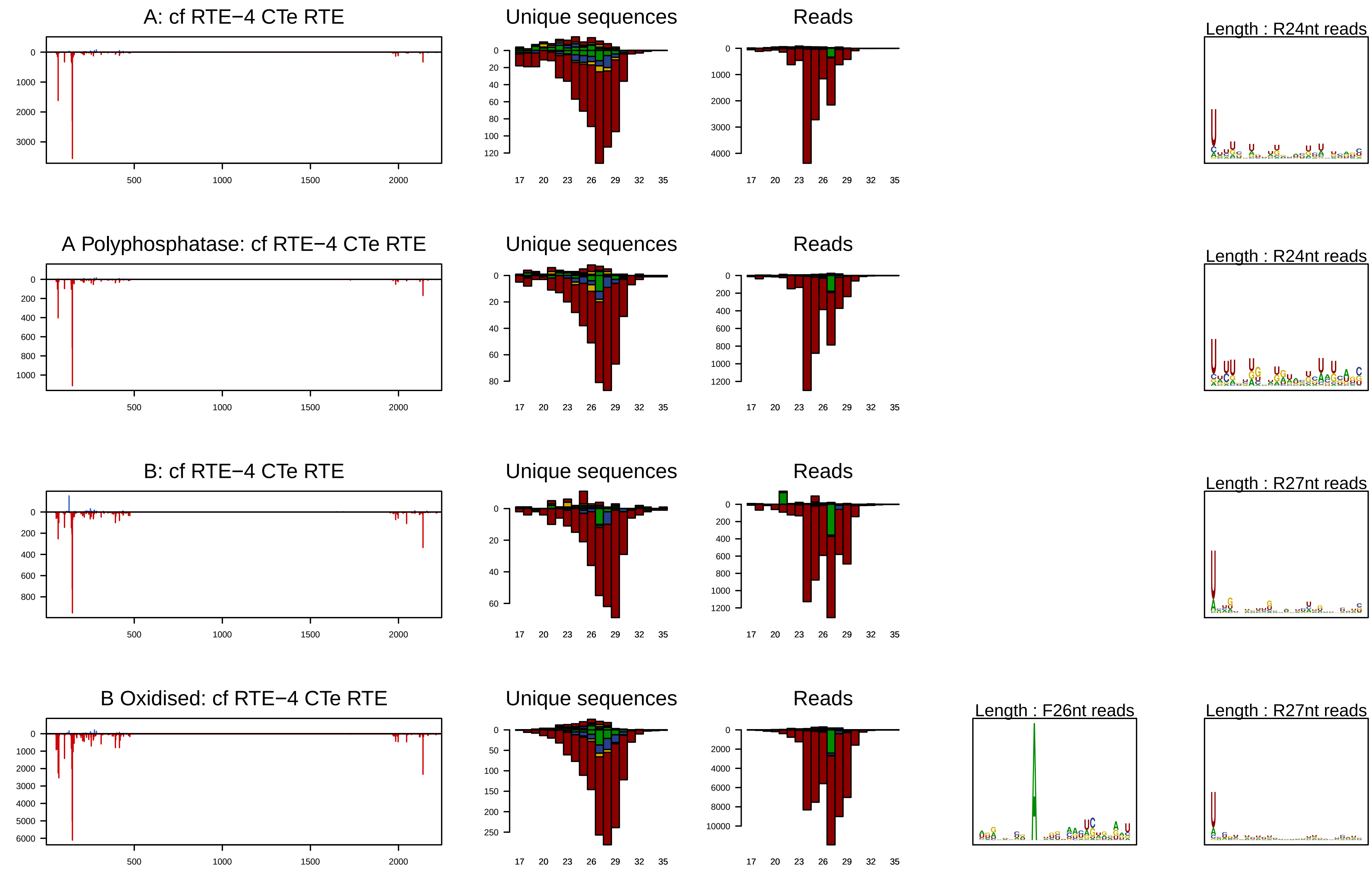

D: Dog Whelk DN75842 cf. Gypsy−35 CGi−I Gypsy Crassostrea gigas

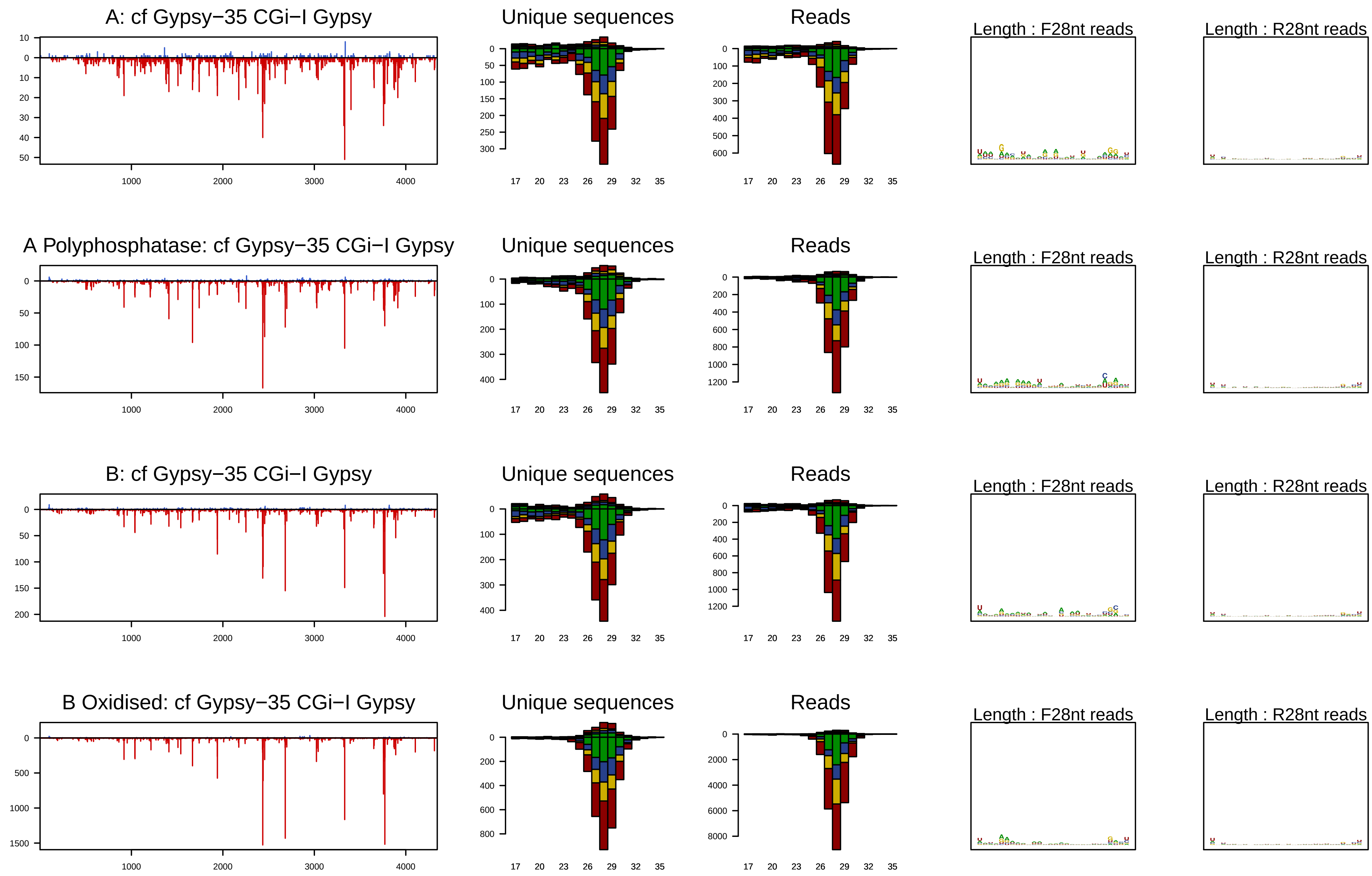

E: Dog Whelk DN73078 cf. Academ−3 SP Academ Strongylocentrotus purpuratus

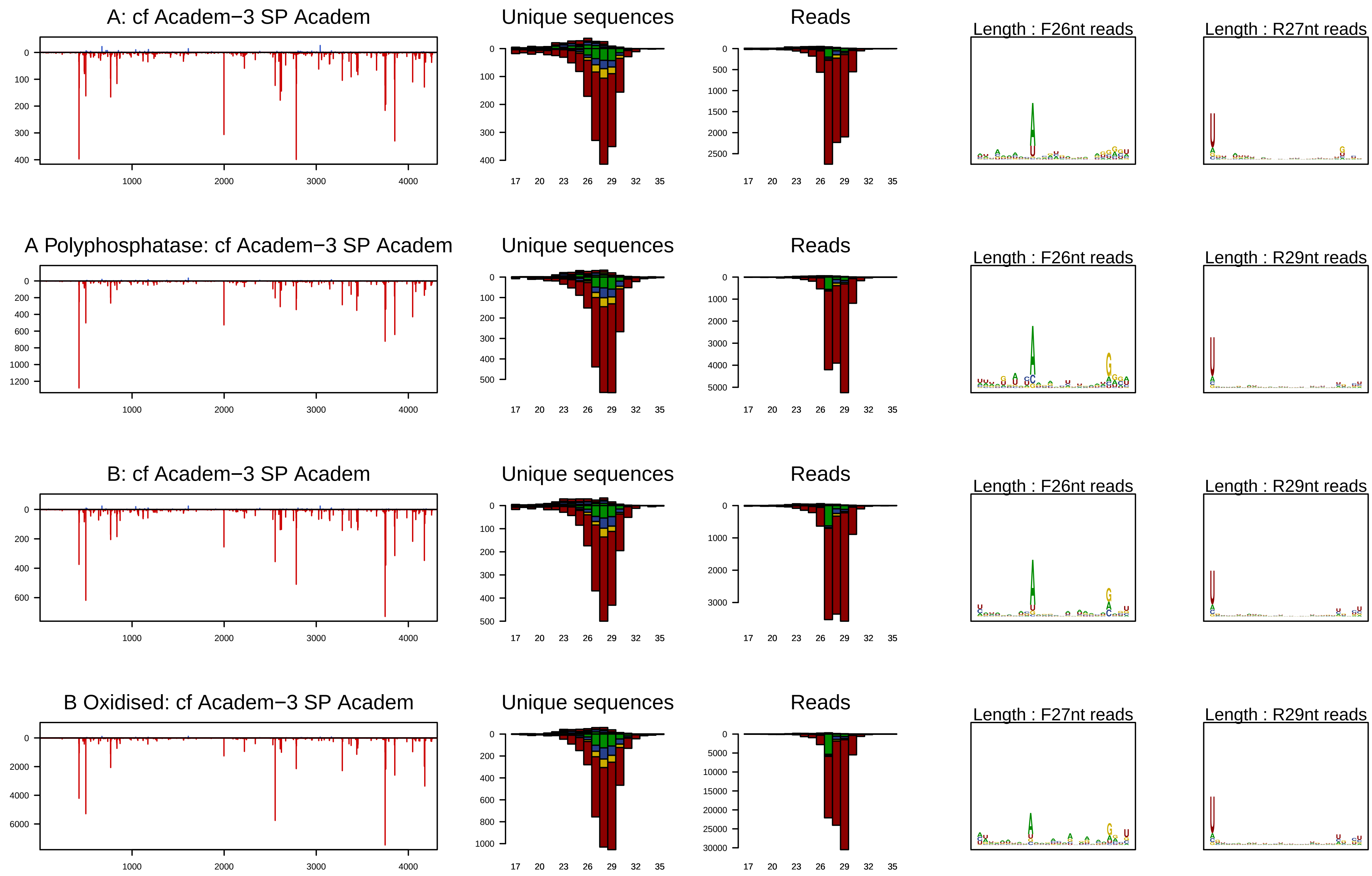

F: Dog Whelk DN58439 cf. Mariner−25 HM Mariner/Tc1 Hydra vulgaris

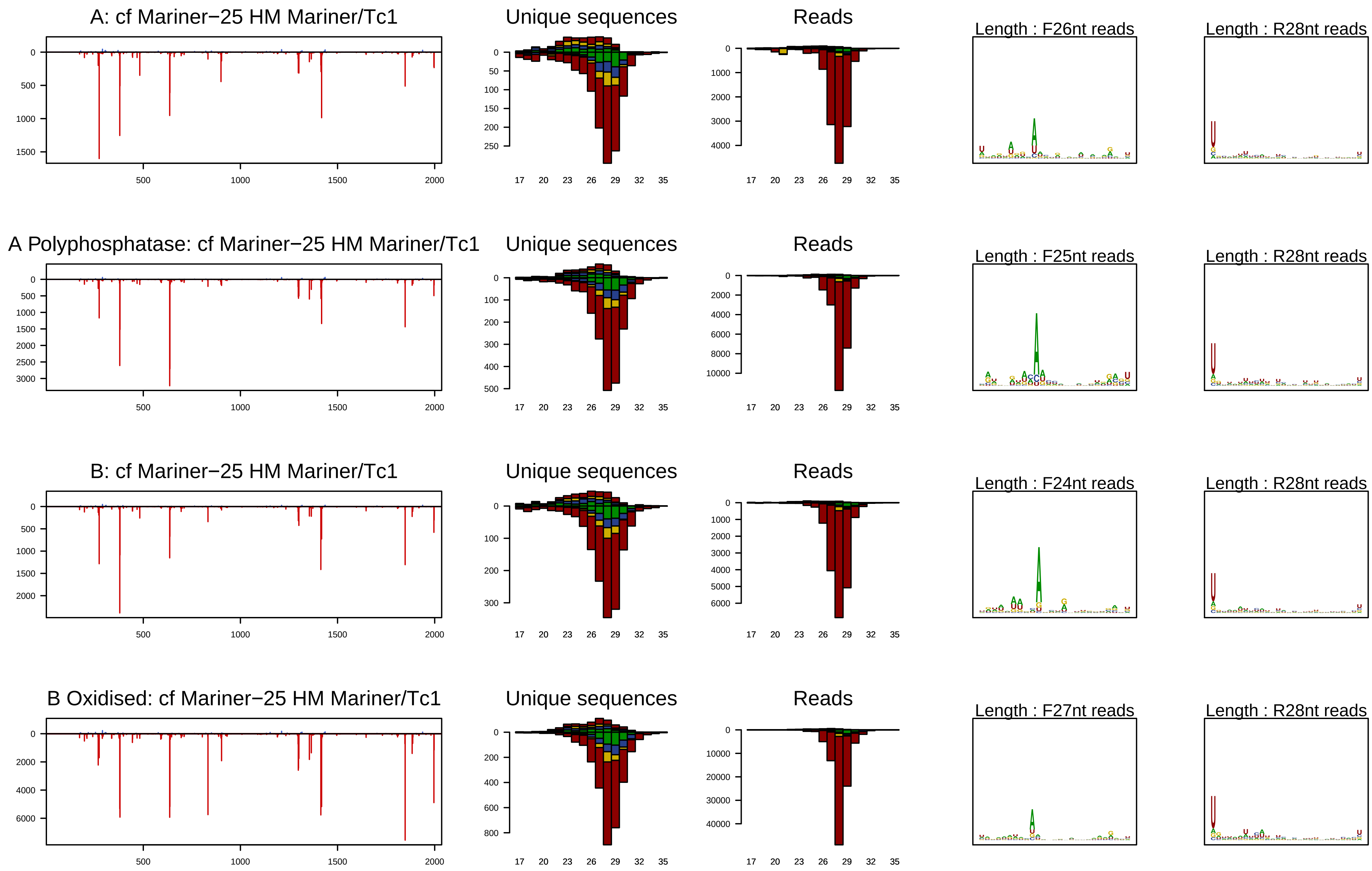

G: Sponge DN39216 cf. Gypsy−22−I BF

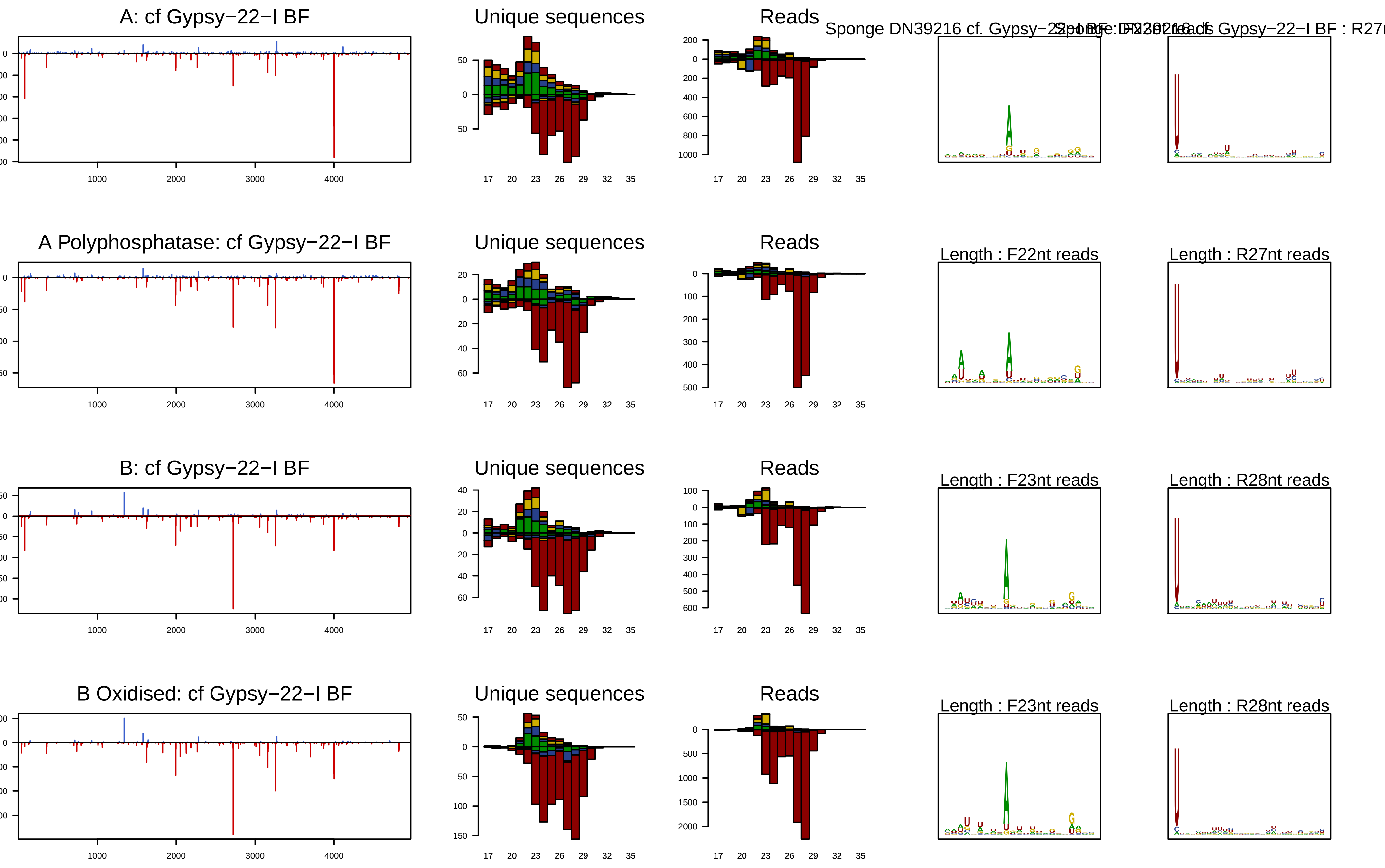

H: Sponge DN36385 cf. Gypsy−6 SSa−I Gypsy Salmo salar

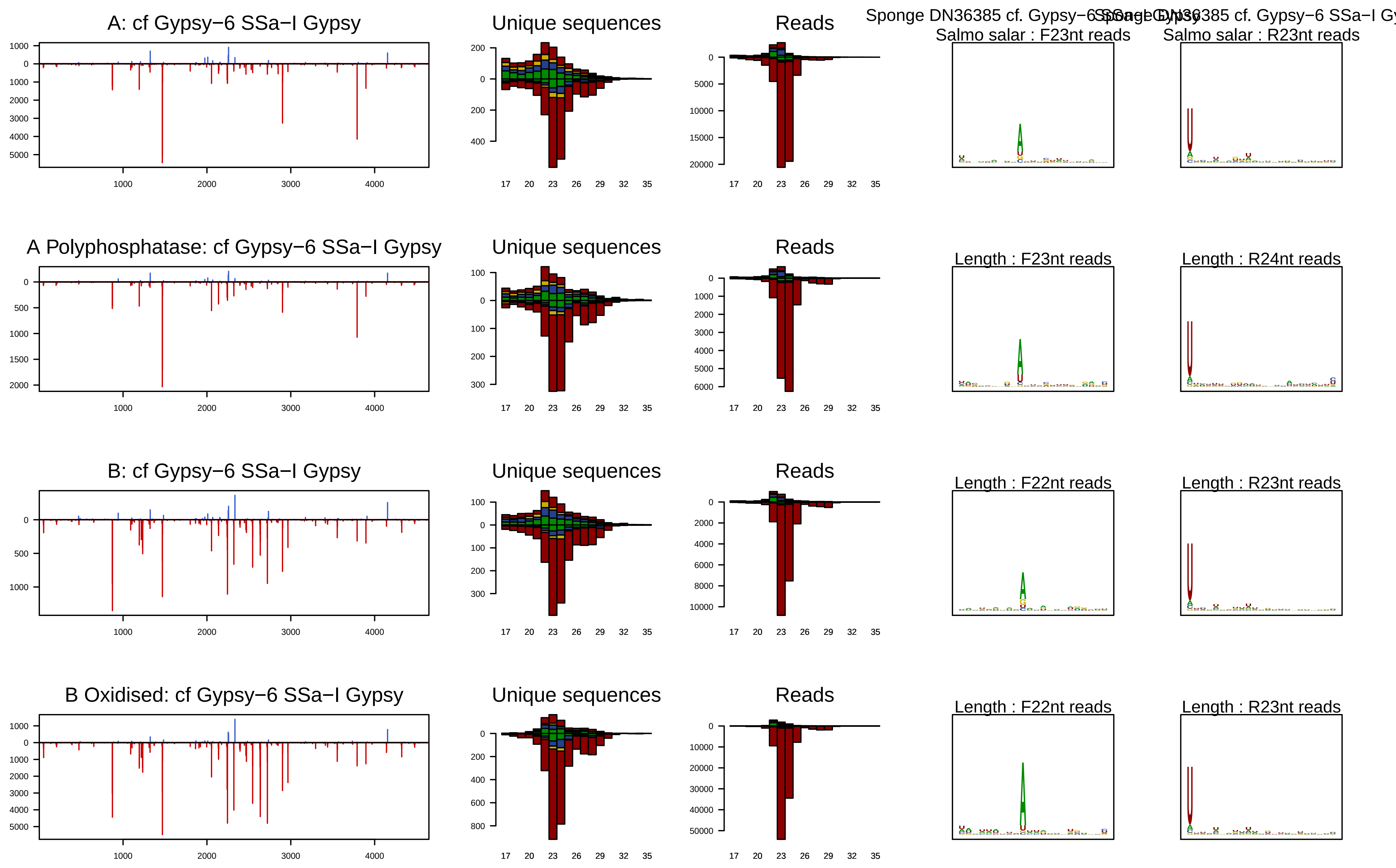

I: Sponge DN30995 cf. Gypsy−30 SSa−I Gypsy Salmo salar

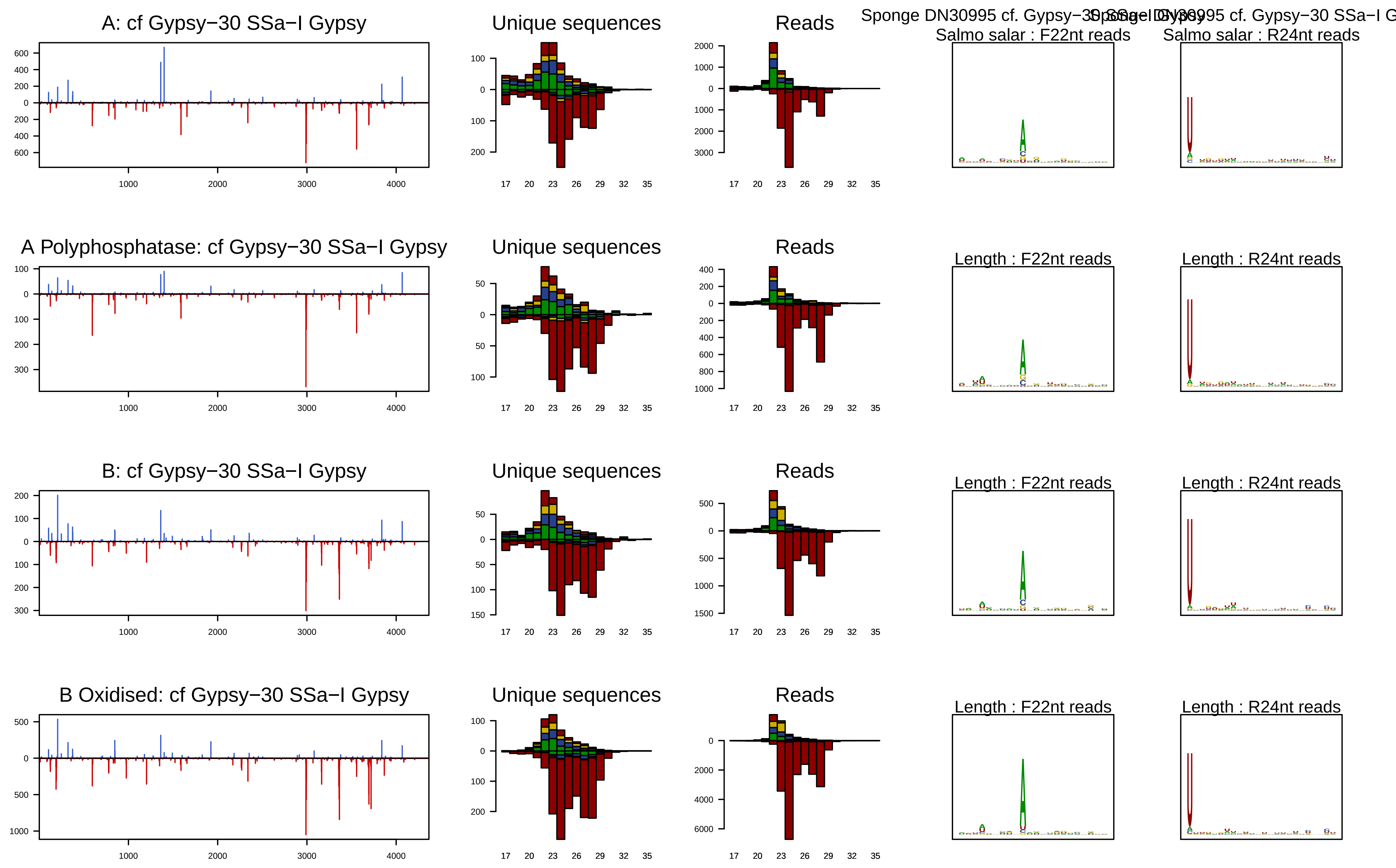

J: Earthworm DN118777 cf. EnSpm−11 DR EnSpm/CACTA Danio rerio

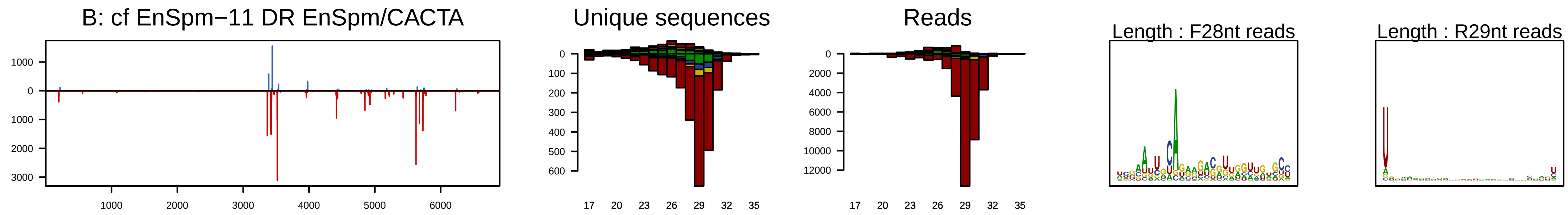

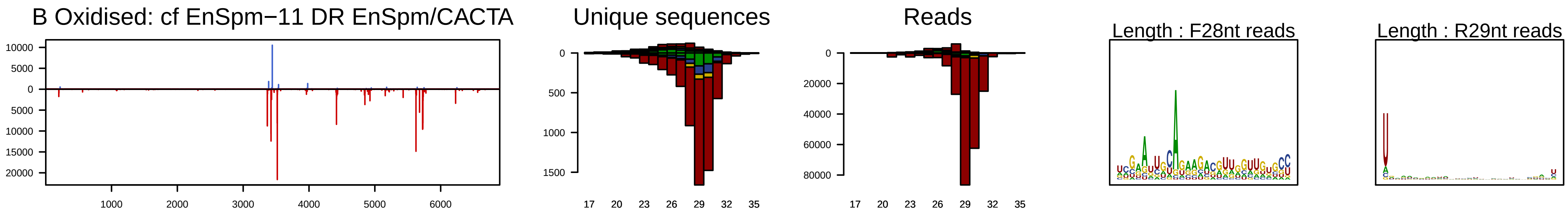

K: Earthworm DN142375 cf. Gypsy−33−I DR Gypsy Danio rerio

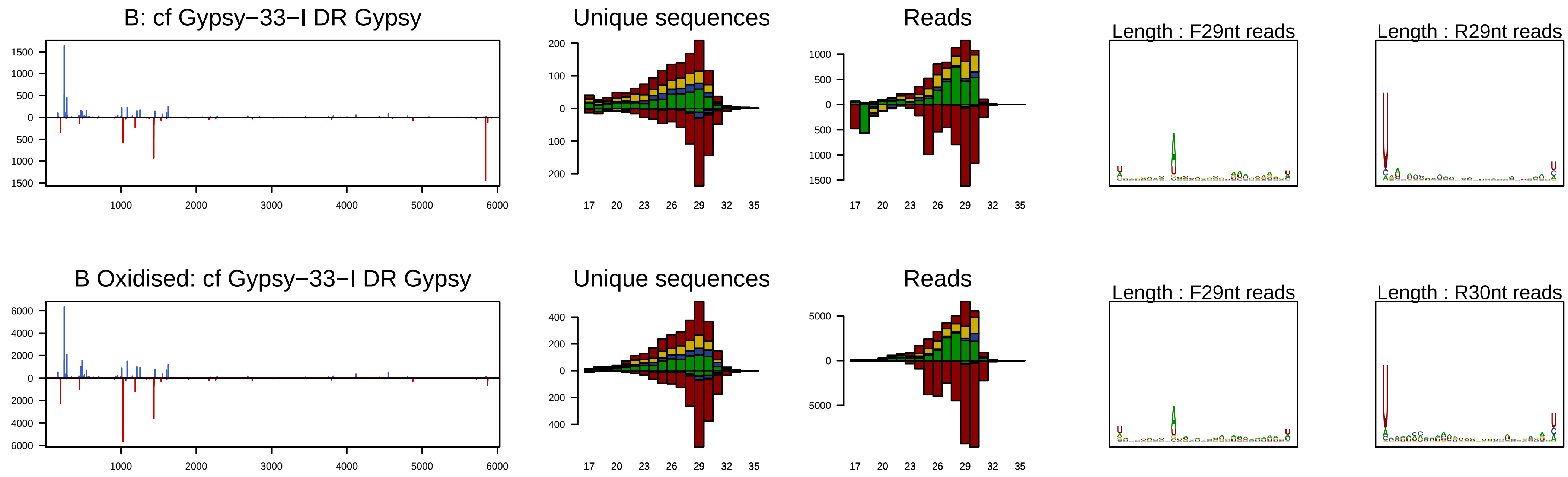

L: Earthworm DN126009 cf. P−38 HMa P Hydra vulgaris

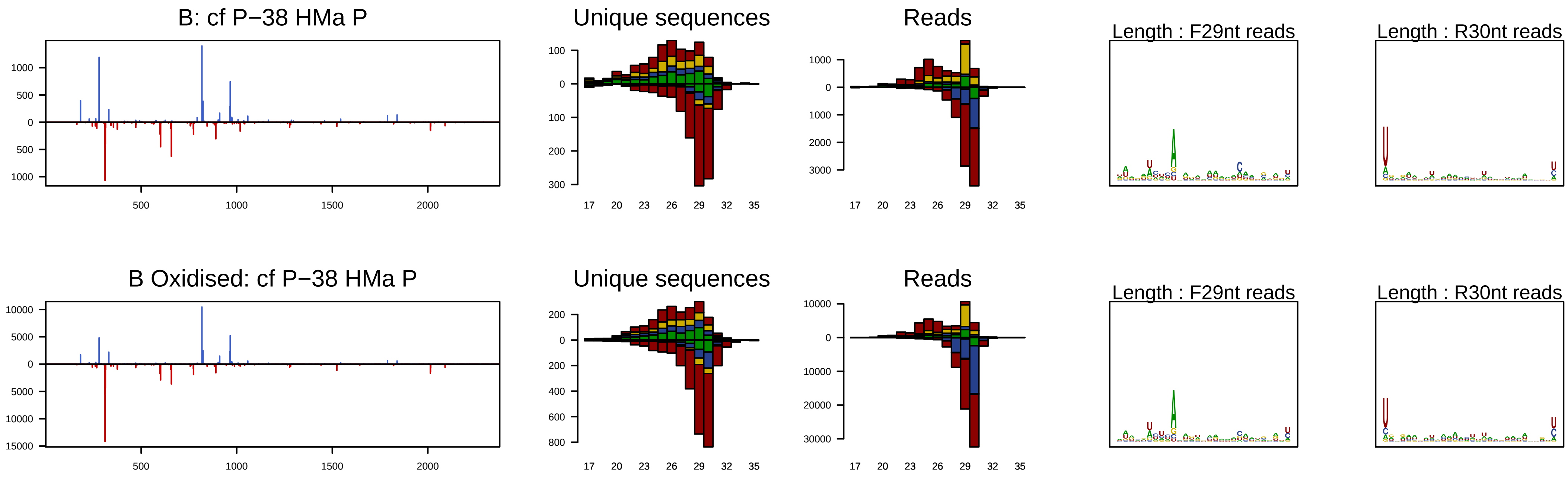

M: Sea Anemone DN77275 cf. Saci−1 INT LTR retrotransposon Schistosoma mansoni

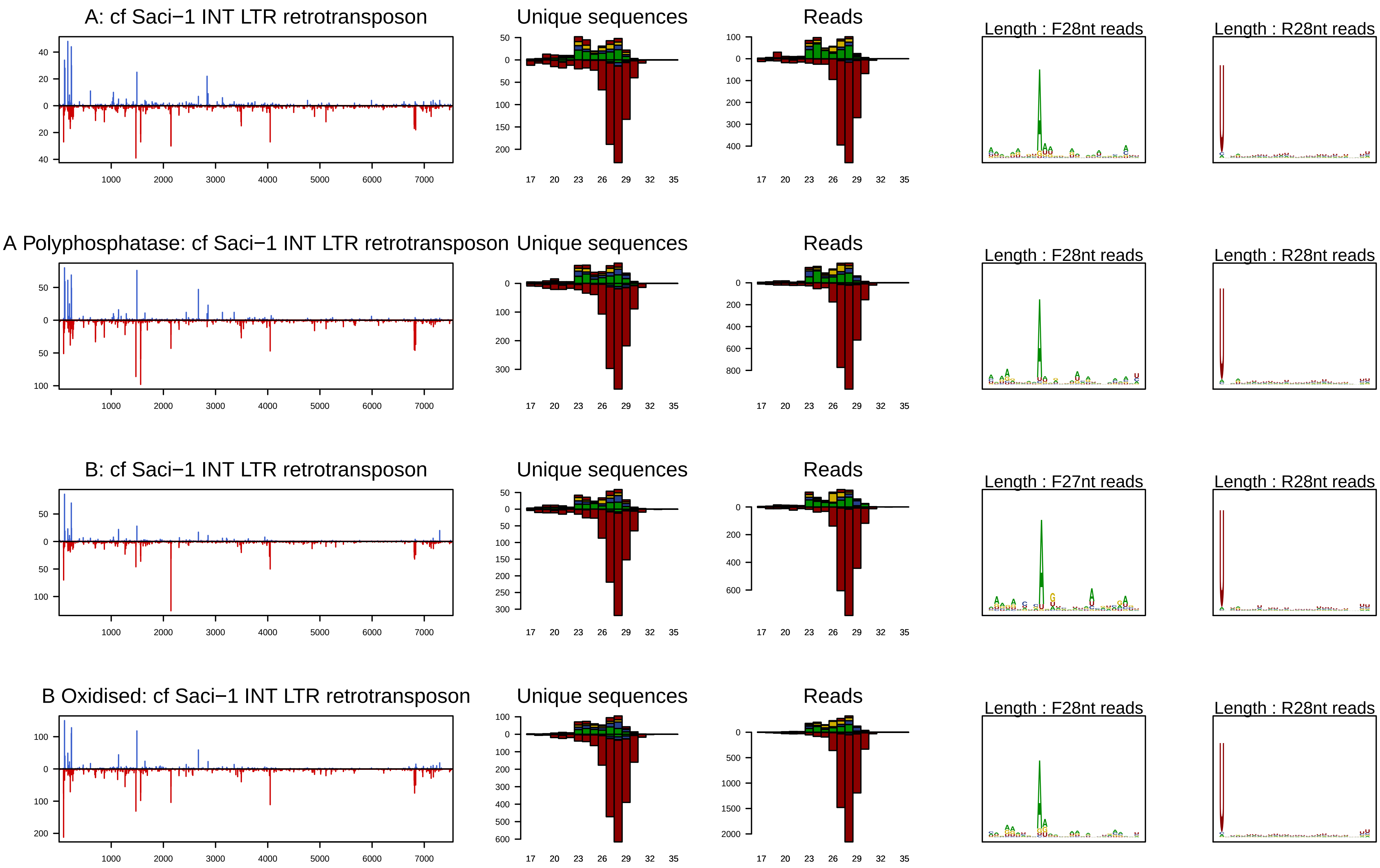

N: Sea Anemone DN78271 cf. Troyka−1−I NV

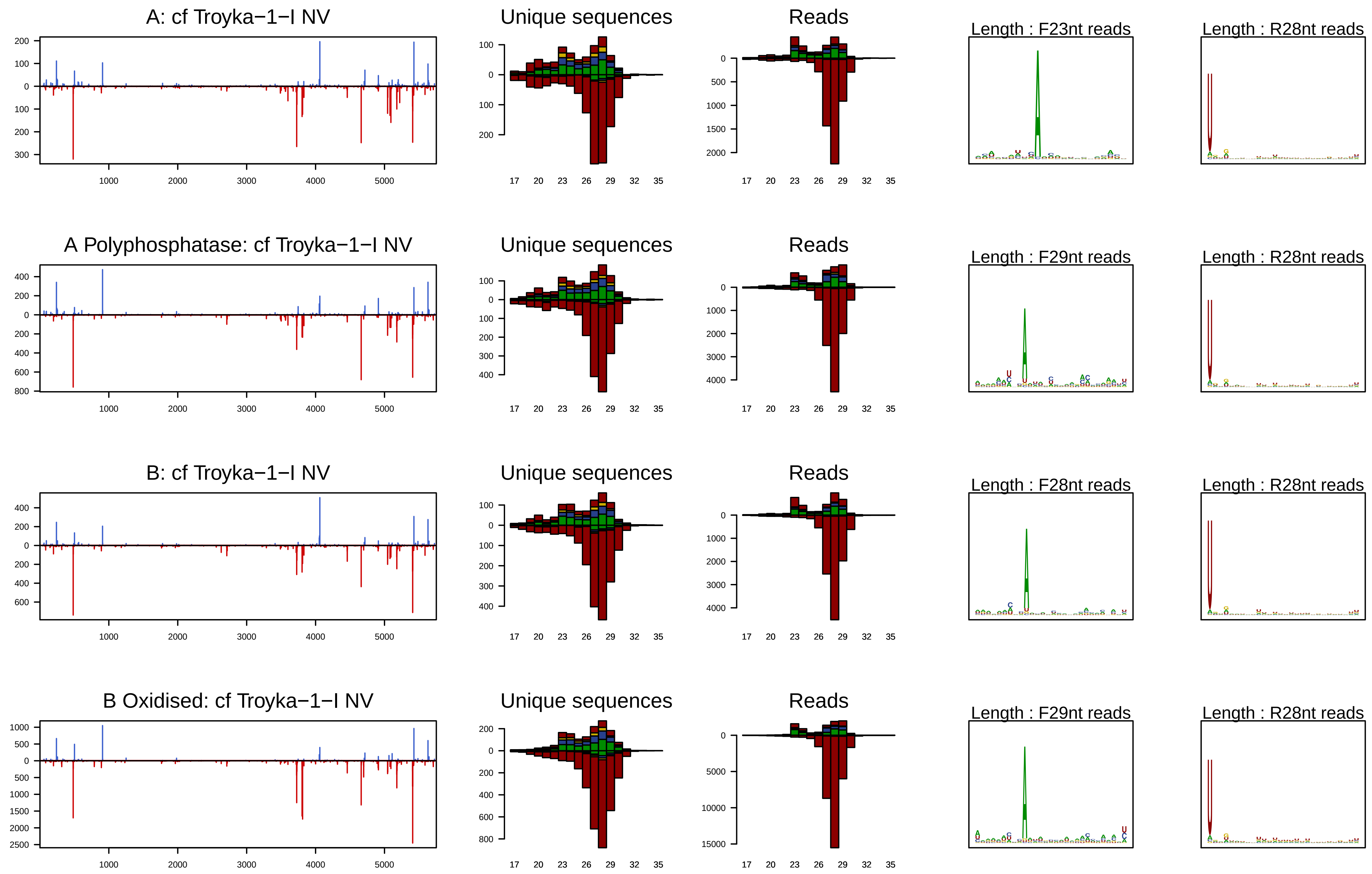

O: Sea Anemone DN69209 cf. Gypsy−16−I NV

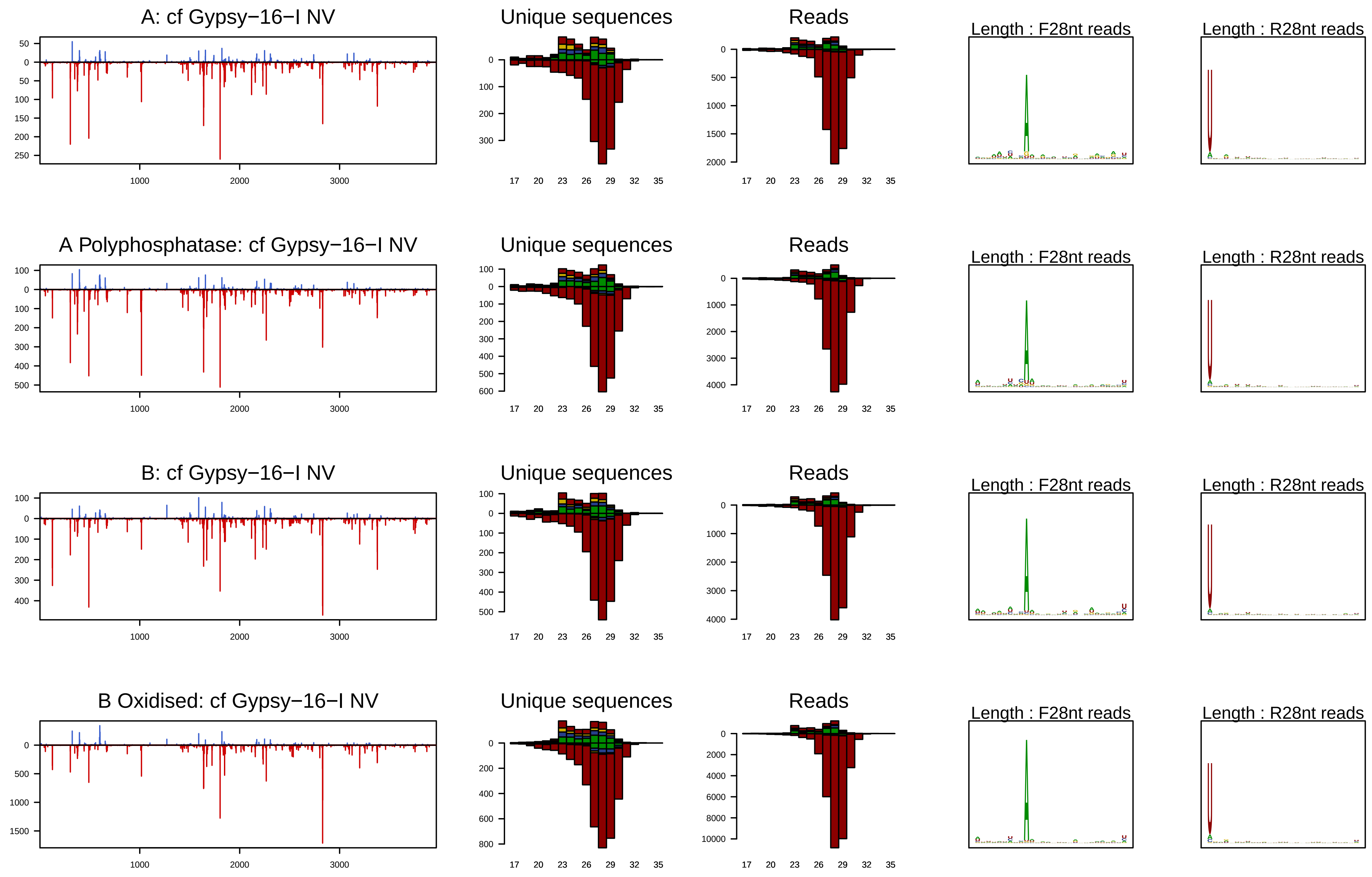

P: Fucus DN74132 cf. Copia−7 ES−I Copia Ectocarpus siliculosus

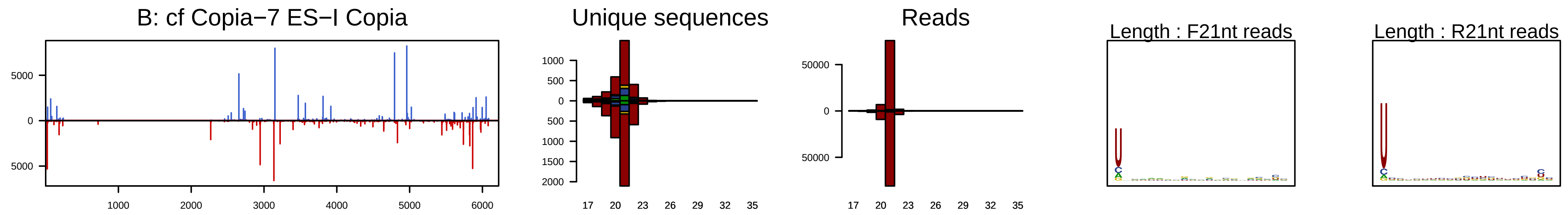

Q: Fucus DN73655 cf. Copia−1 ES−I Copia Ectocarpus siliculosus

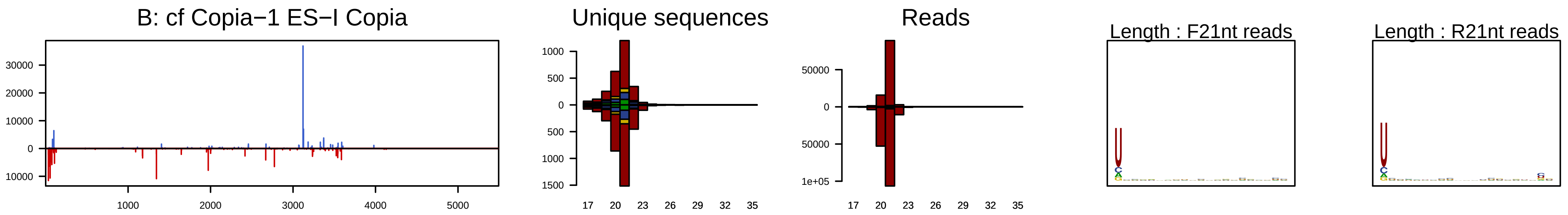

R: Fucus DN73624 cf. RTE−14 Lch RTE Latimeria chalumnae

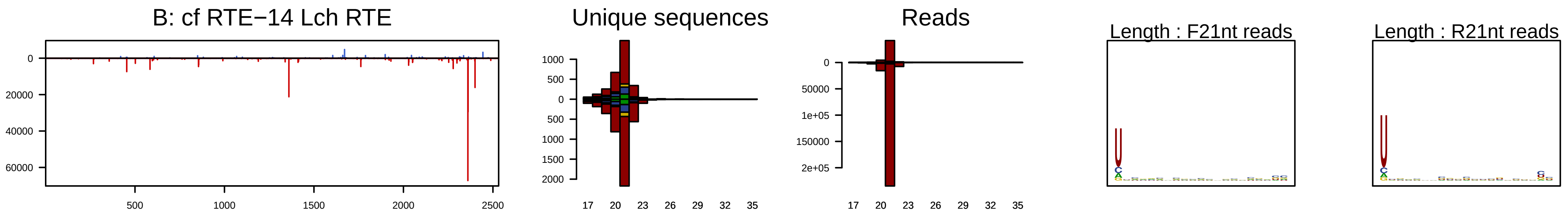

Supplement: S8 Fig — Panels A-R show the small RNA properties of selected high-confidence TE-like contigs: starfish panels A-C, dog whelk D-F, sponge G-I, earthworms J-L, sea anemone M-O, brown alga P-R. Rows and columns are as in S6 Fig. The data required to plot the size distributions are provided in S5 Table. (PDF) [file pgen.1007533.s008.pdf]
