# Supplementary material for: Metagenomic sequencing suggests a diversity of RNA interference-like responses to viruses across multicellular eukaryotes
Source: PLoS Genet. 2018 Jul 30;14(7):e1007533. doi: 10.1371/journal.pgen.1007533 (PMC6085071; doi:10.1371/journal.pgen.1007533)
Supplement: S1 Text — Detailed description of the sampling, tissue preparations and RNA extractions techniques employed for each target taxon. (PDF) [file pgen.1007533.s021.pdf]

S1_Doc Sampling, tissue processing and RNA extractions (Waldron *et al.*, 2017)

Sample collections

All target taxa were sampled in Scotland, UK, in 2014 and 2015. For marine species, individuals of several target taxa were sampled from rock pools and the rocky shore at Barns Ness (Dunbar, East Lothian: 55.988° N, -2.445° W) at low tide on 11/7/14. Marine target taxa were also sampled on a separate occasion from 3 locations near Millport on the island of Great Cumbrae (North Ayreshire). The 3 sites were Ballockmartin Bay (55.768° N, -4.898° W), Farland Bight (55.746° N, -4.911° W), and Kames Bay (55.754° N, -4.914° W) with sampling carried out at low tide on 11-13/8/14. All marine taxa sampled were stored separately prior to laboratory processing, to avoid potential viral cross-contamination between target taxa. Our single terrestrial target taxon, earthworms (of several spp.) were sampled from the soil at the King's Buildings, University of Edinburgh (55° 55' 17.7" N, 3° 10' 35.4" W) on 2-3/11/15.

Tissue processing and RNA extraction: General principles

Tissue excisions were performed using pre-prepared, sterilized, and DEP-C treated razor blades and/or scissors, and tissue cleaning procedures carried out as quickly as possible in dishes kept on ice to avoid RNA degradation (see Riesgo, Andrade, et al., 2012; Riesgo, Pérez-Porro, et al., 2012). To minimise potential cross-contamination, no dissection instruments or dishes were shared between sample pools. Similarly, separate pre-prepared (sterilised and rinsed in RNAseZap (Thermo Scientific)) mortar and pestle set was used for the majority of sample pools. In a minority of cases, where mortar and pestle sets were reused between pools, these were first sets were carefully washed, sterilised and rinsed with RNAseZap.

Below we list sampling techniques, tissue processing, and RNA extraction as they relate to each target taxon.

Halichondria panicea, "the breadcrumb sponge" (Porifera: Demospongiae)

Sampling

Samples of *Halichondria panacea* colonies approximately 1-2cm square were removed from rocks in and around marine rock-pools, and stored in sea water at ambient water temperature on site and at 4°C in the laboratory for up to 12 hours before tissue processing.

Tissue processing and RNA extraction

To remove epibionts and macroscopic debris, small (≤0.5 mm^3) pieces of tissue were excised from each individual colony and cleaned under a stereomicroscope (see Conaco et al., 2012; Riesgo, Andrade, et al., 2012; Riesgo, Pérez-Porro, Carmona, Leys, & Giribet, 2012). Immediately after cleaning, each tissue piece was flash frozen in liquid nitrogen then placed on dry ice, with 11-15 colonies represented in each sample pool. Pools were then pulverised to a fine powder under liquid nitrogen using a mortar and pestle (Gayral et al., 2011). Pulverised frozen tissue (~100-200 mg) was placed in eppendorfs with 1 ml of Trizol (Life Technologies), and then stored at -80°C for later RNA

extraction. All samples were processed within 12 hours of initial collection, and RNA extractions (Trizol) were according to the manufacturer's instructions.

***Actinia equina*, "the beadlet anemone" (Cnidaria: Anthozoa)**

Sampling

Anemones were separated from their underlying rocky substrate, and stored in sea water at ambient water temperature on site, and at 4°C in the laboratory for up to 48 hours before processing.

Tissue processing

Small (≤9 mm^3) pieces of tissue (encompassing outer wall, tentacle, oral disc and mouth tissues) were excised from the oral end of the anemone column, taking care to exclude the central pharynx and greater digestive tract. After removing excess water from anemone tissues by blotting on filter paper (see Stefanik, Wolenski, Friedman, Gilmore, & Finnerty, 2013), each tissue piece was immediately flash frozen in liquid nitrogen and placed on dry ice, with 14-20 individuals represented in each sample pool. Tissue pulverisation and storage in Trizol protocols were carried out as described for *Halichondria panicea* (above), with sample pools numbers 1-7 (from Barns Ness), and numbers 8-14 (from Millport) processed within 12 hours, and 48 hours of field sampling, respectively. RNA extraction (Trizol) was carried out following manufacturer's instructions incorporating published protocols for sea anemones; *Actinia equina* (Moran *et al.*, 2008), and *Nematostella vectinensis* (Stefanik *et al.*, 2013).

***Asterias rubens*, "the common starfish/sea star" (Echinodermata: Asteroidea)**

Sampling

Starfish were hand-collected from rock-pools. Starfish sampled from Millport were stored in sea water at ambient water temperature prior to tissue processing within hours of sampling (see below). Starfish sampled from Barns Ness were stored in sea water at ambient water temperature on site, and at 4°C in the laboratory in Edinburgh for up to 48 hours before tissue processing.

Tissue processing

To avoid sampling digestive tract and reproductive tissues, small amounts of tissue (<5 mm) were harvested from the tips (to avoid digestive caecae) of each of two arms of each starfish. Starfish sampled at Millport (*n=197*; sample pools 2-13) were processed at a nearby field station (Field Studies Council Millport Field Centre), and were returned to the collection location, within 6 hours. After excision, tissue pieces were immediately flash frozen in liquid nitrogen and then placed on dry ice, with 7-30 individuals represented in each sample pool. Tissue pulverisation and storage in Trizol protocols were carried out as described for *Halichondria panicea* (see above), with sample pools 2-13 (from Millport), and 1 (from Barns Ness) processed within 12 hours, and 24 hours of field sampling, respectively. RNA extraction (Trizol) was carried out following manufacturer's instructions, as published previously for *Asterias rubens* (Leclerc *et al.*, 2016).

***Nucella lapillus*, "the dog whelk" (Mollusca: Gastropoda)**

Sampling

Dog whelks were collected from marine rock-pools and adjacent rocks, and stored in sea water at ambient water temperature on site, and at 4°C in the laboratory for up to 72 hours before tissue processing.

Tissue processing

To avoid sampling reproductive and gut tissues, we excised a small amount of tissue from the head. Dog whelk shells were broken using a benchtop vice, and using scissors a small (≤0.5 mm^3) piece of tissue encompassing the anterior head, tentacles, and foot were excised, avoiding any portion of digestive tract, including tissues immediately surrounding the buccal cavity. Tissue pieces were immediately flash frozen in liquid nitrogen and immediately placed on dry ice, with 14-20 individuals represented in sample pool. Tissue pulverisation and storage in Trizol protocols were carried out as described for *Halichondria panicea* (see above), with sample pools 1-7 (from Barns Ness), and 8-11 (from Millport) processed within 24 hours, and 78 hours of field sampling, respectively. RNA extraction (Trizol) was carried out following manufacturer's instructions, as published previously for several mollusc classes (Pérez-Portela & Riesgo, 2013) including gastropods (Gayral *et al.*, 2011).

***Lumbricus and Amynthas spps.*,"earthworms" (Annelida: Oligochaeta)**

Sampling

Earthworms were collected from exposed soil, and were stored at ambient air temperature on site and at 4°C in the laboratory for up to 48 hours before tissue processing.

Tissue processing

Following published protocol (Riesgo, Pérez-Porro, *et al.*, 2012) earthworms were first cleaned carefully to remove epibionts and macroscopic debris prior to tissue processing. From each earthworm, a portion of tissue encompassing the first 4-8 anterior segments (containing prostomial, central ganglia and pharynx) was then harvested. We note that segments 1-8 do not contain reproductive tissues, but do contain digestive tract (buccal cavity, pharynx, and oesophagus). Upon dissection however, digestive tract was found to be empty, as earthworms had been stored for 24 hours prior to tissue processing. Tissue pieces were immediately flash frozen in liquid nitrogen and placed on dry ice), with 8-16 individuals represented in each sample pool. Tissue pulverisation and storage in Trizol protocols were carried out as described for *Halichondria panicea* (above), with all sample pools processed within 78 hours of field sampling. RNA extraction (Trizol) was carried out following manufacturer's instructions, as published previously for oligochaetes (Riesgo, Andrade, *et al.*, 2012).

***Fucus serratus*, "the serrated wrack" (Heterokonta: Phaecophyceae: Fucales)**

Sampling

Whole fronds of small/medium sized individual *Fucus* were removed from marine rock-pools and adjacent rocks, and stored in sea water at ambient water temperature on site, and at 4°C in the laboratory for up to 48 hours before tissue processing.

Tissue processing

Single pieces of lamina (~1 cm^2) were excised using, taking care to exclude visible reproductive bodies. *Fucus serratus* fronds support a wide range of mutualists and other epibionts (Fish & Fish, 2011), so tissue pieces were first washed in sieved seawater to remove macro-invertebrates before being flash frozen in liquid nitrogen. These were stored as sample pools at -80°C. Five sample pools (each containing tissues from 20 individuals, i.e. 100 individuals) were pulverised together using a mortar and pestle.

After a large number of unsuccessful attempts (protocols available from the authors on request), RNA was extracted using the following protocol (a modified version of a published protocol for nucleic acid isolation in the brown alga, *Macrocytis pyrifera* (Apt *et al.*, 1995) kindly provided to us by Susana Coelho:

***Fucus serratus* RNA extraction protocol**

Day 1

Sample preparation and suspension in extraction buffer

- Grind algae to fine powder in liquid nitrogen using a mortar and pestle. Grind for at least ten minutes – this step is essential for final performance.
- Transfer the powder into a cooled 1.5 ml eppendorfs and add frozen algae powder to extraction buffer (see below for buffer) at a ratio of 1:10 (weight g/vol. ml). For 1.5ml eppendorfs: 75mg to 750ul extraction buffer.
- Shake vigorously for at least 15 minutes at room temperature.

Extraction buffer:
100 mM Tris-HCl pH 7.5 (pH 8; Apt et al.)
1.5M NaCl
2% CTAB (added to EB immediately before use)
50 mM EDTA, pH 8
50 mM DTT freshly added to the buffer (buffer usable for 2 days after adding DTT)

Chloroform/isoamyl alcohol Extraction 1

- Add 1 volume chloroform/isoamyl alcohol (24:1)
- Centrifuge at 10,000 g for 15 mins.
- Transfer the upper aqueous phase into a fresh 1.5ml eppendorf.
- Precipitate polysaccharides by adding 0.3 volumes of 100% ethanol.

Chloroform/isoamyl alcohol Extraction 2

- Repeat as for extraction 1

Precipiation with LiCl and β-mercaptoethanol

- Add ¼ volume 12M LiCl and 1% v/v β-mercaptoethanol.
- Stir vigorously.

- Precipitate overnight at -20 ° C

Day 2

Dissolve precipitated RNA in TE

- Centrifuge sample at 10,000 g for 60 mins at 4°C.
- Remove supernatant and resuspend pellet in 750ul TE (see below for TE).

TE:
Tris-HCl pH 8, 10 mM
1mM EDTA

Phenol:chloroform extraction

- Add an equal volume (1:1) of phenol-chloroform (pH 4.3 for RNA).
- Centrifuge at 10,000 g for 15 mins.
- Transfer the aqueous phase into a fresh 1.5ml eppendorf

Overnight precipiation 100% EtOH

- Add 2.5 volumes cooled ethanol and 0.1 volume NAOAc (3M, pH 5.2).
- Precipitate overnight at -20 ° C.

Day 3

Dissolve precipitated RNA and store in nuclease-free H_20

- Centrifuge at 10,000 g for 30 mins at 4°C.
- Re-suspend in nuclease-free H_20
- Store at at -80° C.

References

Apt, K., Clendennen, S., Powers, D. & Grossman, A. 1995. The gene family encoding the fucoxanthin chlorophyll proteins from the brown alga *Macrocystis pyrifera*. *Mol. Gen. Genet.* **246**: 455–464.

Conaco, C., Neveu, P., Zhou, H., Arcila, M.L., Degnan, S.M., Degnan, B.M., *et al.* 2012. Transcriptome profiling of the demosponge *Amphimedon queenslandica* reveals genome-wide events that accompany major life cycle transitions. *BMC Genomics* **13**: 209.

Fish, J. & Fish, S. 2011. *A Student's Guide to the Seashore*, 3rd Ed. Cambridge University Press.

Gayral, P., Weinert, L., Chiari, Y., Tsagkogeorga, G., Ballenghien, M. & Galtier, N. 2011. Next-generation sequencing of transcriptomes: a guide to RNA isolation in nonmodel animals. *Mol. Ecol. Resour.* **11**: 650–61.

Leclerc, M., Kresdorn, N. & Horres, R. 2016. *Asterias rubens*: Evidence of NF-kappa B genes. *Meta Gene* **8**: 30–32.

Moran, Y., Weinberger, H., Sullivan, J.C., Reitzel, A.M., Finnerty, J.R. & Gurevitz, M. 2008. Concerted evolution of sea anemone neurotoxin genes is revealed through analysis of the *Nematostella vectensis* genome. *Mol. Biol. Evol.* **25**: 737–747.

Pérez-Portela, R. & Riesgo, A. 2013. Optimizing preservation protocols to extract high-quality RNA from different tissues of echinoderms for next-generation sequencing. *Mol. Ecol. Resour.* **13**: 884–889.

Riesgo, A., Andrade, S.C.S., Sharma, P.P., Novo, M., Pérez-Porro, A.R., Vahtera, V., *et al.* 2012. Comparative description of ten transcriptomes of newly sequenced invertebrates and efficiency estimation of genomic sampling in non-model taxa. *Front. Zool.* **9**: 33.

Riesgo, A., Pérez-Porro, A.R., Carmona, S., Leys, S.P. & Giribet, G. 2012. Optimization of preservation and storage time of sponge tissues to obtain quality mRNA for next-generation sequencing. *Mol. Ecol. Resour.* **12**: 312–22.

Stefanik, D.J., Wolenski, F.S., Friedman, L.E., Gilmore, T.D. & Finnerty, J.R. 2013. Isolation of DNA, RNA and protein from the starlet sea anemone *Nematostella vectensis*. *Nat. Protoc.* **8**: 892–9.
