# Supplementary material for: Metagenomic sequencing suggests a diversity of RNA interference-like responses to viruses across multicellular eukaryotes
Source: PLoS Genet. 2018 Jul 30;14(7):e1007533. doi: 10.1371/journal.pgen.1007533 (PMC6085071; doi:10.1371/journal.pgen.1007533)
Supplement: S2 Text — Protocol for sodium periodate (NaIO4) oxidation of RNA prior to library preparation, to enrich small RNA libraries for canonical piRNAs and viRNAs by reducing the relative ligation efficiency of metazoan miRNAs that lack 3′-Ribose 2′O-methylation. (PDF) [file pgen.1007533.s022.pdf]

Oxidation protocol for RNA (Waldron *et al.*, 2017)

Background:

Oxidation using reduces the relative ligation efficiency of animal miRNAs that lack 3′-Ribose 2′O-methylation, relative to canonical piRNAs and viRNAs. This permits identification of 3′- 2′O-methylated sRNA populations, and is expected to enrich small RNA library for canonical piRNAs and viRNAs.

Protocol:

Start with RNA dissolved in nuclease-free H_20 (20 µl)

1. Combine RNA, 8 µl 5X Borate Buffer (pH 8.6), 5 µl 200mM $NaIO_4$ (Sodium periodate), and nuclease-free H_20 up to a final volume of 40 µl.
2. Incubate at 25 °C for 30 min.
3. Add: 229 µl Nuclease-free H_20, 30 µl 3M NaoAc (pH 5.2), 1 µl of 20 µg/µl glycogen up to final volume of 300 µl.
4. Add 900 µl of 100% EtOH and incubate overnight at -80 °C
5. Spin at 13,000 rpm for 30 min at 4 °C
    a. Remove supernatant and wash pellet with 1 ml of 80% EtOH
6. Spin at 13,000 rpm for 1 min at 4 °C
    a. Remove supernatant and residual EtOH
7. Air dry pellet for 10 min and dissolve in 12 µl of nuclease-free H_20

……….
